# Hydrogen Radical Chemistry at High-Symmetry {2Fe2S} Centers Probed Using a Muonium Surrogate

**DOI:** 10.1021/acs.inorgchem.4c05126

**Published:** 2025-03-01

**Authors:** Joseph A. Wright, Farhana Haque, Leandro Liborio, Stephen P. Cottrell

**Affiliations:** † Energy Materials Laboratory, School of Chemistry, Pharmacy and Pharmacology, University of East Anglia, Norwich Research Park, Norwich NR4 7TJ, U.K.; ‡ Scientific Computing Department, Science & Technology Facilities Council, Rutherford Appleton Laboratory, Harwell Science Campus, Didcot, Oxfordshire OX11 0QX. U.K.; § ISIS Facility, Science & Technology Facilities Council, Rutherford Appleton Laboratory, Harwell Science Campus, Didcot, Oxfordshire OX11 OQX, U.K.

## Abstract

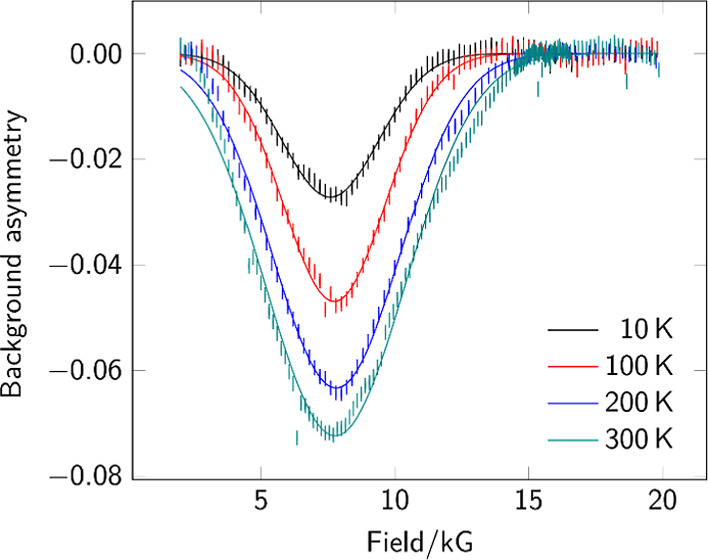

Redox-active metal hydrides are of central importance in the development of novel hydrogen generation catalysts. Direct insight into open-shell hydrides is, however, difficult to obtain. One approach to gain this information is to use muonium (Mu^•^ = μ^+^ e^–^) as a surrogate for the hydrogen radical. The chemistry of Mu^•^ is analogous to H^•^; however, the species provides a highly sensitive probe through detection of the positrons arising from the muon decay (with a lifetime of ∼2.2 μs) and can therefore provide unique information about hyperfine couplings and thus molecular structure. Using this approach, we demonstrate here that the high-symmetry {2Fe2S} systems Fe_2_(edt)(CO)_4_L_2_ (edt = ethane-1,2-dithiolato; L = CO, PMe_3_, CN^–^) form bridging radicals directly on the time scale of the muon experiment. We also extend our computational approach to detail all of the possible addition sites in solid state samples.

## Abstract 2

Muonium (Mu^•^ = μ^+^ e^−^) can be used as a surrogate for the hydrogen radical, with decay of the muon allowing probing of sites of hydride formation without needing access to closed-shell precursors. Here, we use this approach to examine high-symmetry {2Fe2S} systems which form bridging radicals directly on the time scale of the muon experiment. By extending our modeling approach, we can scale this analysis to a wide range of significant organometallic systems.

## Introduction

Models of the [FeFe]-hydrogenase active site continue to attract attention due to their attractive properties of the enzyme system.^1,2^ The enzyme family offers high turnover for the production of H_2_ and is well established to be as efficient as platinum when measuring on a molar basis.^3^ The challenges of working with whole enzymes, including air sensitivity and high molar mass and volume, mean that the search for viable catalysts based on mimicking the active sites continues to be an area of significant research. The exquisite control of redox potentials exhibited in the natural system remains a grand challenge and drives both technological development and intellectual curiosity.^4^

A key aspect of this work is obtaining new insight into the behaviors of metal hydride systems that are central to hydrogen evolution catalysis. Probing the paramagnetic states formed when both a proton and an electron are added to isolable diamagnetic systems remains a challenge. Preforming metal hydrides followed by electron transfer can be used in preparation for electron paramagnetic resonance spectroscopy but is limited to kinetically stable hydrides. Protonation of reduced species is even more challenging as the open-shell species typically have very limited lifetimes.

An attractive route for the direct formation of open shell hydride mimics is the use of positive muons. When muons are stopped in materials, muonium radicals (Mu = μ^+^ e^–^) form by acquisition of electrons creating a species chemically equivalent to a hydrogen atom but with lower mass and limited lifetime (∼2.2 μs).^5,6^ Crucially, this species provides a highly sensitive probe through detection of positrons arising from muon decay. Key to our experiments is that muons are produced almost 100% spin polarized, and this spin can be affected using appropriate external magnetic fields. Potential muon implantation sites can be determined by using an appropriate combination of experimental and simulation data. A set of sustainable software tools, based upon density functional theory (DFT) simulations, have been recently developed to help with the interpretation of muon experiments.^7−9^

There are a number of related muon spectroscopy techniques; however, for chemical application, the most useful is the avoided level crossing muon spin resonance (ALC-μSR) experiment, in which a longitudinal field is applied to the sample being examined.^10^ In the solid state, strong Δ_1_ resonances are expected, where only the muon spin changes sign and the resonance field is proportional to the muon hyperfine coupling.

**Figure 1:**
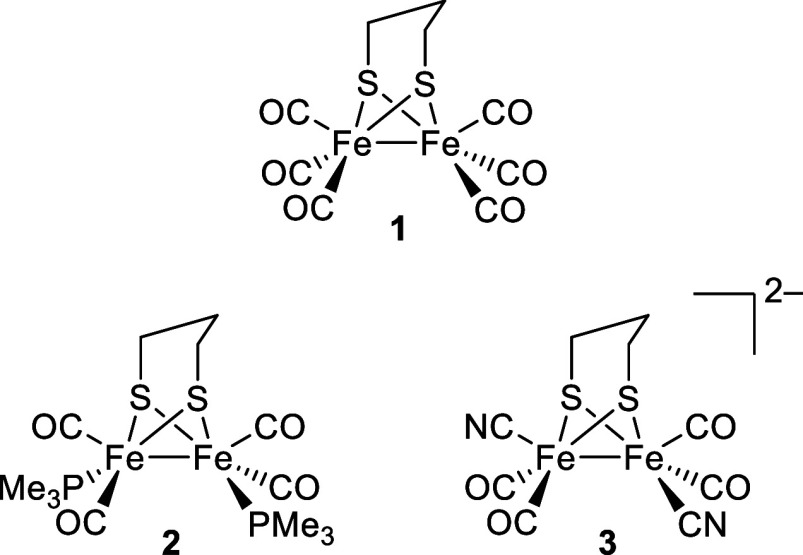
{2Fe2S} complexes containing a three-carbon dithiolate bridge.

We have previously described the use of ALC-μSR to probe hydride chemistry at metallosulfur complexes **1**–**3** (Figure 1), allowing us to examine the direct generation of paramagnetic states featuring a hydride surrogate.^11^ This report was the first using muons in redox-active organometallics and is one of only a small number which examine organometallic systems using muon chemistry.^12−16^ We were able to establish that muoniated radicals bound to the metal centers were amenable to ALC-μSR, and that the majority of muoniation occurred at a single site.

**Figure 2:**
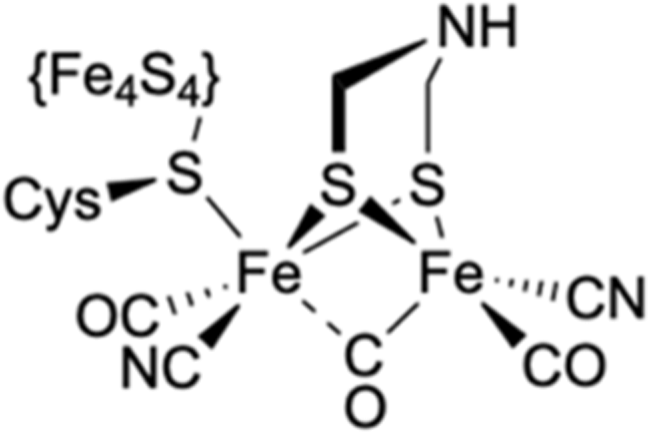
[FeFe]-hydrogenase enzyme active site.

The enzyme active site features a three-atom bridge between the two sulfur centers with a central nitrogen atom acting as a proton relay (Figure 2). Complexes **1**–**3** feature a three-carbon (propane-1,3-dithiolato, pdt) bridge, which is the same length as that in the enzyme but more synthetically accessible. However, the central atom breaks the apparent symmetry of the systems, making data analysis more challenging in the solid state. In particular, this complicates the DFT simulation of additions sites: the central carbon of the bridge sits over one iron center, and both variants have to be considered to fully explore the range of muoniation sites. The DFT approach used previously,^11^ simulating gas phase structures using hydride then postprocessing to account for the muon size and magnetogyric ratio, was also labor-intensive and difficult to automate. Here, models with higher molecular symmetry were examined to allow development of more scalable DFT approaches and to investigate the influence of bridge length on muoniation outcomes.

## Results and Discussion

**Figure 3:**
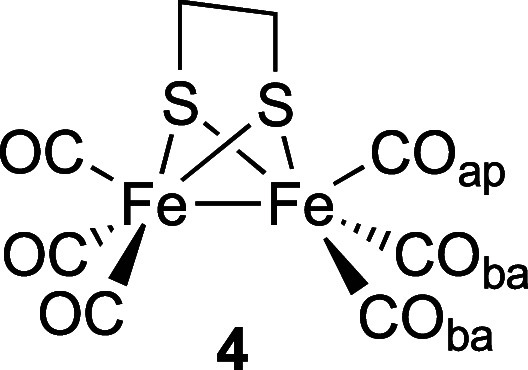
High-symmetry {2Fe2S} complex containing a two-carbon dithiolate bridge highlighting the apical (ap) and basal (ba) positions.

Complex **4** (Figure 3), which contains a symmetrical two-carbon (ethane-1,2-dithiolato, edt) bridge, is readily available in one step from commercial material following the same synthetic route as that for complex **1**. As this has far fewer potential sites for muon addition, we reasoned that it could be used to confirm the previous assignment of the muon addition site while perhaps giving stronger resonances given the limited number of final state species that can be formed. The latter is particularly attractive when considering more challenging experiments for direct observation of the hyperfine interaction.

**Figure 4:**
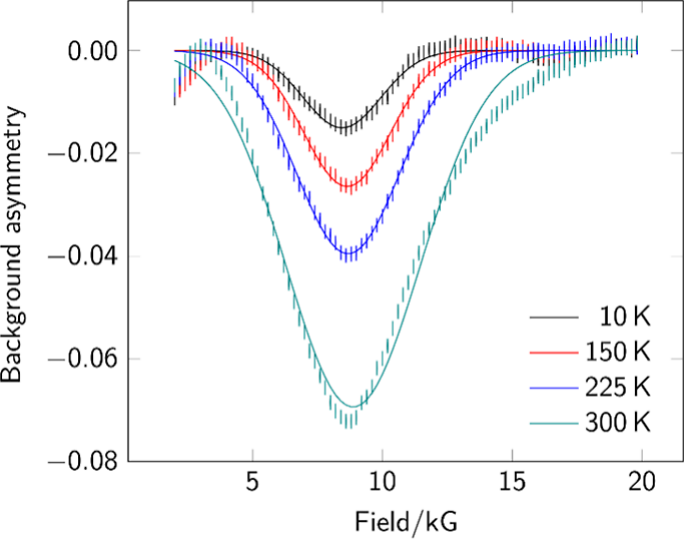
Background-subtracted ALC-μSR spectra for complex **4**. Data points are shown as sticks representing the estimated uncertainty in each point. Gaussian fits are shown as superimposed lines.

ALC-μSR data for complex **4** as a powder were obtained across a range of temperatures, and after background subtraction could be fitted with a single Gaussian peak centered at around 8.5 kG (Figure 4). The choice of a Gaussian function was made to allow parametrization of the peaks measured and does not reflect any particular model for the underlying physics. This line shape and position is broadly in accord with the data obtained previously for complexes **1**–**3**. The position of the resonance peak shows a weak temperature dependence, suggesting a small increase in *A*_Mu_ as the temperature is increased to 300 K.

**Figure 5:**
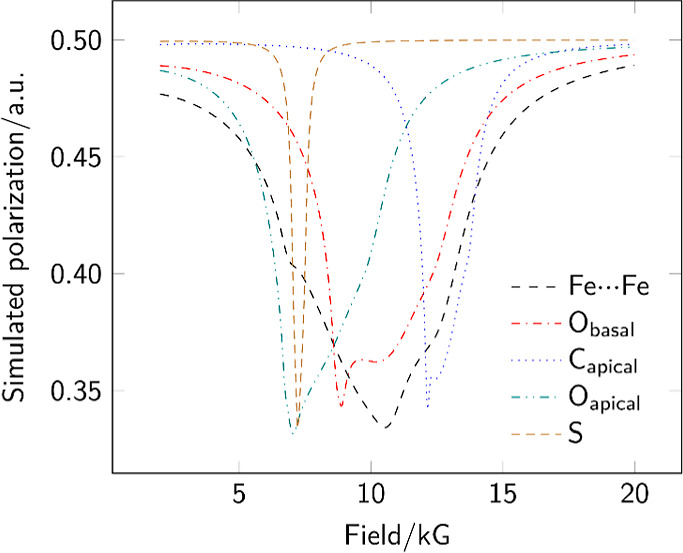
MuSpinSim simulated powder ALC-μSR spectra for each implantation site in complex **4** yielding a resonance in the range 2 to 20 kG; the basal carbon site gave a resonance well above 30 kG and is omitted from the plot.

To properly investigate the high-symmetry environment around the implanted muonium, the potential addition sites were simulated using the CASTEP^17^ code, which allows for treatment of potential intermolecular interactions that may impact both the placement of the implanted muon and the resulting hyperfine values. The use of CASTEP allows for the unique properties of the muon to be included in the simulation, with both its mass and magnetogyric ratio selectable as part of the initial parameter set. Viable muoniation sites were found as expected at the midpoint of the metal–metal bond, at both the oxygen and carbon atoms of the two carbonyl positions, and at the sulfur. The energies of these implantation sites varied by around 175 kJ mol^–1^ with the bridging site most favorable and oxygen binding least favorable. After calculation of the three-dimensional hyperfine tensor for all structures, powder ALC-μSR spectra were simulated using the MuSpinSim code (Figure 5).^18−20^ With the exception of the basal carbon atom, all of the muoniation sites gave resonance maxima in the range 6–12 kG. While the shape of the simulated Fe−μ–Fe site is in accordance with the experimental results, the overlap of potential signals meant that we sought additional experimental evidence to confirm the assignment.

**Figure 6:**
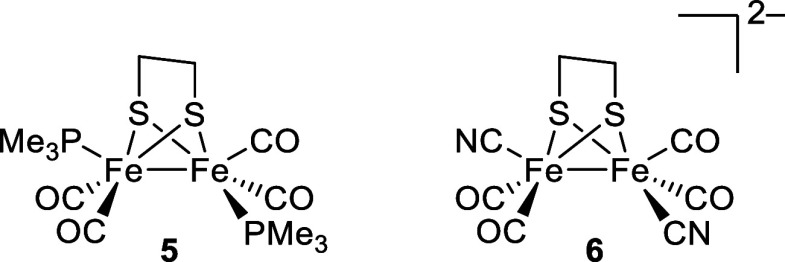
Electron-rich {2Fe2S} complexes containing two-carbon dithiolate bridge.

Substitution of one carbonyl at each metal in complex **4** by either a trimethylphosphine or cyanide can be carried out readily, to give complexes **5** and **6**, respectively (Figure 6). The replacement of two carbonyl ligands by either PMe_3_ or CN^–^ results in more electron-rich molecules showing significantly shifted IR bands.^21−24^ While these retain symmetry of the Fe_2_(edt) core, they adopt lower-symmetry molecular structures in the solid state, as in both systems one noncarbonyl ligand is apical while the other is basal (see Figure 3). This means that for complexes **2** and **3**, there are several potential muoniation sites but without the subtle challenges introduced by the three-carbonyl bridge.

**Figure 7:**
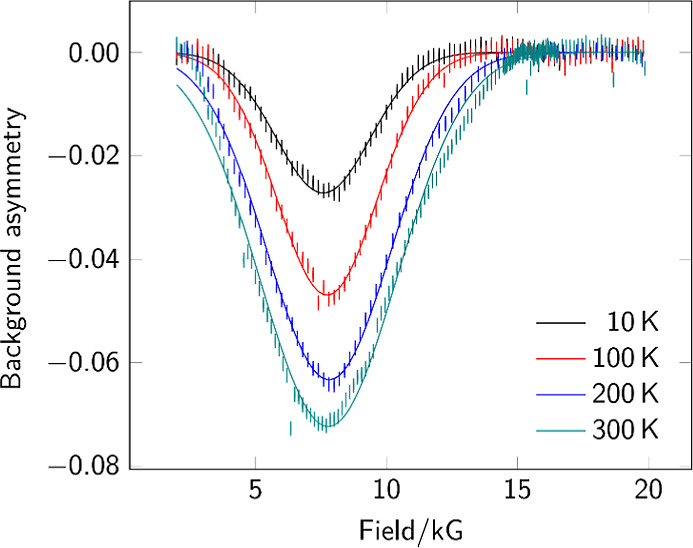
Background-subtracted ALC-μSR spectra for **5**. Data points are shown as sticks representing the estimated uncertainty in each point. Gaussian fits are shown as superimposed lines.

**Figure 8:**
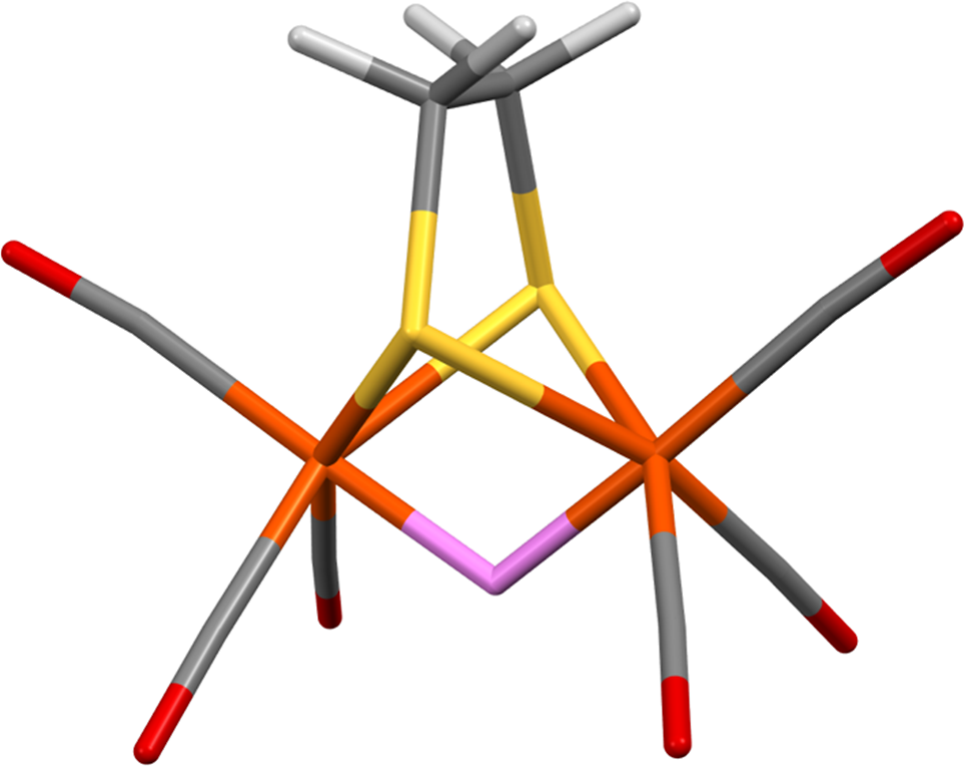
Stick representation of the Fe−μ–Fe site in **4**; the location of the muonium is after energy minimization in CASTEP. Color scheme: muonium, pink; hydrogen, white; carbon, gray; oxygen, red; sulfur, yellow; and iron, orange.

**Figure 9:**
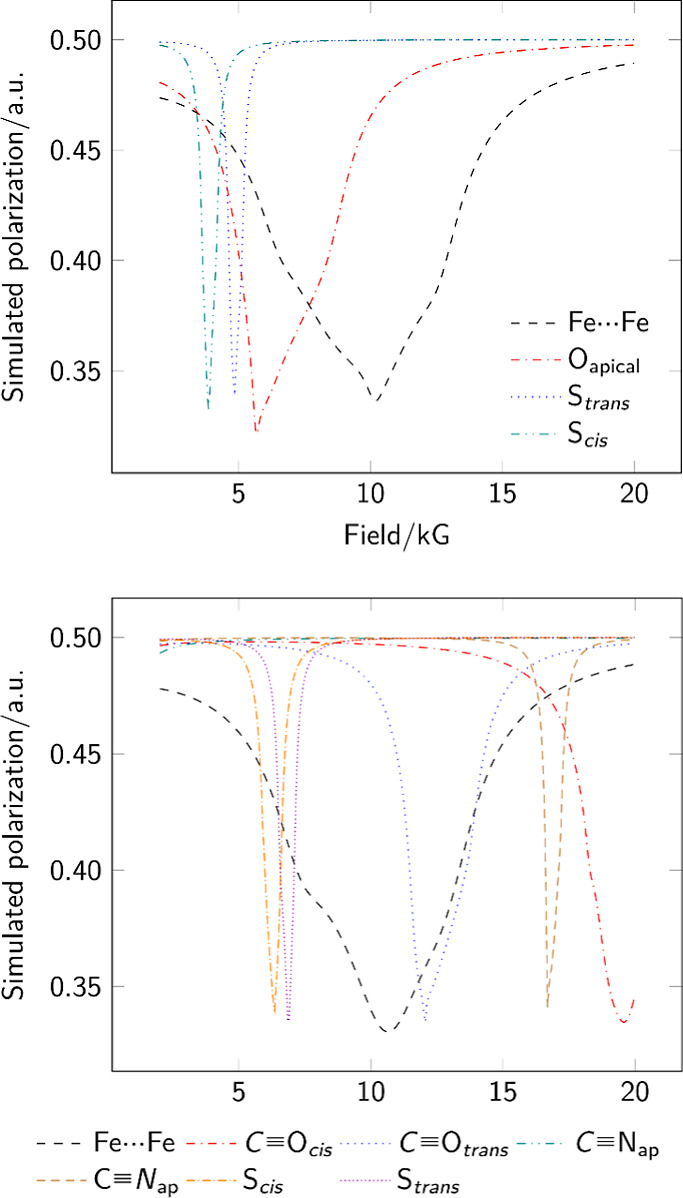
MuSpinSim-simulated ALC-μSR spectra for each implantation site in **5** (top) and **6** (bottom) yielding a resonance in the range 2 to 20 kG. In both cases, other oxygen-based muoniation sites do not yield energy minima while carbon-bound sites give resonances above 20 kG. The labels cis and trans describe the relative geometry of the muoniation site and the unique basal ligand (PMe_3_ or CN^–^).

Solid-state ALC-μSR spectra for complexes **5** and **6** (Figure 7 and Supporting Information Figure S1, respectively) show similar forms to that for complex **4**: one broad signal shifted in these more electron-rich systems to slightly lower field. DFT simulations were carried out for the full set of potential muoniation sites in both of these molecules: the center of the metal–metal bond (Figure 8), each unique carbonyl site, each sulfur, and for complex **6**, each end of each cyanide ligand. Only a small number of the sites yielded viable addition sites, giving resonances in the relevant range (2–20 kG) (Figure 9). Addition to most of the carbonyl oxygen atoms did not result in viable energy minima. As for complex **4**, the energies for successful implantation varied over a range of around 100 kJ mol^–1^ and were not sufficient to rule out any sites. The resonance positions obtained for the carbonyl carbon atoms were all well above 20 kG. For all three complexes, muoniation at the sulfur atom(s) gave sharp resonances in the region of 6 kG. Only the formation of the Feμ–Fe state consistently gave a broad signal falling close to the experimentally observed position.

The weak temperature dependence of the resonance position noted for **4** was also observed in complexes **5** and **6**. Although not central to the present study, the trend in peak intensities with temperature appears similar to results previously reported in ref (11). Sufficient data were available for complex **4** to confirm an Arrhenius dependence with a comparable activation energy, *E*_a_, estimated to be 2.3(3) kJ mol^–1^ (Figure S2). For complexes **5** and **6**, the number of temperature points available precludes quantitative analysis of the addition barrier.

The CASTEP DFT values for the Fermi contact terms of the Fe−μ–Fe adducts of complexes **4**, **5**, and **6** are 278.6713 MHz (10.23 kG), 266.9844 MHz (9.80 kG), and 282.0291 MHz (10.35 kG), respectively. These simulation values of the Fermi contact terms can be used to predict the location of the ALC-μSR peak and further interpret the ALC-μSR experiments. Finally, the full ALC-μSR signal also depends on the off-diagonal terms of the hyperfine tensor, which are responsible for the shape of the ALC-μSR peak. As it can be seen in the Support Information, the CASTEP off-diagonal terms obtained for the hyperfine tensors of complexes **4**, **5**, and **6**, with muonium implanted in the FeμFe site have values significantly larger that when the muon is implanted in all the other proposed sites. These large values for the off-diagonal terms arise because the simulations are able to represent the asymmetric effects caused by the environment around the implanted muonium (metal d electrons in close proximity) and therefore produce simulated ALC-μSR signals that are closer to the experimental results, as can be observed in Figures 5 and 9.

## Conclusions

Muonium implantation at Fe_2_(edt)(CO)_4_L_2_ species proceeds with the formation of a single state characterized by a broad resonance at around 8.5 kG. Simulation of powder ALC-μSR spectra for the full range of potential sites in the solid state can be achieved using CASTEP and MuSpinSim. This confirms exclusive formation of the Fe−μ–Fe product, consistent with the previous study. These results will allow the ALC-μSR to be applied to {2Fe2S} systems featuring a richer ligand set, targeting systems bearing multidentate phosphine ligands and/or known to form terminal hydrides. Future publications from our group will explore these systems in due course.

The combination of CASTEP and MuSpinSim allows for a detailed examination of not only the position but also the shape of the resonances obtained, which is significant in assigning the very broad signals obtained from ALC-μSR of organometallic species. The results presented here therefore can be expected to act as a firm basis on which to probe a wider variety of more challenging organometallic hydride species, with certainty concerning the reactivity of muonium and the simulation of putative addition sites. Finally, these simulations can assist with ALC-μSR experimental planning as the simulated ALC-μSR center can be used to determine the region of the magnetic field to scan in an ALC-μSR experiment, saving valuable experimental time.

## Experimental Section

Compounds **4**, **5**, and **6** were prepared by literature procedures as previously described.^25−27^ Avoided level crossing muon spectroscopy was carried out using the HiFi beamline at the ISIS Pulsed Neutron and Muon Source.^28^ Samples of roughly 800 mg of powder were placed in aluminum holders fitted with a titanium window. Titanium foils were fitted to the sample holder to attenuate the muon momentum and optimize the signal obtained. The sample holder was mounted on a closed cycle refrigerator which maintained the temperature, as detailed in the spectra. Data were processed, including background subtraction and peak fitting, using Mantid.^29^ Backgrounds were fitted using a multipoint polynomial which was constructed based on background data collected as part of our previous experimental runs.^11^ The background data were obtained by filling the sample cell with aluminum sheets to give an equivalent areal density compared to the sample to ensure the correct stopping position of the muonium.

## Simulations

Crystal structures for complexes **4**, **5**, and **6** were obtained from the Cambridge Structural Database; the structure for complex **6** was modified to simplify the disordered cation. Implantation sites for the muonium were selected by hand based on known reactivity and the muonium placed using a custom Python script. Implantation was explored for the metal-bridging site, at the lone pair of each unique sulfur, at each unique triple-bonded carbon, and at each terminal oxygen and nitrogen atom.

The DFT computer simulations carried out in this work were performed with the CASTEP.^17^ A plane wave cutoff of 850 eV and a low-density 1 × 1 × 1 Monkhorst–Pack *k*-point grid^30^ were used. The Meta-GGA RSCAN^31^ exchange–correlation functional was used in combination with autogenerated ultrasoft pseudopotentials, and the DFT calculations were spin-polarized. A specific mass of 0.113, 428, 925, and 9 AMU and magnetogyric ratio of 851, 615, 456.597, and 8916 rad s^–1^ T^–1^ were defined for the muonium. Geometry relaxations were carried out until the forces were converged within a 0.05 eV per atom threshold. Then, hyperfine calculations were carried out on the relaxed structures. The purpose was to calculate the hyperfine coupling tensors for the muonium at the Fe−μ–Fe muonide, which were then used as input for the simulation of ALC-μSR experiments using MuSpinSim software as implemented in the Galaxy platform.^32,33^

## Supporting Information Available

ALC-μSR spectrum for **6**, plot of temperature dependence of peak maximum for **4**, relative energies, hyperfine tensors and final atomic coordinates for all muoniated radicals examined by DFT, and extended details for the use of MuSimSpin to simulate ALC-μSR spectra (PDF)
