# Supplementary material for: Hydrogen Radical Chemistry at High-Symmetry {2Fe2S} Centers Probed Using a Muonium Surrogate
Source: Inorg Chem. 2025 Mar 1;64(10):5053–8. doi: 10.1021/acs.inorgchem.4c05126 (PMC11920949; doi:10.1021/acs.inorgchem.4c05126)
Supplement: Supplementary file 1 — ic4c05126_si_001.pdf [file ic4c05126_si_001.pdf]

Supporting Information for: Hydrogen radical chemistry at high-symmetry {2Fe2S} centers probed using a muonium surrogate

Joseph A. Wright[a,*] Farhana Haque[a] Leandro Liborio[b]
Stephen P. Cottrell[c]

[*]Email: joseph.wright@uea.ac.uk
[a]Energy Materials Laboratory, School of Chemistry, Pharmacy and Pharmacology, University of East Anglia, Norwich Research Park, Norwich, NR4 7TJ, United Kingdom
[b]Scientific Computing Department, Science & Technology Facilities Council, Rutherford Appleton Laboratory, Harwell Science Campus, Oxfordshire, OX11 0QX, United Kingdom
[c]ISIS Facility, Science & Technology Facilities Council, Rutherford Appleton Laboratory, Harwell Science Campus, Oxfordshire, OX11 OQX, United Kingdom

1 ALC-μSR Analysis

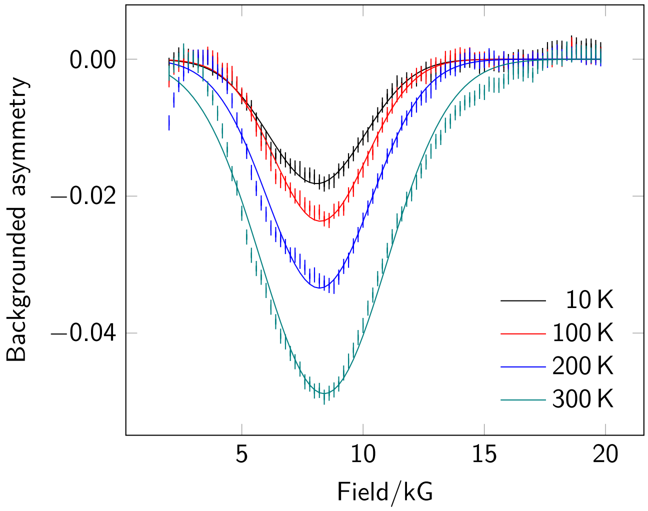

Figure 1: Background-subtracted ALC-μSR spectra for **6**. Data points are shown as sticks representing the estimated uncertainty in each point. Gaussian fits are shown as superimposed lines.

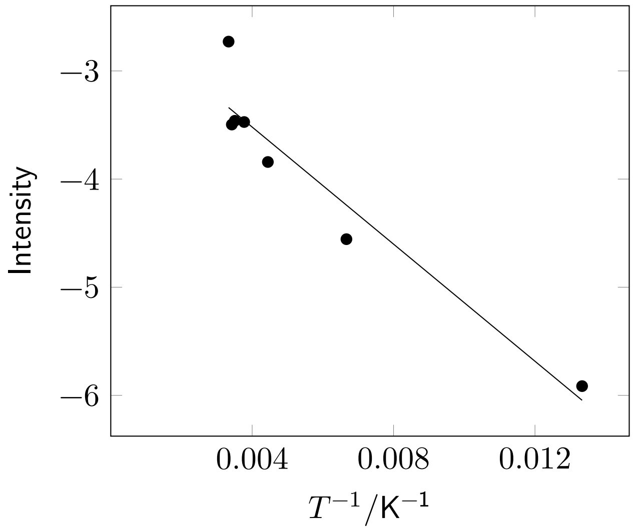

Figure 2: Peak intensity versus reciprocal temperature for the ALC-μSR spectra for **4**, showing least-squares regression fit line

2 Relative energies of implantation sites

Energies for the implantation sites are taken from the CASTEP output and are given relative to the lowest energy position for the parent molecule.

2.1 Complex 4

Site	Energy/kJ mol^{-1}
Fe−μ−Fe	0.0
C_{basal}	77.3
O_{basal}	174.2
C_{apical}	66.5
O_{apical}	150.8
S	99.3

2.2 Complex 5

Site	Energy/kJ mol^{-1}
Fe−μ−Fe	0.0
C_{basal} geminal to P	68.9
C_{basal} *trans* to P	65.2
C_{basal} *cis* to P	65.8
C_{apical}	63.0
O_{apical}	174.0
S *trans* to P	84.7
S *cis* to P	92.9

2.3 Complex 6

Site	Energy/kJ mol^{-1}
Fe−μ−Fe	0.0
C_{basal} geminal to CN	71.2
$\mathit{C}{\equiv}O_{basal}$ *trans* to CN	56.5
$\mathit{C}{\equiv}O_{basal}$ *cis* to CN	43.1
$\mathit{C}{\equiv}O_{apical}$	50.2
$\mathit{C}{\equiv}N_{basal}$	69.2
$C{\equiv}\mathit{N}_{basal}$	77.1
$\mathit{C}{\equiv}N_{apical}$	80.5
$C{\equiv}\mathit{N}_{apical}$	88.1
S *trans* to CN	101.0
S *cis* to CN	94.4

3 Calculated hyperfine tensors

3.1 Complex 4

3.1.1 Fe−μ−Fe site

331.9337	4.1348	57.6038
4.1348	281.3148	−31.3247
57.6038	−31.3247	222.7654

3.1.2 C_{basal}

−994.0951	−22.4061	−4.2336
−22.4061	−1035.9237	18.3778
−4.2336	18.3778	−1036.6223

3.1.3 O_{basal}

−256.3756	−24.5721	−25.5850
−24.5721	−293.8480	−46.7653
−25.5850	−46.7653	−275.3612

3.1.4 C_{apical}

−333.6498	6.1069	11.1295
6.1069	−341.3548	−16.6386
11.1295	−16.6386	−361.9486

3.1.5 O_{apical}

−188.8295	−6.5264	11.2652
−6.5264	−234.4778	43.2931
11.2652	43.2931	−226.4598

3.1.6 S

−198.2384	−2.0924	−3.3515
−2.0924	−200.7383	−4.0826
−3.3515	−4.0826	−194.2104

3.2 Complex 5

3.2.1 Fe−μ−Fe site

192.7390	41.7067	9.9746
41.7067	267.5638	21.0320
9.9746	21.0320	340.6505

3.2.2 C_{basal} geminal to P

−734.8457	−19.4994	4.3047
−19.4994	−752.2045	−4.1489
4.3047	−4.1489	−780.3862

3.2.3 C_{basal} *trans* to P

−617.6717	−13.5846	21.8254
−13.5846	−639.0391	−3.6911
21.8254	−3.6911	−671.5725

3.2.4 C_{basal} *cis* to P

−639.2139	0.1130	−35.3672
0.1130	−648.0040	6.0048
−35.3672	6.0048	−635.3286

3.2.5 C_{apical}

−763.5314	5.9306	−9.5611
5.9306	−735.1393	2.2379
−9.5611	2.2379	−777.3354

3.2.6 O_{apical}

−227.2263	3.0480	26.0975
3.0480	−150.1617	−5.7622
26.0975	−5.7622	−161.5300

3.2.7 S *trans* to P

−127.0684	2.0101	0.2573
2.0101	−132.0776	−2.7580
0.2573	−2.7580	−139.0992

3.2.8 S *cis* to P

−101.0771	4.8048	−2.1154
4.8048	−109.0404	3.2076
−2.1154	3.2076	−107.3056

3.3 Complex 6

3.3.1 Fe−μ−Fe site

293.1785	−10.9039	−2.4734
−10.9039	352.4913	−36.3386
−2.4734	−36.3386	200.4175

3.3.2 $C \equiv O_{basal}$ geminal to CN

−803.7702	9.4272	5.0640
9.4272	−812.4152	10.9225
5.0640	10.9225	−757.9011

3.3.3 $C \equiv O_{basal}$ *trans* to CN

−524.2043	−27.8340	9.7831
−27.8340	−564.8617	−16.5354
9.7831	−16.5354	−521.1277

3.3.4 $C \equiv O_{basal}$ *cis* to CN

−358.3843	27.7110	−0.3344
27.7110	−333.5140	1.2109
−0.3344	1.2109	−328.4403

3.3.5 $C \equiv O_{apical}$

−572.3311	7.3403	−14.3137
7.3403	−586.4501	−3.1868
−14.3137	−3.1868	−571.5342

3.3.6 $C \equiv N_{basal}$

−824.7265	8.8967	−9.0100
8.8967	−819.5503	9.2778
−9.0100	9.2778	−816.5987

3.3.7 $\mathrm{C} \equiv N_{basal}$

−47.9946	−1.2960	1.4638
−1.2960	−50.7562	2.3614
1.4638	2.3614	−49.2435

3.3.8 $C \equiv N_{apical}$

−953.4716	11.6628	−3.6386
11.6628	−932.0900	1.4321
−3.6386	1.4321	−920.4123

3.3.9 $\mathrm{C} \equiv N_{apical}$

−454.8889	−1.2101	−3.5018
−1.2101	−456.9830	−5.0031
−3.5018	−5.0031	−464.2036

3.3.10 S *cis* to CN

−178.8708	1.7695	−0.1523
1.7695	−173.1417	0.0844
−0.1523	0.0844	−160.9540

3.3.11 S *trans* to CN

−188.7384	−2.2060	1.0915
−2.2060	−191.5274	2.7238
1.0915	2.7238	−178.7677

4 Simulating ALC-μSR spectra experiments with `MuSpinSim` and Galaxy

Section (3) has all the CASTEP-calculated hyperfine tensors for muonium added in different positions of the complexes studied in this work. This is the only DFT input needed by `MuSpinSim` to simulate the corresponding ALC-μSR spectra experiment.

`MuSpinSim` has been released in the Python Package Index PyPI: `https://pypi.org/project/muspinsim/`, can be downloaded and installed in Linux or Mac using pip using the following command: `pip install muspinsim`, and in Windows by using `conda install muspinsim`.

Once installed, `MuSpinSim` can be run from the command line by typing: `muspinsim input-file.in`. A representative input-file for an ALC-μSR spectra simulation of the Fe−μ−Fe site in complex **5** is:

```
 name
    HydrogenaseRB
spins
    mu e
hyperfine 1
    192.7390 41.7067 9.9746
    41.7067 267.5638 21.0320
    9.9746 21.0320 340.6505
average_axes
    orientation
field
    range(0.2,2.0,200)
orientation zyz
    zcw(20)
experiment
    alc
```

The meaning and options for all the potential variables in MuSpinSim can be found here: https://muon-spectroscopy-computational-project.github.io/muspinsim/input/, together with detailed examples of the sort of experiments that can be simulated with MuSpinSim.

In Galaxy, MuSpinSim is in the Galaxy instance https://materialsgalaxy.stfc.ac.uk/, which is maintained by the Scientific Computing Department at STFC, and it contains the so called"Galaxy tools" that we developed for different areas of materials science. Figure 3 shows the main Galaxy interface, which the user encounters when going to https://materialsgalaxy.stfc.ac.uk/. On the left panel, under **MUONS** there is a list of all the available Galaxy tools for simulating muon experiments. When **MuSpinSim** is chosen, there is a new unfolded menu with several options. When the **MuSpinSim Configure** tool is chosen, the central panel of Galaxy UI shows a menu with all the potential variables available for MuSpinSim, and we use this to set up the MuSpinSim ALC-μSR spectra simulation input file. Then we move to the **MuSpinSim Simulate** tool, which runs the simulation. And, finally, we move to the **MuSpinSim Plot** to plot the ALC-μSR spectra and visualize it in Galaxy. We can also obtain a file that can be plotted in an external software.[1]

All the calculations run for the ALC-μSR spectra experiment are saved in Galaxy, and can be visualized on the right panel of the Galaxy UI.

[1]The plots in the main text and this supporting file were prepared using the exported simulation files in the LaTeX package pgfplots.

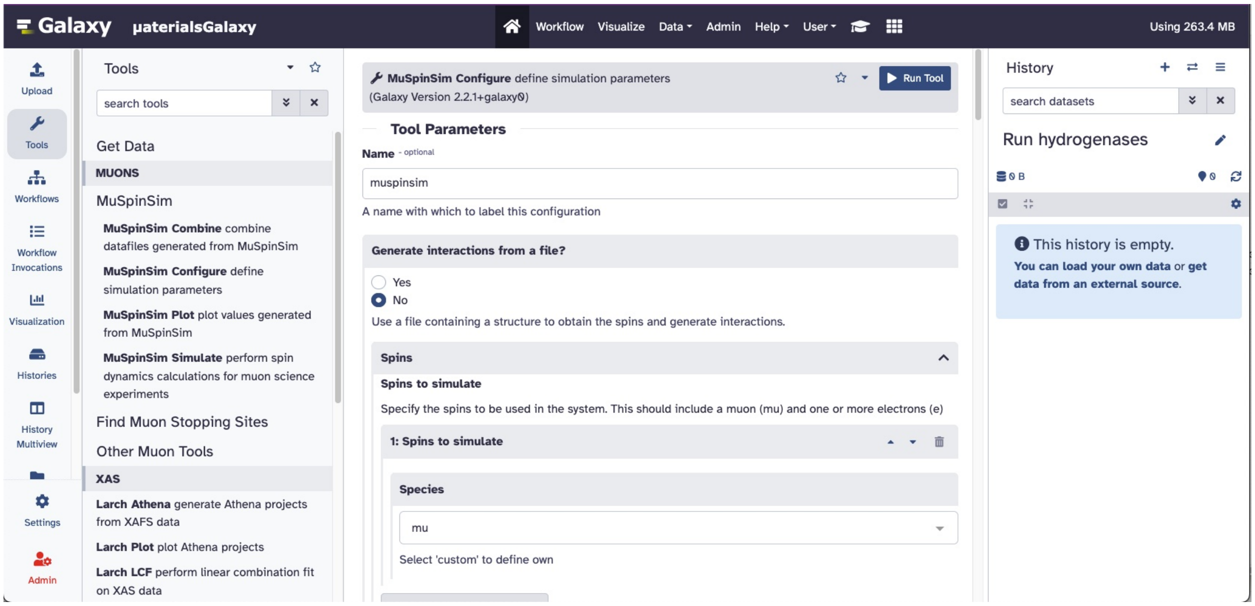

Figure 3: Main Galaxy UI. The left panel shows a list of the Galaxy tools available. The image shows what appears in the central panel once the **MuSpinSim Configure** tool is chosen. There is a list of the variables available to **MuSpinSim**, and we can choose what to simulate and build the corresponding **MuSpinSim**'s input file. The **MuSpinSim Simulate** tool then runs the simulation. And the **MuSpinSim Plot** tool plots the ALC-μSR spectra graph and visualize it in Galaxy.

5 Final atomic coordinates

5.1 Complex 4

5.1.1 Fe−μ−Fe site

```
89
Converted from jawg013a-out.cell by c2x
H -0.46695326 5.71433893 8.01848751
H 5.03495146 2.13144733 3.28454736
H 1.98030603 13.71312740 6.24444804
H 7.50669158 10.03356889 1.49604341
H 0.20981391 5.20526719 6.47992980
H 5.75022086 2.64248741 1.76388707
H 1.28429699 13.18575914 7.77011694
H 6.81138853 10.52250311 3.03488038
H 7.19780849 5.44569558 5.44029087
H 3.69539612 2.38509832 0.68673180
H 3.34523787 13.45318770 8.82697027
H -0.16764218 10.29591796 4.09168376
H 6.53838000 6.01220056 6.97162495
H 3.00825377 1.79148670 2.19885094
H 4.02517774 14.04507117 7.31075173
H 0.51299295 9.75912292 2.55602263
C 0.16909090 1.84154614 0.45921074
C 3.76115384 6.12675666 4.96642125
C 3.26643110 9.71782255 4.53915017
C 6.78736623 14.09809068 9.27383093
C 8.27536080 4.31060499 0.17219623
C 2.73438973 3.65015183 4.93012567
C 4.29338843 12.19374321 4.57191996
C -1.22873739 11.66194951 9.34172660
C 4.95165033 2.18991187 8.77466384
C 1.48665923 5.82292572 3.87811458
C 5.54166377 10.02073662 5.62542626
C 1.96893098 13.78326245 0.94249043
C -0.35618910 0.77664060 6.56887843
C 5.02027730 7.13258004 2.08427960
C 2.00660083 8.70662695 7.41885709
C 7.48791060 15.01953921 2.69087709
C -0.81079642 2.66799742 4.72894151
C 2.69811786 5.42000043 9.51749666
C 4.33222920 10.41636711 -0.01483906
C 7.86651323 13.30903141 4.74535391
C 6.30677139 0.97405133 5.59151218
```

C 2.67959987 6.85506044 1.13720401
C 4.35913345 8.98344098 8.34693233
C 0.78457621 14.82487910 3.65364718
C -0.60571706 5.03503149 7.18028917
C 4.91270523 2.80184002 2.43704573
C 2.11579576 13.03629802 7.08523498
C 7.63654871 10.69697449 2.34860566
C 7.09333111 5.20415395 6.49698968
C 3.58003658 2.60031077 1.74735056
C 3.45408282 13.23754746 7.76517320
C -0.07048828 10.54259007 3.03601622
O 0.65248573 1.14419546 1.22853950
O 4.24132526 6.86968560 5.69414593
O 2.78399515 8.97533051 3.81230674
O 6.31773873 14.84060085 8.53672153
O 8.05220158 5.19378555 0.86118997
O 2.54045954 2.77369065 5.64067227
O 4.48297566 13.06798575 3.85776596
O -1.02542386 10.78134337 8.63951205
O 3.90497783 1.72953821 8.82053178
O 0.48821467 6.38186157 3.91966527
O 6.53886660 9.46008374 5.58534878
O 2.97206110 14.33344425 0.91543489
O 0.33779385 15.78535498 6.86565645
O 5.68978461 8.01299795 2.38426277
O 1.33285743 7.82987862 7.11907938
O 6.87539389 0.04671739 2.28733323
O -0.42115737 2.92473266 3.68395300
O 3.10366300 5.15357363 8.47854833
O 3.92334968 10.69034730 1.02108867
O 7.45484015 13.07389982 5.78950215
O 5.47662997 0.29105287 5.19669776
O 1.83601453 7.56190528 0.82014445
O 5.21332119 8.28160187 8.64450176
O 1.62433352 15.54679603 3.94375357
S -0.38354695 3.35602344 7.83509635
S 5.09679746 4.49204053 3.09000820
S 1.93405202 11.34973468 6.42115269
S 7.42692750 12.39121756 1.71345806
S 6.02620337 3.73237809 6.58096904
S 2.52560462 4.08705788 1.85260282
S 4.50775395 11.75085449 7.65369019
S 0.95538740 12.04980418 2.95352913
Fe 6.56592242 2.86878230 8.63795583
Fe 3.04559013 4.99261222 3.82984040

```
Fe 3.98377300 10.85230838 5.67452382
Fe 0.41182780 12.95543306 0.98292686
Fe 7.54429044 2.03011988 6.26017055
Fe 3.98282056 5.77678567 1.63649070
Fe 3.04826509 10.06086744 7.86595560
Fe -0.51391725 13.72625700 3.18417304
H 6.82937005 1.54001070 7.68904691
```

5.1.2 C_{basal}

```
89
Converted from jawg013b-out.cell by c2x
H -0.41829740 5.76202574 7.80051077
H 5.10878446 2.13286387 3.17075633
H 2.05865196 13.74556502 6.02600957
H 7.54992306 10.17429626 1.42079388
H 0.26945135 5.27630592 6.25787270
H 5.77383398 2.71127302 1.65098962
H 1.37530872 13.25182736 7.56843232
H 6.85987344 10.66453362 2.96124882
H 7.24434239 5.51810173 5.26663643
H 3.71241802 2.39543951 0.62219892
H 3.45111818 13.54170940 8.59548611
H -0.11602453 10.39902803 3.95468092
H 6.57476323 6.07875049 6.79733430
H 3.04174602 1.80888743 2.14382148
H 4.11340483 14.09612864 7.05752731
H 0.55687521 9.85141584 2.42072728
C 0.25110837 1.76285987 0.23455713
C 3.91763830 6.16961234 4.97611245
C 3.31833584 9.78278298 4.33792957
C 6.90260606 14.15874726 8.99623401
C 8.26482381 4.24318121 0.24551380
C 2.65718188 3.47650451 4.77069686
C 4.36374201 12.25323664 4.40865373
C -1.11578534 11.67852770 8.99501796
C 5.13441001 2.02323371 8.41309397
C 1.55543329 5.86479482 3.86462684
C 5.60986110 10.05310779 5.43773548
C 2.01742112 13.90195293 0.80987420
C -0.40540164 0.77183059 6.62162827
C 5.00095692 7.18003666 2.05567281
C 2.14947272 8.73938017 7.22079106
C 7.55442734 15.15119988 2.60932459
C -0.79092821 2.49960923 4.57008593
```

C 2.76569644 5.38331243 9.31160628
C 4.37001736 10.54037337 0.01568373
C 7.93320759 13.41879691 4.65936711
C 6.29613259 0.99669610 5.72121223
C 2.67925937 6.89143307 1.12515432
C 4.45995125 9.02816365 8.15485336
C 0.85285224 14.92036430 3.51083093
C -0.55108961 5.09860483 6.94855751
C 4.95007298 2.82964244 2.34977958
C 2.20124550 13.08658952 6.88058134
C 7.68561490 10.83619201 2.27386821
C 7.15238549 5.28617819 6.32609690
C 3.60459717 2.61793424 1.68231419
C 3.54728252 13.30058991 7.53811908
C -0.02072108 10.64004786 2.89784541
O 0.72458042 1.00702393 0.95321088
O 4.49250593 7.19384479 5.13815987
O 2.80578006 9.06653977 3.60728579
O 6.42749370 14.91146387 8.27420476
O 8.07067832 5.13212221 0.94003537
O 2.40674344 2.51417850 5.33287969
O 4.54911245 13.16028474 3.73540241
O -0.92817217 10.78652226 8.30253042
O 4.13691553 1.46146014 8.40647182
O 0.55702002 6.42823676 3.83053616
O 6.61005046 9.49830748 5.41373726
O 3.01030606 14.47110557 0.78949415
O 0.26670590 15.86663488 6.90217098
O 5.65391868 8.08615107 2.30007492
O 1.52040690 7.83432058 6.90365084
O 6.88166378 0.05538095 2.32552335
O -0.37879044 2.75784789 3.53209386
O 3.18273639 5.11435044 8.27637265
O 3.95248627 10.80308082 1.05079051
O 7.52203320 13.15931156 5.69763468
O 5.45586576 0.27386140 5.43444698
O 1.85703391 7.61362503 0.78921675
O 5.29639124 8.29993613 8.44273956
O 1.69303143 15.64451585 3.79611192
S -0.33715274 3.40465490 7.58572061
S 5.11811354 4.50505790 3.05464509
S 2.01344489 11.39089063 6.24511779
S 7.48303085 12.53016781 1.63791543
S 6.12603281 3.77858043 6.43033021
S 2.54087659 4.09904475 1.79981930

```
S 4.59782999 11.81002369 7.44586291
S 1.01603338 12.14072754 2.80773067
Fe 6.68221217 2.86972374 8.39255644
Fe 3.07362084 4.95683622 3.83470503
Fe 4.05580923 10.89525478 5.48415695
Fe 0.46929114 13.05593622 0.84243292
Fe 7.59257831 2.09984684 6.18948787
Fe 3.97764179 5.80324480 1.63181317
Fe 3.15206579 10.11166985 7.68523224
Fe -0.44718367 13.82304503 3.04269896
H 3.78809322 5.44321185 5.84385258
```

5.1.3 O_{basal}

```
89
Converted from jawg013c-out.cell by c2x
H -0.35307012 5.66438797 7.79878328
H 5.12592616 2.13946235 3.26557884
H 2.08589520 13.48657304 5.99021482
H 7.62391854 9.90237843 1.41342903
H 0.31540292 5.13123461 6.26349571
H 5.83357634 2.68015601 1.74766742
H 1.40787550 12.96855364 7.52444908
H 6.94365013 10.42205555 2.94517329
H 7.38684570 5.42227032 5.31893629
H 3.81222438 2.28845388 0.66520458
H 3.48095632 13.27550353 8.55533350
H -0.14156691 10.17341562 3.90593036
H 6.73802872 5.95745104 6.86665116
H 3.09300498 1.76974766 2.18999100
H 4.15109438 13.82761535 7.02350532
H 0.53030781 9.62054379 2.37350833
C 0.23875017 1.75458919 0.24167172
C 3.60916326 6.26223101 4.83629371
C 3.37691018 9.58649788 4.22469309
C 6.99137488 13.80394569 8.96520459
C 8.35652825 4.22013595 0.22031378
C 2.78712767 3.68327862 4.82123880
C 4.40990246 12.01601980 4.37351788
C -1.12308677 11.36831608 8.98886504
C 5.22791846 1.93116861 8.46329901
C 1.42423785 5.75159826 3.69906641
C 5.68225335 9.83810623 5.39987663
C 2.00588712 13.65901358 0.75298679
C -0.39379342 0.69761934 6.65102869
```

C 5.09959643 7.07697934 1.98684241
C 2.09735745 8.50981063 7.20942947
C 7.61671604 14.89222693 2.55140784
C -0.78497799 2.42892457 4.59770378
C 2.85022226 5.23436654 9.24005742
C 4.39177924 10.35531706 -0.08725598
C 8.00144891 13.17205483 4.61473020
C 6.40091081 0.90973308 5.76959626
C 2.74056305 6.80358284 1.03799113
C 4.48558655 8.78462531 8.16908786
C 0.82021905 14.68721819 3.43975413
C -0.50856033 4.98897573 6.95972158
C 4.98606425 2.80855180 2.41771283
C 2.23613321 12.81819901 6.83591580
C 7.76753814 10.57874660 2.25323678
C 7.30419033 5.17029128 6.37477754
C 3.66738565 2.55990832 1.71022511
C 3.58012569 13.03229947 7.49880610
C -0.04610605 10.41227408 2.84805773
O 0.72149433 1.00964661 0.96777733
O 4.60254848 6.56580928 5.59194138
O 2.86397696 8.92895059 3.44270439
O 6.49284400 14.51522579 8.21925918
O 8.15426369 5.13732448 0.87406405
O 2.64351149 2.79275338 5.52744816
O 4.58310606 12.92106211 3.69743604
O -0.93590983 10.46201833 8.31464602
O 4.22686500 1.37441403 8.46973126
O 0.39421385 6.24994737 3.70618480
O 6.69685322 9.30649439 5.40000224
O 3.00869058 14.21149589 0.74554090
O 0.24709583 15.56846747 6.84241977
O 5.78876366 7.93761561 2.30876294
O 1.38100445 7.66528089 6.92458730
O 6.97724000 0.01854760 2.35524960
O -0.38884440 2.70399896 3.55766966
O 3.24832026 4.98133718 8.19257603
O 3.98396927 10.64928268 0.94034828
O 7.59558652 12.89924536 5.65249170
O 5.54514484 0.20625608 5.47688060
O 1.88166097 7.49828109 0.72665363
O 5.34390340 8.09962913 8.50035348
O 1.67743009 15.38909428 3.72665350
S -0.32957856 3.30377447 7.62309454
S 5.11466867 4.50795337 3.07257797

S 2.06069603 11.12689198 6.18092817
S 7.57095291 12.26518662 1.59417722
S 6.25727875 3.67772438 6.46348242
S 2.59779460 4.03570618 1.72415061
S 4.62958357 11.54110301 7.41235710
S 0.98319523 11.91777897 2.75822787
Fe 6.78640395 2.76562392 8.43742177
Fe 3.03980221 5.01525537 3.71253968
Fe 4.11026112 10.63880538 5.43319500
Fe 0.45286942 12.81400476 0.78018526
Fe 7.71078775 1.99204355 6.24182596
Fe 4.05457788 5.74223571 1.52801370
Fe 3.18244360 9.83803800 7.62972138
Fe -0.48579839 13.59507949 2.97119339
H 4.47629121 7.44327369 6.00392721

5.1.4 C_{apical}

89
Converted from jawg013d-out.cell by c2x
H -0.42663657 5.76675791 7.88307756
H 5.07674637 2.16498144 3.23759940
H 2.11199149 13.69815788 6.05611310
H 7.59002116 10.12792001 1.47612304
H 0.26659256 5.27584301 6.34478765
H 5.76531864 2.65936744 1.69696317
H 1.35957395 13.26602681 7.59184184
H 6.88892828 10.58388173 3.02094546
H 7.23291195 5.51898237 5.34008044
H 3.69241038 2.37377175 0.66314484
H 3.40777158 13.50162844 8.68329541
H -0.08331677 10.38502064 4.02860095
H 6.56085129 6.08176469 6.86830315
H 3.02408733 1.81821223 2.19774977
H 4.10078742 14.07060944 7.15923645
H 0.59782952 9.82571085 2.50191329
C 0.24125069 1.75384669 0.24073532
C 3.80013509 6.15716717 4.91846216
C 3.34199939 9.77542930 4.41338814
C 6.86944564 14.15638395 9.13225589
C 8.24895247 4.22344827 0.21789799
C 2.77812330 3.67161632 4.85616748
C 4.44680192 12.13410523 4.18654289
C -1.12914930 11.68614013 9.14850945
C 5.10678025 2.03030604 8.50718614

C 1.53170823 5.86258226 3.82105361
C 5.60285871 10.08831161 5.44716791
C 2.00434226 13.88127857 0.85998390
C -0.41781566 0.78675321 6.69251655
C 5.01140510 7.16536956 2.01810418
C 2.09725446 8.73273926 7.41163121
C 7.54023370 15.11794228 2.67634515
C -0.80079834 2.51274344 4.64232075
C 2.76994171 5.39144346 9.31634758
C 4.35144868 10.51817274 0.07276073
C 7.92199688 13.39682622 4.73845419
C 6.27691311 1.01248774 5.77508046
C 2.69630977 6.87813730 1.08690380
C 4.43407863 9.02745927 8.32922153
C 0.84284477 14.89722891 3.59400047
C -0.55907605 5.10275013 7.03132563
C 4.93804236 2.82487987 2.38358806
C 2.20150081 13.06146950 6.93398128
C 7.70871543 10.78424432 2.33556420
C 7.13694374 5.28787140 6.39898448
C 3.59326637 2.61380515 1.72042928
C 3.53276913 13.26989346 7.62731915
C 0.01343417 10.61461711 2.96940608
O 0.71466416 1.01274093 0.97586966
O 4.27131428 6.89438533 5.65832807
O 2.85531116 9.01051272 3.70638406
O 6.39748164 14.90269284 8.40259315
O 8.05922923 5.11928136 0.90458890
O 2.58810935 2.77286746 5.53852906
O 4.26138775 13.26880746 4.53131103
O -0.93394775 10.79186694 8.46183771
O 4.10936580 1.47007431 8.54267788
O 0.53371717 6.42201370 3.86254745
O 6.58561284 9.49400648 5.41147247
O 3.00383538 14.43908185 0.82993418
O 0.25132981 15.84893468 7.02271699
O 5.64588848 8.06976149 2.32253752
O 1.45381858 7.83064134 7.12676071
O 6.87629079 0.05713685 2.33815727
O -0.39246072 2.76590730 3.60109999
O 3.17716065 5.13019703 8.27689822
O 3.93703549 10.78083602 1.10993150
O 7.51221422 13.14891485 5.77956449
O 5.44070742 0.29070498 5.47561083
O 1.86019661 7.60304647 0.79141633

```
O 5.27004532 8.30819633 8.63459080
O 1.67869640 15.62380636 3.88209451
S -0.34897962 3.40913967 7.66720351
S 5.12920724 4.52274548 3.01776643
S 1.98673194 11.35909352 6.32695462
S 7.47222495 12.48129491 1.71491735
S 6.10366931 3.78703791 6.49561641
S 2.54570141 4.10604571 1.80919195
S 4.60366185 11.79585502 7.54347612
S 1.02862758 12.12688156 2.87220199
Fe 6.65781274 2.87301444 8.46040409
Fe 3.08669780 5.02808926 3.77324991
Fe 4.05607912 10.89496222 5.53572804
Fe 0.45752491 13.03527122 0.90947550
Fe 7.57255809 2.11142061 6.25502312
Fe 3.99958464 5.79470944 1.56684738
Fe 3.12635620 10.10465202 7.82468218
Fe -0.45186671 13.79271887 3.12072062
H 4.79448771 11.88692593 3.16943651
```

5.1.5 O_{apical}

```
89
Converted from jawg013e-out.cell by c2x
H -0.46977630 5.73206930 7.95537960
H 5.10403216 2.12960444 3.11909143
H 2.00001614 13.57972867 6.14151153
H 7.54466995 9.98155643 1.39763433
H 0.21013877 5.19422295 6.42371134
H 5.79682993 2.63975792 1.58062519
H 1.32440903 13.04224261 7.67472662
H 6.87032184 10.50106978 2.93587191
H 7.20604616 5.52956798 5.31559966
H 3.72191356 2.37755623 0.55108024
H 3.38989526 13.35727596 8.70298438
H -0.11390703 10.18880115 4.02447006
H 6.53429277 6.10270920 6.84526249
H 3.06854617 1.79308484 2.08274802
H 4.05862282 13.92094450 7.17114377
H 0.55112044 9.63266002 2.48719248
C 0.21441649 1.75540684 0.24328872
C 3.77464096 6.00612573 4.92371216
C 3.32635621 9.62337884 4.41111462
C 6.82050768 13.98051759 9.10647482
C 8.26202294 4.27225825 0.11254331
```

C 2.86325551 3.51645764 4.82596181
C 4.33731389 12.10142520 4.49160619
C -1.20400526 11.51118627 9.22462447
C 4.97845059 2.10412388 8.41925717
C 1.51779850 5.61066382 3.88554803
C 5.60650298 9.92883232 5.53590077
C 2.04619684 13.68599414 0.87296015
C -0.54409646 0.76349235 6.77868830
C 5.04695640 7.12795036 1.99748975
C 2.04702647 8.59278707 7.32179235
C 7.58122524 14.95592010 2.56920274
C -0.88979613 2.48735876 4.73838427
C 2.67029123 5.37314678 9.37048608
C 4.42354190 10.34565201 -0.08426775
C 7.93201158 13.23833442 4.62313743
C 6.14025133 1.07315192 5.74515394
C 2.69980880 6.88776440 1.08052530
C 4.42941473 8.88012029 8.25215514
C 0.85422179 14.70004702 3.62925541
C -0.61996227 5.06133260 7.11221513
C 4.97374819 2.80303826 2.27356975
C 2.15079588 12.90486248 6.98082936
C 7.69318780 10.65294431 2.24093759
C 7.10009912 5.30133231 6.37435628
C 3.62583959 2.60071704 1.61213387
C 3.49310587 13.12195890 7.64544895
C -0.01814704 10.42655889 2.96679381
O 0.70174607 1.01423849 0.96843756
O 4.22339294 6.73396195 5.68752724
O 2.83709578 8.90542786 3.66546819
O 6.35181673 14.70948492 8.35623276
O 8.08113724 5.17468904 0.79075247
O 2.18854367 3.16501971 5.85629701
O 4.52164133 12.99456050 3.79882768
O -1.00558744 10.64233054 8.50794854
O 3.95313123 1.58975674 8.35145192
O 0.48263890 6.09806064 3.97214135
O 6.61975041 9.39562390 5.52132563
O 3.04857600 14.23754978 0.83202444
O 0.20163263 15.65519269 7.09691855
O 5.74105145 7.98888898 2.30481749
O 1.34081450 7.74052874 7.03019715
O 6.86587238 0.07990159 2.23508494
O -0.45207948 2.73360531 3.70903179
O 3.09614212 5.08749593 8.34355877

```
O 3.99597623 10.62494096 0.94241555
O 7.50497035 12.99904271 5.65988288
O 5.25718454 0.40926144 5.44032389
O 1.86893587 7.61791798 0.77603461
O 5.30210789 8.20902760 8.56593507
O 1.67617596 15.42797357 3.96093557
S -0.46245331 3.37197935 7.76882541
S 5.17491696 4.48529410 2.94113295
S 1.98431280 11.21823955 6.31320480
S 7.50668491 12.34469985 1.59406521
S 6.03819451 3.82048445 6.46552149
S 2.56404214 4.07894045 1.73976382
S 4.54973043 11.63795633 7.55068248
S 1.02553629 11.92294538 2.88200972
Fe 6.54761072 2.89913811 8.43706570
Fe 3.09772563 4.87674780 3.76614542
Fe 4.03713779 10.74162446 5.56982083
Fe 0.48933273 12.85208908 0.91880152
Fe 7.47132846 2.11475025 6.23953737
Fe 4.00540643 5.79198341 1.53221239
Fe 3.10385278 9.93617362 7.75994048
Fe -0.43135594 13.61005483 3.12253553
H 2.40837282 2.27131436 6.16672704
```

5.1.6 S

```
89
Converted from jawg013f-out.cell by c2x
H -0.42081025 5.80757416 7.97774562
H 4.95332289 1.91357911 3.19783081
H 2.02628792 13.82254907 6.20343628
H 7.54824185 10.20847324 1.52657962
H 0.27532121 5.30569288 6.44156261
H 5.72420551 2.48349648 1.61874444
H 1.35125348 13.30168357 7.74124590
H 6.87242423 10.70607587 3.07144897
H 7.32856195 5.54001065 5.42655556
H 3.49737279 2.39943879 0.54731105
H 3.42148971 13.59358794 8.76897631
H -0.14737356 10.46954873 4.06472201
H 6.63241825 6.11010663 6.94453329
H 2.77495315 1.82390833 2.06408200
H 4.08329844 14.17249198 7.24173638
H 0.50589672 9.89060680 2.53183873
C 0.22337410 1.75890545 0.25446487
```

C 3.69674575 6.25395520 5.06969224
C 3.30953403 9.81842427 4.51098831
C 6.88407926 14.22224017 9.20069315
C 8.28617528 4.21517021 0.23927276
C 2.83473472 3.72033487 4.89546404
C 4.34305773 12.30765903 4.55094638
C -1.17317013 11.78095624 9.23204526
C 5.14257759 2.04976481 8.52938754
C 1.49624636 5.83582651 3.83515741
C 5.58845217 10.10673609 5.60075882
C 1.97681926 13.95245615 0.89600396
C -0.43401008 0.81633445 6.76832924
C 4.92556533 7.41056139 1.95739429
C 2.10696089 8.81436070 7.41437142
C 7.61630596 15.18348047 2.72212735
C -0.80813376 2.55507190 4.74352617
C 2.77013609 5.51800097 9.46423244
C 4.36317475 10.56923705 0.01627467
C 7.96123870 13.44577838 4.74516712
C 6.32080176 1.02580611 5.83833329
C 2.60379680 6.89972616 1.12120694
C 4.43380457 9.09809082 8.33854350
C 0.84022865 14.96725669 3.64412401
C -0.55272562 5.14149865 7.12730048
C 4.83535893 2.28749144 2.19403479
C 2.17602495 13.15204611 7.04801430
C 7.69448991 10.86902954 2.37873791
C 7.20622178 5.30856665 6.48260785
C 3.51943401 2.48907244 1.63302714
C 3.52157463 13.36637418 7.70867423
C -0.05499381 10.69494209 3.00345660
O 0.70171558 1.00562581 0.97362518
O 4.13905966 6.96183709 5.85509097
O 2.81401064 9.06882883 3.79925273
O 6.40379429 14.95959005 8.46563577
O 8.09272991 5.11757467 0.91628691
O 2.62420514 2.79419002 5.53304948
O 4.52349422 13.20655634 3.86674801
O -0.97805963 10.88215288 8.54966932
O 4.14810318 1.48596091 8.49340335
O 0.45319936 6.31018903 3.84387011
O 6.57928895 9.53209081 5.57090505
O 2.97255767 14.51627069 0.87800730
O 0.19110772 15.95509191 7.11442048
O 5.55195776 8.34317278 2.18599914

O 1.45372725 7.91459263 7.13703606
O 7.00971916 0.05304047 2.36537178
O -0.40091390 2.81953281 3.70553644
O 3.16450938 5.17949499 8.44285564
O 3.94104160 10.85100271 1.04349926
O 7.52865050 13.19419137 5.77626187
O 5.49847543 0.29816845 5.51507307
O 1.71801722 7.54324298 0.78753771
O 5.26217666 8.37053529 8.64995530
O 1.65420679 15.70772315 3.96026877
S -0.36316011 3.44624598 7.76659906
S 5.24986812 4.88752314 3.17362491
S 1.99638386 11.45939052 6.39625864
S 7.49939585 12.56350440 1.74043186
S 6.15862485 3.81578292 6.56773488
S 2.65653347 4.16928418 1.88197331
S 4.57880633 11.88113744 7.60459494
S 0.99843970 12.18124542 2.89291874
Fe 6.69243985 2.89802280 8.53839232
Fe 3.10988026 5.12947245 3.85806775
Fe 4.03970693 10.95448514 5.63913836
Fe 0.43039514 13.10201334 0.93014703
Fe 7.61285290 2.12126734 6.34586525
Fe 3.99819020 5.95559182 1.63297977
Fe 3.13263464 10.18263367 7.83986908
Fe -0.44816064 13.87185038 3.14719604
H 5.90357718 5.90894570 3.77540101

5.2 Complex 5

5.2.1 Fe–μ–Fe site

177
Converted from jawg015a-out.cell by c2x
H 4.06039940 9.51238624 2.45409951
H 8.51151618 1.09430002 10.82535350
H 9.58571486 4.20740823 4.24466593
H 2.88213715 6.29042849 9.13799363
H 5.71620746 8.92594104 2.47679698
H 6.85385755 1.67612797 10.83616565
H 7.93592347 3.60286076 4.23979487
H 4.53999629 6.86868051 9.16651013
H 5.51691170 8.09268618 0.31790486
H 7.08563399 2.46464611 13.00027093
H 8.17173755 2.78656645 6.40396105

H 4.35369626 7.71010534 7.01262935
H 3.93940695 8.85789784 0.20877809
H 8.66503740 1.70075650 13.06064796
H 9.75393180 3.54449418 6.48251814
H 2.77601403 6.94520222 6.89513986
H 0.07080894 9.69714900 1.00647491
H 12.49195688 0.74496727 12.41478613
H 13.65326259 4.30722813 5.68954652
H -1.08243767 6.13593952 7.68040927
H 0.61978720 8.28508820 0.08136059
H 11.94301295 2.17140105 13.31443049
H 13.06625339 2.89766974 6.59597307
H -0.53691521 7.56346610 6.77439279
H 1.79666026 9.45958797 0.67433528
H 10.76419632 0.98475888 12.74393820
H 11.92216633 4.10708800 6.01656563
H 0.64343130 6.38210058 7.34936198
H 14.05563618 6.93511652 3.70470905
H -1.46394316 3.41382458 9.66817835
H -0.40151668 1.75146110 2.95948855
H 12.91550915 8.70093471 10.40123935
H 13.83664574 6.68722832 1.96514962
H -1.24258272 3.68940360 11.40344395
H -0.18633123 1.51342744 4.69951252
H 12.69920818 8.93197675 8.65995407
H 13.48965798 8.27019678 2.68413714
H -0.93379748 2.08557365 10.71452708
H 0.19171342 3.08404885 3.97190546
H 12.33653710 7.35973399 9.39428729
H 2.18000693 9.79314938 3.57128688
H 10.34458426 0.63643811 9.84566793
H 11.51138749 4.49147299 3.16482173
H 1.02558120 5.99123108 10.22336926
H 1.23213769 8.83473496 4.69334974
H 11.30494477 1.56834325 8.71151400
H 12.41841478 3.54854794 1.99517094
H 0.08757447 6.92539784 11.37492639
H 0.42040993 10.00501997 3.63566424
H 12.09910935 0.39233120 9.77307573
H 13.27394822 4.67161005 3.06796470
H -0.73434197 5.78710289 10.28850195
H 2.47748018 3.99973369 12.44577852
H 9.97391241 6.53121541 0.94557951
H 8.84505851 9.17167787 7.62663530
H 3.65910363 1.31382139 5.76705780

H 0.78365783 3.89496612 12.94359728
H 11.65123605 6.62810979 0.39433740
H 10.54008916 9.08972847 7.12695268
H 1.96654713 1.39274264 6.27384856
H 1.73111055 2.40842189 12.71704368
H 10.73170474 8.12140231 0.68326946
H 9.61109283 7.58998225 7.34469311
H 2.89114228 2.89445479 6.04334397
H 5.93188161 1.50427638 0.94616712
H 6.45562335 9.04934355 12.52907700
H 7.66283776 6.73701203 5.77860040
H 4.85032240 3.75004190 7.61495858
H 6.47822277 2.33214465 2.41727476
H 5.91545942 8.27895725 11.02311722
H 7.11235792 7.57156973 4.31347997
H 5.39257655 2.91527326 9.08438613
H 6.86285744 3.00158133 0.82861618
H 5.52180607 7.54586253 12.58081738
H 6.73921040 8.24134006 5.90347561
H 5.76973214 2.24399826 7.49589586
H 3.35944040 1.38098368 1.68672617
H 9.01245105 9.21245992 11.72593969
H 10.23243834 6.60998400 5.01144409
H 2.27556429 3.87939400 8.37531750
H 2.42890846 2.75731238 2.31144243
H 9.95203642 7.84349362 11.10014466
H 11.15769343 7.99001769 4.38725359
H 1.35065456 2.50318551 9.00797039
H 3.77015553 2.10270027 3.25771481
H 8.61343953 8.50016810 10.14856030
H 9.81109171 7.33874072 3.44738495
H 2.70011073 3.15725182 9.94195988
C 4.68894882 8.64994904 2.24150042
C 7.88587026 1.95616294 11.04879308
C 8.96878035 3.33795086 4.46292033
C 3.51607098 7.14986679 8.93045840
C 4.54923641 8.18314530 0.80681747
C 8.04850435 2.39844266 12.49567195
C 9.13043989 2.87181834 5.89579036
C 3.38310596 7.61998553 7.49673849
C 1.45132806 5.20397418 1.61932026
C 11.20394860 5.32292396 11.72703393
C 12.15887946 10.44216352 5.06031036
C 0.34598459 0.05313002 8.31749618
C 1.97172774 5.68153972 4.02181435

C 10.84699973 4.70308209 9.28562843
C 11.66791235 0.34478412 2.65748495
C 0.82285375 10.14013347 10.72113133
C 6.46786450 5.64965339 1.94974180
C 5.97242753 4.81276566 11.46424830
C 7.19758866 0.35602076 4.74657567
C 5.30695575 10.13384034 8.63982232
C 5.02828051 4.64469840 3.90664077
C 7.20401715 5.92418813 9.47235416
C 8.56462356 9.88285596 2.81106489
C 3.94373038 0.61198964 10.57565596
C 0.86210843 8.95403705 0.90462576
C 11.69765971 1.48741913 12.50368277
C 12.84346673 3.58268742 5.78003197
C -0.29277445 6.88165314 7.58694738
C 14.14531261 7.40140661 2.72519127
C -1.56892013 2.96881133 10.65619233
C -0.48114479 2.22856529 3.93412923
C 13.00037096 8.22230442 9.42744575
C 1.22986826 9.27970501 3.70038385
C 11.30251980 1.13169516 9.70839133
C 12.44881551 3.96473077 3.00013249
C 0.07904742 6.50632784 10.37073105
C 1.76666487 3.44430342 13.05154859
C 10.67012076 7.09321353 0.32877256
C 9.56213819 8.62792573 7.01611959
C 2.94307318 1.85910010 6.37718507
C 6.10490077 2.47603185 1.40514650
C 6.28171033 8.09711863 12.03176655
C 7.49190952 7.71147130 5.32399786
C 5.01643675 2.77631810 8.07231737
C 3.41758677 2.31190473 2.24942724
C 8.95979540 8.28476873 11.15753924
C 10.16956149 7.54421433 4.45439725
C 2.33895537 2.94786591 8.93670534
O 0.80355680 4.43122196 1.04815313
O 11.82150663 6.07840331 12.34740664
O 12.84707064 9.68470276 5.60287566
O -0.33664838 0.81781458 7.77719596
O 1.65764673 5.22447379 5.04108105
O 11.26451794 5.03742144 8.25782821
O 11.91127175 10.41305124 1.67232896
O 0.57517811 0.06616828 11.70801303
O 7.58527725 5.78342690 1.67883139
O 4.84346516 4.73407897 11.70240274

```
O 6.08237907 0.50745802 5.01359699
O 6.42165548 9.98366339 8.36685786
O 5.21850178 4.12604339 4.92314260
O 6.90337265 6.39744422 8.45973328
O 8.38213490 9.36438915 1.79238368
O 4.12943501 1.13718461 11.58981802
P 1.01733314 7.98572017 2.43911843
P 11.54539477 2.43674028 10.95503291
P 12.65871917 2.64451394 4.23355097
P -0.13247958 7.82607068 9.13526565
P 4.57614717 3.46095300 1.43455535
P 7.80736887 7.12154691 11.96120662
P 9.02017682 8.69501818 5.28108098
P 3.48637747 1.79586768 8.10965745
S 4.22037743 7.33898422 3.42801930
S 8.32728269 3.26000697 9.85756550
S 9.43492635 2.03303526 3.26713471
S 3.05791443 8.46022838 10.12110039
S 3.78839190 6.52171586 0.70496210
S 8.79525875 4.04990308 12.64449363
S 9.88541122 1.20649195 5.98673000
S 2.62564587 9.28403609 7.39879029
Fe 2.43989577 6.32583183 2.48997640
Fe 10.18775462 4.25178824 10.81632870
Fe 11.21571885 1.00672990 4.18683423
Fe 1.28540153 9.48435928 9.19251189
Fe 4.80506078 5.44238625 2.38451965
Fe 7.63910857 5.16368665 10.95900787
Fe 8.85809006 0.13236536 4.30971202
Fe 3.64744871 10.35698655 9.07937724
H 9.18146288 5.55586621 10.56499162
```

5.2.2 C_{basal} geminal to P

```
177
Converted from jawg015b-out.cell by c2x
H 4.07779593 9.57105870 2.44205792
H 8.44886304 1.10463666 10.92329340
H 9.53702011 4.43212042 4.44296187
H 3.03063042 6.39687855 9.06745631
H 5.75895110 9.03839169 2.46303085
H 6.78617383 1.67861128 10.92224731
H 7.89074760 3.83446519 4.32752673
H 4.69299499 6.96757825 9.10001946
H 5.59407938 8.17686954 0.31700682
```

H 7.00580908 2.51668189 13.07948961
H 8.02616362 2.88872603 6.44231534
H 4.50823164 7.81215610 6.94045046
H 4.01106669 8.92671183 0.18839535
H 8.59041335 1.76067116 13.16820615
H 9.59075976 3.65388792 6.64523133
H 2.92138906 7.07131824 6.82418142
H 0.17223395 9.75623237 1.01005809
H 12.44178459 0.95780006 12.36344678
H 13.55207596 4.49556350 5.78834843
H -0.92900877 6.24262386 7.59652680
H 0.68997432 8.33027883 0.08816552
H 11.91452795 2.38140989 13.28214625
H 13.00176823 3.05413965 6.66845696
H -0.39283233 7.67797721 6.69887728
H 1.88961544 9.50057804 0.64679756
H 10.72149041 1.20131601 12.72663981
H 11.82569830 4.25413288 6.12531469
H 0.79415838 6.49725217 7.25909680
H 14.02936568 7.13397456 3.75680053
H -1.43424123 3.58111758 9.60845004
H -0.35150442 1.77780354 2.95552753
H 12.93423700 8.94537940 10.41122497
H 13.83495035 6.90122633 2.01323050
H -1.23954351 3.81635776 11.35142796
H -0.15091004 1.48894125 4.69260171
H 12.71400570 9.21854257 8.67418010
H 13.45893239 8.47441325 2.74009730
H -0.85463748 2.24565056 10.62604007
H 0.25667852 3.07386821 4.00485191
H 12.36651904 7.62364422 9.37084508
H 2.25005772 9.87554390 3.56040122
H 10.35790046 0.81988623 9.81389110
H 11.48102057 4.70036741 3.23908064
H 1.14976052 6.08842346 10.15301574
H 1.33216666 8.89371432 4.68805133
H 11.27337215 1.80117423 8.68382727
H 12.36777114 3.72372396 2.08163077
H 0.23729301 7.05664436 11.29590000
H 0.48582382 10.04312683 3.63474495
H 12.12440482 0.65601895 9.73739760
H 13.25069983 4.83604785 3.14408943
H -0.61544231 5.92207181 10.23271112
H 2.58208546 4.05328467 12.34272757
H 9.97082840 6.65279945 1.03769611

H 8.90746797 9.42675251 7.54859188
H 3.69490058 1.36558402 5.72106108
H 0.89683837 3.96672012 12.86922565
H 11.66406704 6.66613271 0.52214391
H 10.60812973 9.36214352 7.06659152
H 2.00189476 1.43504763 6.22727210
H 1.82336359 2.46975557 12.62432458
H 10.79682500 8.20012252 0.75110372
H 9.69067196 7.85277199 7.28155121
H 2.92095208 2.94129790 6.00732304
H 5.94370531 1.55872250 0.92430604
H 6.62844179 9.10711677 12.49362709
H 7.76194597 6.99393597 5.71221651
H 4.86403363 3.79793735 7.57797633
H 6.53119559 2.39706388 2.37574433
H 6.02733016 8.28912487 11.03904332
H 7.21336139 7.82307330 4.23670409
H 5.39552474 2.98068757 9.06037784
H 6.89480555 3.04563728 0.77474828
H 5.68655517 7.61813241 12.63829842
H 6.83499200 8.49571016 5.83047507
H 5.80720013 2.30533987 7.48306055
H 3.40402481 1.46408252 1.68839210
H 9.15671153 9.22022872 11.69807414
H 10.32200538 6.85395507 5.00456886
H 2.29348781 3.90746974 8.32930759
H 2.48967379 2.84103542 2.33068055
H 10.07473415 7.85257476 11.04131859
H 11.28121716 8.21111822 4.39336389
H 1.36722562 2.52872059 8.95432029
H 3.84161194 2.17618248 3.25501972
H 8.71462654 8.51666940 10.12784539
H 9.95539632 7.55895848 3.41448787
H 2.70953628 3.18667535 9.89732407
C 4.74108244 8.73484287 2.22863587
C 7.81460850 1.96097426 11.14528263
C 8.91171921 3.55121847 4.57918850
C 3.66942763 7.25334070 8.86042948
C 4.62097871 8.26073519 0.79624109
C 7.96724105 2.42998099 12.57755354
C 9.01091006 3.00363788 5.99287856
C 3.53933483 7.73249994 7.42924934
C 1.52543679 5.26005883 1.63976148
C 11.06436784 5.40724769 11.72319586
C 12.17682223 10.69163193 5.15437007

C 0.40096485 0.10240188 8.21864950
C 2.08061006 5.73179203 4.01459215
C 10.50357277 4.96126212 9.34863577
C 11.55536766 0.51728945 2.73138739
C 0.98939117 10.24505184 10.65034049
C 6.58368443 5.66383237 1.95354600
C 6.01086551 4.96728992 11.41048281
C 7.13471828 0.50234392 4.67862068
C 5.48669980 10.25718993 8.61415331
C 5.08980558 4.72682994 3.90130530
C 7.50755350 5.97283076 9.46712159
C 8.49408688 9.93959825 2.76253443
C 3.97680017 0.63874957 10.53113333
C 0.95537231 9.00548537 0.89951561
C 11.65263059 1.70232348 12.47274332
C 12.75730548 3.75395754 5.87195264
C -0.14186350 6.99183265 7.50549448
C 14.12439676 7.61278748 2.78375431
C -1.52635526 3.10227986 10.58195067
C -0.42760850 2.22835072 3.94387059
C 13.02089846 8.49333451 9.42463728
C 1.31401746 9.33812331 3.69496959
C 11.29368547 1.35759073 9.67714926
C 12.40625082 4.15205336 3.08044756
C 0.21487599 6.62400156 10.29811110
C 1.87616173 3.50464972 12.95988602
C 10.70438956 7.16559920 0.42114513
C 9.63760753 8.88940563 6.95089182
C 2.97672351 1.90369114 6.33375720
C 6.14217596 2.53215525 1.36849768
C 6.42950870 8.13801169 12.03866993
C 7.58365839 7.96259032 5.24892559
C 5.03744429 2.83010104 8.04380989
C 3.47551027 2.39254610 2.25339767
C 9.08712075 8.29590137 11.12651003
C 10.28661574 7.77650017 4.42629306
C 2.35471884 2.97612058 8.89055579
O 0.83720331 4.49031958 1.11102709
O 11.76047078 6.16109684 12.26136171
O 12.83150956 9.92730082 5.72909666
O -0.24920420 0.87382509 7.64614941
O 1.76118378 5.27006940 5.03050403
O 10.82709502 5.43990935 8.34215062
O 11.87793467 10.63627667 1.74269999
O 0.64026201 0.09202525 11.64640807

```
O 7.71172087 5.71574331 1.70502373
O 4.88932017 4.83408988 11.66256966
O 6.02083376 0.59078602 4.97983760
O 6.60883499 10.13462533 8.35648381
O 5.23492297 4.21704658 4.93045789
O 7.38050746 6.49230942 8.44102868
O 8.01045222 8.95819826 2.26156409
O 4.15321824 1.16414937 11.54714198
P 1.12112384 8.03980603 2.43429041
P 11.48144349 2.65868215 10.93266179
P 12.59055652 2.83085949 4.31467845
P 0.01769582 7.93706736 9.05389666
P 4.62928163 3.53713766 1.42323632
P 7.94565706 7.14253750 11.95907241
P 9.11728272 8.93444509 5.21219545
P 3.51667424 1.83513525 8.06638291
S 4.31695763 7.41027542 3.41300610
S 8.26101284 3.27338678 9.95229572
S 9.42350353 2.32615487 3.32601879
S 3.21025997 8.55968312 10.05546151
S 3.88021783 6.59101998 0.69079238
S 8.71190074 4.09776151 12.67110871
S 9.78961350 1.35111693 6.05212274
S 2.80189663 9.40397512 7.33731016
Fe 2.53966259 6.37946850 2.48277402
Fe 10.04337416 4.29995107 10.87591768
Fe 11.13547458 1.20007968 4.26188115
Fe 1.44329860 9.59557935 9.11529363
Fe 4.90344368 5.51274382 2.37020401
Fe 7.68238420 5.17117863 10.99539196
Fe 8.80140809 0.31757661 4.24179262
Fe 3.81617535 10.46053276 9.03171677
H 9.03563976 10.68360043 2.10066462
```

5.2.3 C_{basal} *trans* to P

```
177
Converted from jawg015d-out.cell by c2x
H 4.09886009 9.57339622 2.49717085
H 8.41670017 1.08223918 10.93947537
H 9.51577608 4.33094150 4.34554799
H 2.99516329 6.36690524 9.14759919
H 5.75957025 8.99727500 2.49946558
H 6.75577321 1.65744841 10.93693235
H 7.84811384 3.77860796 4.34222231
```

H 4.65494338 6.93965498 9.16647781
H 5.54571890 8.19952429 0.33144966
H 6.97176062 2.46058927 13.10540796
H 8.05882908 2.87225256 6.45974379
H 4.45115321 7.77597821 7.00452090
H 3.95591152 8.93710532 0.25260386
H 8.55768512 1.71466918 13.18099711
H 9.63377269 3.63240057 6.57772899
H 2.86422093 7.03276999 6.91001471
H 0.07418039 9.71455050 1.04155190
H 12.41236756 0.91781540 12.40411633
H 13.53432070 4.52188500 5.85984987
H -1.00698093 6.23887374 7.67375244
H 0.64952830 8.30949397 0.12329703
H 11.86456496 2.33190003 13.32809730
H 12.95283112 3.07173226 6.70617520
H -0.44013253 7.65842052 6.76799076
H 1.80319808 9.52351560 0.68398856
H 10.68890557 1.13869589 12.75755350
H 11.80109012 4.29831192 6.16346840
H 0.72067028 6.45459335 7.33608279
H 13.98453901 7.07579299 3.82593373
H -1.45783525 3.59688554 9.64531131
H -0.33344374 1.92463272 2.97385709
H 12.87284441 8.88435137 10.48510623
H 13.77092497 6.82297898 2.08609861
H -1.24795375 3.83141055 11.38688918
H -0.11644597 1.64723512 4.70803832
H 12.65459416 9.14180800 8.74909226
H 13.38294495 8.39490441 2.80178959
H -0.87082314 2.26050903 10.65716293
H 0.26876322 3.23393720 4.00921207
H 12.28402180 7.56069803 9.46045239
H 2.22233108 9.87159306 3.58651360
H 10.30975095 0.78541980 9.85527462
H 11.46796909 4.79417419 3.30011835
H 1.11299552 6.06296421 10.21702506
H 1.28194881 8.91861068 4.72257604
H 11.24260867 1.74923806 8.72405098
H 12.37424453 3.86009303 2.12209761
H 0.18542807 7.00916910 11.36657728
H 0.45937237 10.06596075 3.65238143
H 12.07298130 0.60154559 9.79325197
H 13.23313202 4.94992538 3.22222123
H -0.65054281 5.87487181 10.28826901

H 2.54173956 4.11730917 12.38980809
H 9.95410956 6.49239877 1.03180839
H 8.88154914 9.32150576 7.66524641
H 3.66656447 1.38292761 5.74441458
H 0.85750677 4.02575427 12.91718031
H 11.64163455 6.60195339 0.52222942
H 10.56691632 9.23371847 7.14556139
H 1.97529010 1.46383440 6.25031581
H 1.78898024 2.53044347 12.67287440
H 10.70995043 8.08749162 0.79647306
H 9.63630369 7.73428349 7.38382946
H 2.90502023 2.96520758 6.03566598
H 5.91153325 1.54785816 0.95154879
H 6.59839350 9.09818961 12.49442952
H 7.69976645 6.86977408 5.80971073
H 4.83665524 3.82348870 7.60469535
H 6.45904192 2.36993206 2.42835441
H 6.04503478 8.28408676 11.01683420
H 7.14977535 7.69845728 4.33876418
H 5.37041148 2.99814238 9.08396464
H 6.86596469 3.03548575 0.84680809
H 5.64018722 7.61578215 12.60023586
H 6.75847075 8.36123080 5.92804059
H 5.77758605 2.32797530 7.50291352
H 3.37056961 1.44535367 1.71393072
H 9.13709897 9.20369744 11.74843100
H 10.27741926 6.78077324 5.05490984
H 2.27400225 3.92737384 8.36205158
H 2.43417429 2.81910170 2.33408764
H 10.07322276 7.83962329 11.11097950
H 11.18528135 8.16646718 4.40935571
H 1.33699106 2.54528426 8.96653899
H 3.78068177 2.17271461 3.28042479
H 8.72660140 8.49669956 10.17147647
H 9.84355401 7.49425828 3.48359114
H 2.67310939 3.18976280 9.92762696
C 4.73195015 8.71785716 2.27131803
C 7.78349662 1.93765064 11.16389764
C 8.87364366 3.47574956 4.54610636
C 3.62948393 7.22530959 8.93665185
C 4.58277912 8.26318185 0.83421137
C 7.93332028 2.39302215 12.60066262
C 9.02446474 2.96749243 5.96747179
C 3.48771399 7.69875944 7.50440040
C 1.48992893 5.26618015 1.67324025

C 11.02985722 5.37902521 11.76945813
C 12.20656406 10.63155379 4.99066420
C 0.39317437 0.07969557 8.23158932
C 2.04089570 5.73324726 4.04704246
C 10.47459815 4.93361096 9.39225259
C 11.50399946 0.37032403 2.56940073
C 0.92964645 10.22314355 10.72207676
C 6.52958493 5.69982176 1.98741653
C 5.98479061 4.96197847 11.45049688
C 7.10573248 0.44610875 4.75968320
C 5.42834392 10.23874853 8.68782977
C 5.06020362 4.70812573 3.92491203
C 7.46111266 5.95494505 9.51226005
C 8.54841651 10.03325695 2.88031388
C 3.94581618 0.66988930 10.55441341
C 0.88658863 8.99533967 0.93458979
C 11.61584472 1.65379856 12.51340273
C 12.72940468 3.78879427 5.91923873
C -0.20479457 6.97111410 7.57882269
C 14.06116564 7.54446477 2.84662940
C -1.54106754 3.11829876 10.61952675
C -0.40760604 2.38142534 3.95985912
C 12.95378937 8.41853723 9.50473558
C 1.27779601 9.35133744 3.72420933
C 11.25132538 1.31225072 9.72047369
C 12.39920100 4.25727040 3.13520886
C 0.17076240 6.58531849 10.36464716
C 1.83817562 3.56631994 13.00668249
C 10.66077506 7.06213851 0.43534652
C 9.58816326 8.77007635 7.05104151
C 2.95292731 1.92594165 6.35778099
C 6.09648180 2.51632370 1.41275837
C 6.40939605 8.13169812 12.03107953
C 7.52056274 7.84117980 5.35210220
C 5.01054077 2.85351786 8.06705067
C 3.42522708 2.37914838 2.27262908
C 9.08168688 8.27802320 11.17703429
C 10.20359272 7.70843290 4.48791787
C 2.32687338 2.99034806 8.91518928
O 0.81199169 4.49123573 1.13997263
O 11.71632311 6.13935011 12.31041161
O 12.92864223 9.85483937 5.46113204
O -0.27234793 0.82192039 7.64100016
O 1.72932967 5.26715750 5.06401659
O 10.78849792 5.41110123 8.38199010

```
O 11.58852655 9.93623096 1.99887438
O 0.60916571 0.13213799 11.65151317
O 7.65289716 5.82598307 1.73903082
O 4.86175279 4.84680842 11.70709690
O 5.98559466 0.56909164 5.02187901
O 6.54738461 10.10987523 8.42176364
O 5.22909152 4.19694133 4.94926858
O 7.30226195 6.47771383 8.49216646
O 8.36808785 9.48863145 1.87650388
O 4.10026547 1.20462713 11.56953603
P 1.07132231 8.04321014 2.47603822
P 11.44306403 2.61721933 10.97354858
P 12.58414118 2.90697879 4.33817036
P -0.03169607 7.90726388 9.13216367
P 4.58527299 3.52330440 1.44889713
P 7.92296975 7.12726420 11.99334868
P 9.04057516 8.83734107 5.32185861
P 3.49309092 1.85394160 8.09022065
S 4.27976099 7.39870210 3.45295574
S 8.23413238 3.25564241 9.98110809
S 9.29664407 2.19613949 3.30987002
S 3.17127712 8.53050878 10.13383772
S 3.84235565 6.59249241 0.72549610
S 8.67463700 4.06235772 12.70968994
S 9.78579197 1.30442984 6.03199051
S 2.74914949 9.37031969 7.41552122
Fe 2.49921048 6.38550107 2.51843326
Fe 10.01559811 4.26919988 10.91743531
Fe 11.12969583 1.19769446 4.17030568
Fe 1.39723442 9.55673964 9.19819431
Fe 4.85704285 5.50456501 2.40128421
Fe 7.65641807 5.14926196 11.03230636
Fe 8.77257745 0.26415334 4.32243132
Fe 3.75775184 10.43715245 9.10960494
H 11.87751342 1.35414712 2.10715547
```

5.2.4 C_{basal} *cis* to P

```
177
Converted from jawg015f-out.cell by c2x
H 4.08978632 9.57823268 2.42600603
H 8.41230775 1.02469639 10.94253671
H 9.52911242 4.26109954 4.34270347
H 2.99174516 6.31725385 9.07221423
H 5.75474094 9.01944200 2.43534136
```

H 6.75010052 1.59180327 10.93009762
H 7.86746752 3.69595490 4.26677176
H 4.65153081 6.88768453 9.08495340
H 5.54583717 8.19141874 0.26839585
H 6.95780882 2.43262053 13.08761583
H 7.98817181 2.82088646 6.40704628
H 4.44334477 7.69927699 6.91352433
H 3.94910306 8.91274549 0.18706114
H 8.55165936 1.70450833 13.18323694
H 9.55451196 3.59246603 6.58677665
H 2.84748146 6.97854601 6.83249718
H 0.08369532 9.69667661 0.95888350
H 12.38179394 0.86895566 12.38252235
H 13.55316793 4.66431265 5.60708429
H -1.00715194 6.17966885 7.61275718
H 0.65378423 8.27196957 0.06376168
H 11.85346466 2.29998682 13.29096394
H 13.07862723 3.27862035 6.60416878
H -0.50551552 7.62470429 6.71277611
H 1.81359815 9.48168327 0.61898668
H 10.66388635 1.11771032 12.73872081
H 11.84825204 4.40415129 6.03037224
H 0.70623381 6.44240630 7.23033686
H 13.96860887 7.00689371 3.72008554
H -1.45840729 3.54226626 9.62897606
H -0.34314971 1.88604392 2.93622804
H 12.87080379 8.85365653 10.38834506
H 13.77265509 6.78486206 1.97203318
H -1.26006262 3.79326206 11.36889567
H -0.09402710 1.68169559 4.67504195
H 12.65509449 9.09492791 8.64709346
H 13.37495917 8.34313042 2.72168884
H -0.88609839 2.21199404 10.65887332
H 0.26028826 3.24089808 3.91624072
H 12.27824367 7.52149945 9.37220569
H 2.17031431 9.87264968 3.53209017
H 10.30225283 0.74043373 9.81429619
H 11.35151224 4.63445997 3.13801422
H 1.08179199 6.00769904 10.15527905
H 1.25015507 8.89263796 4.66045098
H 11.22883397 1.71694456 8.69191173
H 12.22134735 3.61736969 2.00590876
H 0.18181126 6.98771725 11.29972560
H 0.40258658 10.03142664 3.59844899
H 12.06622753 0.56322831 9.74847079

H 13.10413213 4.83134388 2.95320590
H -0.68178179 5.85577572 10.24305513
H 2.56053846 3.97616171 12.36393634
H 9.93998723 6.62858394 0.98579313
H 8.84083498 9.23855472 7.62242863
H 3.66901638 1.29555307 5.72119837
H 0.87184030 3.89853002 12.88096397
H 11.62902657 6.70097196 0.46914450
H 10.53448206 9.16001651 7.11178604
H 1.97785296 1.36229053 6.23003024
H 1.79665392 2.39709735 12.65999555
H 10.70799063 8.20421890 0.68919051
H 9.60252595 7.66073341 7.31553584
H 2.89691337 2.87048534 6.01518099
H 5.93624097 1.54571141 0.93988877
H 6.55570598 9.04693697 12.41759914
H 7.60039531 6.89786723 5.78381791
H 4.82578041 3.73264916 7.58602569
H 6.48140268 2.36977265 2.41701671
H 6.02075139 8.23056213 10.93429472
H 7.07694963 7.72322956 4.30513261
H 5.36068398 2.91580217 9.06993902
H 6.88279394 3.03678549 0.83368080
H 5.61290008 7.55316992 12.51319626
H 6.69921951 8.41499628 5.88665911
H 5.77499507 2.24501378 7.49008727
H 3.37660278 1.44428182 1.69290805
H 9.11824342 9.15949633 11.64717599
H 10.15448199 6.69559811 4.96180443
H 2.26869241 3.83555528 8.33227447
H 2.44244126 2.82688990 2.29352539
H 10.05225023 7.77600544 11.04903423
H 11.08644213 8.05426051 4.29927799
H 1.32783039 2.45457534 8.93035583
H 3.77204494 2.17812198 3.26107923
H 8.71797107 8.41808212 10.08282889
H 9.67063253 7.42904612 3.42030897
H 2.65649860 3.09914584 9.90143310
C 4.73015106 8.72557479 2.20823286
C 7.77562735 1.88110410 11.15588730
C 8.88473050 3.40316673 4.52399413
C 3.62602847 7.17416128 8.85423056
C 4.58487735 8.25396881 0.77542312
C 7.92052894 2.36036093 12.58559685
C 8.97414018 2.91952281 5.95818286

C 3.48155809 7.63988867 7.41958587
C 1.50715015 5.28544567 1.58978570
C 10.99758157 5.31601339 11.75984941
C 12.20679593 10.46782000 5.25544184
C 0.40324683 0.00177467 8.23330420
C 2.05029332 5.71997184 3.99789184
C 10.45458190 4.88313880 9.34826492
C 11.72520030 0.36724941 2.89102215
C 0.94046373 10.18319327 10.63492758
C 6.53907476 5.70222052 1.94798483
C 5.96777303 4.89934026 11.39772058
C 7.15716748 0.39210411 4.73961369
C 5.42856181 10.20015066 8.58524982
C 5.04641823 4.70746171 3.89592840
C 7.44926682 5.89279961 9.45353884
C 8.64086076 10.01089746 2.80054811
C 3.94172810 0.58158466 10.53892984
C 0.88896897 8.96697027 0.86755868
C 11.59529470 1.61703002 12.48384279
C 12.78149525 3.91093288 5.76860652
C -0.22498346 6.93180395 7.50505517
C 14.05282156 7.49231726 2.74954719
C -1.55068529 3.07351711 10.60689956
C -0.40763806 2.38100696 3.90242783
C 12.94908803 8.37921827 9.41198626
C 1.23587766 9.33299837 3.66604903
C 11.24181973 1.27147374 9.68421682
C 12.28969145 4.11032083 2.97244589
C 0.15267234 6.55287447 10.30267647
C 1.84992351 3.43598871 12.98278744
C 10.65221858 7.16568486 0.36598722
C 9.55564183 8.70344714 7.00311274
C 2.95221492 1.83111925 6.33693535
C 6.11602133 2.51493571 1.40180593
C 6.38034439 8.07977499 11.95064380
C 7.44842152 7.86985993 5.31785052
C 5.00366671 2.76564545 8.05256212
C 3.43162180 2.38032132 2.24753374
C 9.06259994 8.22143727 11.09603867
C 10.09265011 7.62899731 4.40308150
C 2.31783006 2.89924006 8.88674594
O 0.84215563 4.54032160 1.00194281
O 11.66235152 6.06109575 12.34731621
O 12.58141696 9.33309616 5.38436449
O -0.25423212 0.75644647 7.64491995

O 1.74153444 5.24537635 5.01168345
O 10.76188583 5.35760974 8.33438025
O 12.08276399 10.44288061 1.91579637
O 0.63652648 0.04709147 11.65768363
O 7.65897920 5.83279346 1.68460157
O 4.84702511 4.76963270 11.65539252
O 6.04240022 0.53192435 5.01293586
O 6.54936930 10.07685302 8.32437456
O 5.18250689 4.18117751 4.91772635
O 7.29434659 6.42022874 8.43507977
O 8.47627940 9.49302852 1.77960865
O 4.08591059 1.10989544 11.55852085
P 1.06068171 8.02347998 2.41550061
P 11.42998716 2.57141724 10.94086204
P 12.57870975 2.88332510 4.27543297
P -0.03884772 7.86447918 9.05606605
P 4.60111958 3.51840737 1.42778482
P 7.89919774 7.08347535 11.92244766
P 9.00814006 8.80126854 5.27452764
P 3.48965035 1.76216896 8.07090288
S 4.28354327 7.41136063 3.40076163
S 8.22117796 3.18712509 9.95370083
S 9.38321090 2.11135776 3.32418299
S 3.17219810 8.48908740 10.04292264
S 3.85581798 6.57910863 0.68284045
S 8.65320400 4.03356672 12.66808420
S 9.74611018 1.26390433 6.08111247
S 2.74881923 9.31298525 7.32079877
Fe 2.50820093 6.38364403 2.47146595
Fe 9.99749348 4.21855334 10.87763959
Fe 11.18981159 1.10351884 4.35781327
Fe 1.39705645 9.51436377 9.10799655
Fe 4.86803748 5.50684964 2.36819386
Fe 7.63845638 5.09509024 10.98046386
Fe 8.82700222 0.18845964 4.34182396
Fe 3.75668709 10.39059714 9.00581436
H 12.68418503 11.27919108 5.90626124

5.2.5 C_{apical}

177
Converted from jawg015h-out.cell by c2x
H 4.07997002 9.51044247 2.42538475
H 8.38932021 0.95453487 10.92887433
H 9.50649735 4.21542160 4.29448183

H 2.98464221 6.19653053 9.08735628
H 5.73078796 8.93993921 2.44125363
H 6.73200399 1.53507098 10.91345397
H 7.83383749 3.67641802 4.33542097
H 4.64376703 6.77171431 9.09579201
H 5.53282657 8.11209581 0.27706727
H 6.93990685 2.34399052 13.08451129
H 8.07039044 2.80768664 6.47381965
H 4.42904489 7.57447380 6.91980848
H 3.94134246 8.84620522 0.18140052
H 8.53353337 1.61376030 13.16908097
H 9.62282349 3.62427719 6.55848131
H 2.83562640 6.84411477 6.84456920
H 0.08628570 9.65036243 0.96652401
H 12.40746871 0.83016697 12.39954637
H 13.51219328 4.42564473 5.70886222
H -1.02502965 6.05982551 7.63468867
H 0.65507952 8.23801183 0.05349388
H 11.83728626 2.24315173 13.31121795
H 12.95366797 3.00532889 6.61544873
H -0.45699679 7.46805045 6.71449548
H 1.81574290 9.43921665 0.62898105
H 10.67801320 1.03787438 12.74167353
H 11.78458698 4.20310264 6.05275959
H 0.70258681 6.26529394 7.28606524
H 13.93425139 7.00342792 3.72742740
H -1.45960459 3.46587856 9.62311077
H -0.33436781 1.76003195 2.92987456
H 12.83750615 8.72715570 10.40096268
H 13.70801956 6.78041204 1.98281827
H -1.22646423 3.70612828 11.36385298
H -0.09566575 1.53973271 4.67149937
H 12.60199142 8.96692473 8.66192302
H 13.35171470 8.34840267 2.72680978
H -0.86834621 2.13198223 10.63465503
H 0.24551181 3.10896782 3.92480424
H 12.24881708 7.39138265 9.39204474
H 2.22240520 9.79229915 3.51466045
H 10.26366951 0.67223317 9.85423920
H 11.36656361 4.56296096 3.15092183
H 1.11427842 5.91525849 10.18421499
H 1.27754600 8.84769201 4.65255707
H 11.20336345 1.61885810 8.71533444
H 12.31844473 3.62059847 2.01604019
H 0.17447539 6.87162708 11.31609138

H 0.46042957 9.99705860 3.57610296
H 12.02515727 0.47339988 9.79297836
H 13.12670451 4.77787233 3.08617647
H -0.64748610 5.71876670 10.24659317
H 2.53288849 3.90076651 12.39681523
H 9.93617779 6.56704619 0.96268779
H 8.87761299 9.09845847 7.68980465
H 3.66743623 1.28987690 5.72602102
H 0.84332314 3.81158225 12.91063248
H 11.62626510 6.65674955 0.44896012
H 10.55138656 9.03083286 7.12039594
H 1.97411681 1.34863440 6.23362303
H 1.77691625 2.31622380 12.68108883
H 10.69213999 8.15121797 0.67664929
H 9.64163997 7.52435316 7.37083202
H 2.88504672 2.85913208 6.01583056
H 5.91740931 1.49091813 0.91666938
H 6.55704780 8.97823077 12.43737771
H 7.67519483 6.65488062 5.79424248
H 4.80764318 3.73583295 7.60006714
H 6.44997997 2.30116838 2.39642714
H 5.99969886 8.14889903 10.96801160
H 7.12019170 7.49913293 4.33045084
H 5.35982861 2.90224564 9.07021149
H 6.86144134 2.98972437 0.81762243
H 5.61437701 7.48493864 12.55801497
H 6.70944657 8.12978765 5.92870109
H 5.75829552 2.24852885 7.48192541
H 3.34974210 1.38713532 1.69113868
H 9.11481923 9.07964621 11.65935357
H 10.24484917 6.61276322 5.00785997
H 2.25242700 3.83062864 8.35222525
H 2.43267582 2.77528649 2.31331459
H 10.03761757 7.69177972 11.04882787
H 11.13471699 8.02169569 4.40637278
H 1.32457520 2.44714457 8.96592220
H 3.78110208 2.10731468 3.25094552
H 8.69566232 8.34098650 10.09910540
H 9.79667872 7.35684978 3.45702637
H 2.66594544 3.09718031 9.91623729
C 4.70905764 8.65081212 2.20693868
C 7.75875228 1.81424540 11.14627287
C 8.86054513 3.37468345 4.53805702
C 3.61741484 7.05306809 8.86392998
C 4.56764896 8.18232014 0.77476044

C 7.90273674 2.27823871 12.58132023
C 9.03159305 2.92126806 5.97458057
C 3.46857562 7.51082195 7.42759526
C 1.48895115 5.20408278 1.58710739
C 10.98522669 5.27131145 11.76909119
C 12.12065250 10.42951976 5.03551216
C 0.38855470 0.00589219 8.25603319
C 2.01905187 5.65710612 3.99551057
C 10.46282785 4.80123152 9.36515336
C 11.56471443 0.45010113 2.68753730
C 0.92318746 10.04513368 10.64353003
C 6.51350998 5.63825312 1.94069051
C 5.95643536 4.82579720 11.41201160
C 6.93092741 0.45293435 4.67158890
C 5.42000968 10.06925376 8.58318691
C 5.03884199 4.63947382 3.88722170
C 7.43421661 5.82465966 9.46705795
C 8.45444899 9.84543121 2.90885394
C 3.93516911 0.57555810 10.55530246
C 0.89070613 8.92068400 0.86769393
C 11.60134643 1.55788971 12.49940634
C 12.71191029 3.69150644 5.80559419
C -0.21944117 6.78719499 7.52984689
C 14.01362921 7.48410712 2.75409680
C -1.53351535 2.99365394 10.60109116
C -0.41191723 2.24129408 3.90277953
C 12.91234694 8.25449717 9.42331370
C 1.27423401 9.27855931 3.65350237
C 11.20964270 1.18996702 9.71504544
C 12.31746164 4.05332936 3.01444133
C 0.16895114 6.43529019 10.31926700
C 1.82342099 3.35306916 13.01108542
C 10.64559065 7.11395986 0.34755144
C 9.57210987 8.56238836 7.04877956
C 2.94608435 1.82206459 6.34341542
C 6.09723193 2.45887737 1.38044875
C 6.37355436 8.00726175 11.98039628
C 7.48034949 7.62812507 5.34938201
C 4.99100779 2.76426372 8.05554287
C 3.41722280 2.32104109 2.24800244
C 9.05080004 8.14185084 11.10862902
C 10.15736448 7.55276705 4.46443661
C 2.31261867 2.89460725 8.90635547
O 0.83283073 4.44856501 1.00152956
O 11.64010197 6.03675217 12.34125741

O 12.77292160 9.64255229 5.58157221
O -0.26625689 0.75492874 7.66229053
O 1.70369761 5.19139539 5.01173733
O 10.78113585 5.26031360 8.34668139
O 11.88336206 10.48157081 1.63779829
O 0.59972977 0.01635018 11.69093320
O 7.63353967 5.77269714 1.67931864
O 4.83656213 4.69136913 11.67088527
O 5.84416975 0.56940196 4.17743107
O 6.54282757 9.94266190 8.32337201
O 5.19640331 4.12250682 4.91060699
O 7.27550091 6.34272350 8.44369000
O 8.25170306 9.30808784 1.90115952
O 4.09493617 1.08927383 11.57892073
P 1.06621333 7.96795236 2.41033656
P 11.41805811 2.50361364 10.95484745
P 12.53722514 2.74486178 4.25948176
P -0.03730200 7.74119113 9.07019544
P 4.58064105 3.45353951 1.41665318
P 7.88932771 7.00715158 11.94215852
P 8.98272501 8.64179124 5.33341936
P 3.47765678 1.75804793 8.07970990
S 4.25800362 7.33933233 3.39725525
S 8.21902609 3.12753168 9.95861767
S 9.25874506 2.04275597 3.35321117
S 3.16591555 8.37176983 10.04805845
S 3.83088676 6.51059890 0.67691714
S 8.63778267 3.95156246 12.68034595
S 9.84030969 1.28244826 6.09157266
S 2.73320040 9.18308513 7.32329918
Fe 2.48355478 6.31078688 2.46655625
Fe 9.99119523 4.15537299 10.89553679
Fe 11.10843359 1.08008578 4.22987661
Fe 1.39081272 9.39218378 9.11375402
Fe 4.84452326 5.43824056 2.36220929
Fe 7.62784498 5.02692640 10.99328130
Fe 8.69552694 0.15938027 4.43287253
Fe 3.75199317 10.26546228 8.99913829
H 7.04981362 0.52268572 5.80631858

5.2.6 O_{apical}

177
Converted from jawg015i-out.cell by c2x
H 4.12259641 9.50727482 2.45851899

H 8.42842047 1.03151895 10.91592300
H 9.59467897 4.30826985 4.26013797
H 3.01230273 6.23862781 9.10517871
H 5.77759541 8.92653110 2.47781930
H 6.76942758 1.60633018 10.91124656
H 7.93418948 3.73085034 4.27065382
H 4.67046342 6.81804403 9.12147572
H 5.57259959 8.10837200 0.31246705
H 6.98683518 2.42106113 13.07997631
H 8.14289140 2.88051314 6.40963937
H 4.46330677 7.62126422 6.94823811
H 3.98414166 8.85206570 0.22088715
H 8.57747256 1.68130239 13.15885560
H 9.69686156 3.70140867 6.52306389
H 2.86604244 6.89845209 6.86586661
H 0.10193340 9.65877017 1.00850841
H 12.45535034 0.90511026 12.36731885
H 13.59453185 4.49330491 5.70646052
H -1.00210734 6.09826182 7.65779258
H 0.66220685 8.24619627 0.08995634
H 11.89140249 2.31728507 13.28502862
H 13.03528540 3.07817323 6.62469463
H -0.44747183 7.50869936 6.73372820
H 1.82907899 9.44600588 0.65671561
H 10.73014798 1.10894024 12.72700210
H 11.86695769 4.26576674 6.04657133
H 0.72375597 6.31264730 7.30166167
H 14.03044757 7.04465836 3.75630176
H -1.48440554 3.51704549 9.62020098
H -0.31944322 1.78621794 2.94529857
H 12.92592001 8.81079984 10.40379721
H 13.79855187 6.82419949 2.01296721
H -1.26223642 3.74489927 11.36465642
H -0.08415145 1.55210758 4.68519009
H 12.69348967 9.04727224 8.66190543
H 13.44562428 8.39129953 2.75984870
H -0.89552126 2.17796341 10.62428878
H 0.26171069 3.12909220 3.95149876
H 12.33996246 7.47342288 9.39558520
H 2.24339642 9.79883742 3.54574655
H 10.31229822 0.74647518 9.82943373
H 11.45339365 4.61622815 3.16300361
H 1.13766938 5.96889333 10.20667543
H 1.31022195 8.84196853 4.68631975
H 11.23932772 1.70010438 8.68754728

H 12.39391001 3.66713260 2.02497339
H 0.18952703 6.92252770 11.33402389
H 0.48123564 9.99662182 3.62317675
H 12.07387328 0.55880670 9.76101379
H 13.21141445 4.82659222 3.08928669
H -0.62302147 5.76298576 10.26342618
H 2.55321484 3.97668585 12.38459250
H 10.01361447 6.56811000 0.99709984
H 8.90643086 9.21958789 7.65935641
H 3.44203841 1.27500480 5.71075067
H 0.86300777 3.88958534 12.89735208
H 11.70001089 6.66106218 0.47528570
H 10.59195249 9.11887439 7.13479165
H 1.82727514 1.55430770 6.32971902
H 1.79406010 2.39368704 12.67066098
H 10.76494808 8.15383641 0.70332241
H 9.64825808 7.62834955 7.36230245
H 2.89679790 2.93658394 6.01218178
H 5.96529236 1.49417229 0.94745149
H 6.60685349 9.05086727 12.45341068
H 7.74359946 6.76072558 5.75715737
H 4.80743466 3.78822859 7.56873302
H 6.51788880 2.34063678 2.40941842
H 6.05035499 8.22520173 10.98079124
H 7.16601434 7.62815336 4.31862760
H 5.34754357 2.96544202 9.04728728
H 6.89358270 2.99419950 0.81611674
H 5.65899703 7.56158326 12.56893035
H 6.78124035 8.23588274 5.93057320
H 5.76120382 2.30058397 7.47309732
H 3.38656416 1.38921101 1.69531522
H 9.16271957 9.14824808 11.67240002
H 10.27074803 6.68597415 5.00006307
H 2.25680314 3.89839341 8.36908963
H 2.45685123 2.77960861 2.28904735
H 10.08400810 7.76483273 11.05706984
H 11.18920540 8.07655308 4.38649225
H 1.32853934 2.51940699 8.98969883
H 3.78487905 2.13577063 3.25841272
H 8.74270290 8.41334837 10.10942770
H 9.82898749 7.44070167 3.45135208
H 2.68354789 3.16131014 9.92885665
C 4.75289499 8.64694602 2.24390847
C 7.79664190 1.88859735 11.13934146
C 8.96392447 3.45201029 4.49176251

C 3.64393350 7.09763095 8.88805004
C 4.60810779 8.18297098 0.81061815
C 7.94796676 2.34953891 12.57425437
C 9.11343951 2.99958711 5.93100456
C 3.50086686 7.55960651 7.45255907
C 1.50992930 5.23320014 1.60351003
C 11.03446892 5.35906008 11.72364301
C 12.20312667 10.46142543 5.13549005
C 0.37887419 0.08533548 8.23852449
C 2.05044469 5.66479248 4.02138264
C 10.49975000 4.87285711 9.34958940
C 11.64136586 0.46090405 2.70289781
C 0.93860292 10.08013562 10.67090690
C 6.53342001 5.65206568 1.97033770
C 5.99545829 4.89768447 11.42500950
C 7.13033149 0.49957047 4.91043434
C 5.44832052 10.11091372 8.62761478
C 5.07267271 4.61225463 3.90661527
C 7.47102543 5.89551988 9.47962629
C 8.48633936 9.95908141 2.92521847
C 3.93880103 0.63376935 10.54185640
C 0.90492266 8.92848429 0.90291137
C 11.64943201 1.63198632 12.47455315
C 12.79728618 3.75603139 5.80700763
C -0.20065301 6.82963906 7.54767654
C 14.10784863 7.52708589 2.78370301
C -1.56326218 3.03796703 10.59433768
C -0.39708442 2.26150106 3.92170722
C 13.00202075 8.33768892 9.42638677
C 1.29827900 9.28076185 3.69091010
C 11.25415526 1.27120740 9.68754149
C 12.40138753 4.10245852 3.02213078
C 0.18918887 6.48398310 10.33824346
C 1.84297474 3.43104359 12.99949886
C 10.71784555 7.11565068 0.37680570
C 9.60795050 8.66756859 7.04023734
C 2.85353331 1.90942886 6.36920768
C 6.13776822 2.47032974 1.39730100
C 6.42148713 8.08148071 11.99372403
C 7.54313124 7.74266686 5.33311032
C 4.98345842 2.81923912 8.03161965
C 3.44398989 2.32877525 2.24327658
C 9.09667262 8.21239668 11.11869552
C 10.20287572 7.62807693 4.45651999
C 2.31800208 2.96255387 8.92333909

O 0.84960648 4.49125881 1.00734856
O 11.71146314 6.13667479 12.25375808
O 12.83338194 9.68286766 5.72322756
O -0.27271379 0.85521644 7.66790133
O 1.74354406 5.19942001 5.04017783
O 10.82805612 5.33392346 8.33683065
O 11.97816035 10.47032106 1.70939796
O 0.58321219 0.06433064 11.65933296
O 7.64925179 5.79816351 1.70430121
O 4.87245192 4.76462876 11.67502070
O 5.91696404 0.08506400 4.65483119
O 6.56978231 9.98768300 8.36752179
O 5.24027954 4.05488314 4.90948965
O 7.31189567 6.41047452 8.45510259
O 8.27282112 9.42019083 1.91869157
O 4.10237260 1.15529271 11.56099433
P 1.08568254 7.97454094 2.44381282
P 11.45947287 2.58064745 10.93181067
P 12.61728090 2.79283370 4.26881759
P -0.02145864 7.78537093 9.08627355
P 4.61723513 3.46409346 1.42750603
P 7.93560033 7.07757687 11.95260971
P 9.05261178 8.75454179 5.31477536
P 3.46453666 1.82143898 8.08368604
S 4.28787159 7.33894211 3.43422052
S 8.24656710 3.20488436 9.95259258
S 9.40369108 2.13086206 3.30901906
S 3.18168621 8.41054779 10.07520494
S 3.86093575 6.51675284 0.71038904
S 8.69100556 4.01895944 12.67610416
S 9.90247848 1.35790268 6.06216764
S 2.76667720 9.23346919 7.35242254
Fe 2.51162712 6.32341873 2.49445181
Fe 10.02773469 4.23060844 10.88122259
Fe 11.19742560 1.12314911 4.23317788
Fe 1.41159750 9.43825012 9.13813423
Fe 4.86730903 5.43634770 2.39622835
Fe 7.66524331 5.09842111 11.00420678
Fe 8.74138532 0.27897226 4.38708908
Fe 3.77631609 10.30941454 9.04236880
H 5.28364563 0.44756133 5.29024217

5.2.7 S *trans* to P

177

Converted from jawg015j-out.cell by c2x
H 4.05963093 9.53750095 2.47619970
H 8.41933552 1.00054822 10.99152470
H 9.53228571 4.22491971 4.26764856
H 2.92892534 6.05244288 8.66532361
H 5.71865146 8.95982441 2.50333500
H 6.74926708 1.54817230 10.96057331
H 7.86958923 3.65739489 4.24904297
H 4.66404702 6.66850131 8.74586343
H 5.53514714 8.12096928 0.35134750
H 6.92365953 2.42080568 13.10856259
H 8.06645150 2.80580431 6.40454918
H 4.32227988 7.86718803 6.61332524
H 3.95187367 8.88903101 0.22988140
H 8.50129239 1.65945958 13.23768994
H 9.64327485 3.56865353 6.51483502
H 2.70096134 7.18781141 6.45464049
H 0.08227824 9.70623774 1.00041094
H 12.37873124 0.85064969 12.44121203
H 13.51410861 4.37379882 5.72701923
H -0.99023818 6.20040973 7.67930973
H 0.64520718 8.29337093 0.08517506
H 11.81581441 2.27055486 13.34846993
H 12.96057090 2.95140539 6.63499680
H -0.44256460 7.67009678 6.84331853
H 1.81003612 9.48094486 0.67587217
H 10.65020519 1.07466342 12.77548355
H 11.78874667 4.14337998 6.06647645
H 0.73981324 6.48242506 7.39760027
H 13.95961916 7.03508773 3.71345559
H -1.50994270 3.50954555 9.71928238
H -0.37884683 1.76492413 2.99443892
H 12.79548075 8.73995239 10.47261918
H 13.74507649 6.76207850 1.97659221
H -1.29289624 3.77817815 11.45733813
H -0.15676531 1.48835174 4.72930208
H 12.60989333 9.02681254 8.73712605
H 13.38117332 8.35246012 2.67489923
H -0.92998873 2.18995753 10.75596671
H 0.20507509 3.07973220 4.03355873
H 12.24811289 7.42797158 9.40870545
H 2.17829488 9.83079504 3.57068403
H 10.29001300 0.69017682 9.87860018
H 11.42216498 4.54083993 3.18293882
H 1.11695523 6.07328286 10.31712803

H 1.24780610 8.86208325 4.70084460
H 11.20971512 1.65786718 8.73978435
H 12.33200297 3.57779266 2.03271918
H 0.06437254 6.91892686 11.43645353
H 0.41613870 10.01646437 3.64538437
H 12.05482520 0.52093243 9.80634704
H 13.18447441 4.70805963 3.10129882
H -0.63305554 5.75689220 10.29549567
H 2.48614048 3.98496222 12.54015387
H 9.98239006 6.55356613 0.89521812
H 8.84445169 9.25614699 7.60909305
H 3.63658659 1.17730640 5.84133010
H 0.79536926 3.90493027 13.04706861
H 11.66596263 6.65949709 0.37323722
H 10.52990369 9.16012726 7.08726040
H 1.94160081 1.26878174 6.32836279
H 1.71454949 2.40412832 12.79671687
H 10.72595015 8.14788174 0.62823739
H 9.59564667 7.66747828 7.33700703
H 2.86292002 2.76907577 6.04890408
H 5.91728193 1.52985060 0.94085505
H 6.51788657 9.00363738 12.48777086
H 7.65412198 6.79248795 5.78378843
H 4.75682196 3.72957292 7.62367847
H 6.47369295 2.34936467 2.41351362
H 5.94458642 8.15721219 11.03272620
H 7.09833521 7.60907269 4.30805397
H 5.32805624 2.92959843 9.10146225
H 6.86114197 3.01951278 0.82622391
H 5.59074380 7.50283217 12.63479249
H 6.71756456 8.28944018 5.89259814
H 5.70904791 2.23577612 7.52409426
H 3.37546957 1.40506544 1.69671890
H 9.06107805 9.13502193 11.72342799
H 10.19645626 6.66547232 5.00648762
H 2.21605266 3.84036585 8.31589927
H 2.43147005 2.77046371 2.32316554
H 10.00198117 7.76251130 11.10732446
H 11.13967258 8.03582221 4.39124396
H 1.27431698 2.48871573 8.98507900
H 3.78011831 2.12624395 3.26798435
H 8.65928680 8.40428281 10.15508895
H 9.79892373 7.39254730 3.43582330
H 2.60355276 3.18719207 9.92170441
C 4.69347355 8.67896337 2.26413813

C 7.76938854 1.84890137 11.19658572
C 8.89462843 3.36928336 4.48060507
C 3.70488908 6.67771071 8.25455414
C 4.56094481 8.21282543 0.82916835
C 7.89594659 2.32837995 12.62826129
C 9.03353206 2.89648495 5.91354534
C 3.42694268 7.58374883 7.16195864
C 1.46153108 5.21480918 1.64294663
C 10.98867393 5.32394260 11.77122046
C 12.11057257 10.46768073 5.04131456
C 0.34882869 0.02887773 8.35524394
C 1.97354672 5.68748104 4.02668256
C 10.46291896 4.83073080 9.38680098
C 11.59223138 0.38667182 2.67396738
C 0.73023839 10.24878408 10.77580266
C 6.47968084 5.67614801 1.95823430
C 5.97174910 4.85960370 11.45959082
C 7.10654218 0.37399515 4.75894910
C 5.33286453 10.16033889 8.71520121
C 5.03534813 4.66560682 3.91651914
C 7.47360681 5.86496126 9.50763476
C 8.53799789 9.93704887 2.78480501
C 3.95614442 0.74922719 10.66079256
C 0.87933977 8.96852334 0.90596909
C 11.57934191 1.58588657 12.53580802
C 12.71836774 3.63457840 5.82302741
C -0.20852303 6.95902323 7.63355683
C 14.04438108 7.48979853 2.72784911
C -1.59265320 3.05258957 10.70414295
C -0.45910853 2.21794207 3.98130363
C 12.90435663 8.29401401 9.48565053
C 1.23715107 9.30406750 3.70636419
C 11.22626434 1.22434680 9.73769915
C 12.35578092 4.00501997 3.03255965
C 0.13182775 6.52013447 10.42622487
C 1.76993491 3.43470846 13.14449219
C 10.68369789 7.11539930 0.28415457
C 9.54934717 8.70092989 6.99588063
C 2.91305262 1.74615220 6.41987005
C 6.09989782 2.49887081 1.40242567
C 6.33530861 8.02793212 12.04037479
C 7.47405793 7.75994064 5.31822357
C 4.94991860 2.76674505 8.09419377
C 3.42484349 2.33592025 2.26050689
C 9.01017532 8.20121936 11.16499230

C 10.14744572 7.59833442 4.44599420
C 2.26227073 2.93459943 8.91944630
O 0.80446631 4.43190025 1.09591401
O 11.64854443 6.10695800 12.31502037
O 12.76980777 9.68219040 5.58229786
O -0.30063730 0.74937735 7.72188763
O 1.64182351 5.21805892 5.03442950
O 10.78077496 5.29113964 8.36970034
O 11.88556435 10.49982114 1.63677779
O 0.36972346 0.15446645 11.78162136
O 7.59507309 5.80417717 1.67840646
O 4.85567459 4.72796260 11.73289333
O 5.98878880 0.51203730 5.02604737
O 6.42693147 9.99791605 8.37823440
O 5.21631580 4.13568862 4.92817795
O 7.35629741 6.39323643 8.48583348
O 8.36296022 9.42151521 1.76349879
O 4.15519444 1.32858082 11.64426471
P 1.03398410 8.00694636 2.44601935
P 11.41849717 2.53318223 10.98807763
P 12.55391286 2.68921969 4.27405187
P -0.09458711 7.85596100 9.21505000
P 4.57754591 3.49127426 1.44379263
P 7.86404146 7.04452741 11.98813962
P 8.99592203 8.75161354 5.26624455
P 3.43600138 1.76669285 8.15789794
S 4.22807316 7.36090253 3.44351598
S 8.21590049 3.15994325 10.00023486
S 9.35141904 2.05860532 3.28717430
S 3.05575417 8.59691575 10.38310002
S 3.80347336 6.55063147 0.71841927
S 8.65465776 3.99157901 12.71825047
S 9.79233767 1.23319809 6.00569382
S 2.66926583 9.29969516 7.53787385
Fe 2.45282239 6.34410610 2.50190079
Fe 9.99626804 4.18939923 10.92193905
Fe 11.13221261 1.03059873 4.21061675
Fe 1.29609396 9.54753433 9.30705041
Fe 4.81745709 5.47134463 2.39664364
Fe 7.63910470 5.06241584 11.03532561
Fe 8.76916633 0.16045326 4.32655500
Fe 3.68239683 10.41553559 9.18973316
H 2.97859646 9.05406035 11.65078092

5.2.8 S *cis* to P

```
177
Converted from jawg015k-out.cell by c2x
H 4.06819225 9.52037855 2.41428886
H 8.38780562 0.96441028 10.93611603
H 9.38037073 4.40056898 3.90572832
H 2.95521000 6.23891134 9.08277543
H 5.72675807 8.94436031 2.43746595
H 6.72920810 1.53914340 10.91072954
H 7.73779845 3.75563150 3.85426447
H 4.61503193 6.81281490 9.09581700
H 5.52710637 8.10879063 0.27234539
H 6.92636148 2.38258877 13.07273745
H 7.77497122 3.30524280 6.28748962
H 4.40829554 7.63951265 6.93013492
H 3.94291837 8.85520100 0.17261562
H 8.50723585 1.63045519 13.17594921
H 9.43160957 4.11311331 6.36647474
H 2.82339738 6.89286727 6.83786422
H 0.06383561 9.66230511 0.95836645
H 12.40338402 0.82791713 12.38580395
H 13.49603905 4.40981122 5.71468101
H -1.06552111 6.09634266 7.63578466
H 0.63344704 8.24666311 0.04927582
H 11.82934412 2.23763563 13.30043427
H 12.97614293 2.97014329 6.61696959
H -0.49062917 7.50483204 6.71977826
H 1.79287157 9.45087454 0.62309879
H 10.67320372 1.03196507 12.72735301
H 11.77149441 4.11891333 6.03859010
H 0.66320102 6.29662168 7.28949764
H 13.93980034 7.00746994 3.71329794
H -1.48493216 3.48543884 9.63545324
H -0.29581818 1.78285668 2.94454265
H 12.83237175 8.73605479 10.38474380
H 13.71469574 6.76156586 1.97259741
H -1.24951181 3.73217664 11.37479516
H -0.04826463 1.55756568 4.68254341
H 12.60623384 8.98147657 8.64583819
H 13.35696191 8.33893426 2.69820493
H -0.89789994 2.15364092 10.64988230
H 0.26228342 3.13715468 3.94677022
H 12.24331453 7.40407954 9.37011239
H 2.20931338 9.80592184 3.51706712
```

H 10.25705456 0.70108011 9.80866734
H 11.38625571 4.49924022 3.15998232
H 1.08421270 5.95109894 10.19254823
H 1.24792411 8.87052375 4.64876587
H 11.22175073 1.67402436 8.70516533
H 12.33367221 3.56548496 2.01404535
H 0.13759183 6.90485689 11.32114695
H 0.45018906 10.02647022 3.56716164
H 12.01898069 0.48421792 9.75638321
H 13.14155283 4.73210438 3.07875923
H -0.67702512 5.74911873 10.24970758
H 2.50835200 3.91771982 12.40846840
H 9.95312550 6.56139618 0.93046396
H 8.92900829 9.00721422 7.66546571
H 3.62315078 1.26216833 5.74503350
H 0.81749443 3.81619562 12.91661440
H 11.64358456 6.66296449 0.42237894
H 10.58297750 8.61787934 7.13416606
H 1.93456484 1.36189873 6.26182024
H 1.76144834 2.32788544 12.68832533
H 10.69928245 8.15188511 0.65392198
H 9.38152519 7.33345896 7.29953143
H 2.87462713 2.85098942 6.02140786
H 5.91552206 1.50117818 0.93107376
H 6.54556833 8.98302227 12.41737544
H 7.60220993 6.63990804 5.60060842
H 4.80143664 3.72915160 7.60604087
H 6.46065676 2.32988524 2.40525005
H 5.99589062 8.15435380 10.94422667
H 7.10870870 7.60347308 4.19641821
H 5.35265131 2.89403674 9.07359341
H 6.85573826 2.99456210 0.81750830
H 5.60568850 7.48687036 12.53037708
H 6.71078806 8.15354001 5.82660464
H 5.74560506 2.23805786 7.48295764
H 3.35128118 1.39324525 1.69494673
H 9.10856261 9.08913944 11.65005388
H 10.21264139 6.51688737 4.95076448
H 2.24543700 3.83122137 8.36017960
H 2.42295599 2.77782256 2.30270647
H 10.03396736 7.70715656 11.03370873
H 11.15107247 7.90582722 4.36532869
H 1.31464100 2.45313604 8.97689199
H 3.76360045 2.13383970 3.25685436
H 8.68982359 8.35550015 10.08681008

H 9.79310924 7.29350896 3.40958046
H 2.65959623 3.09941073 9.92504655
C 4.70121703 8.66096980 2.20321299
C 7.75423477 1.82434165 11.14477426
C 8.71270421 3.65418454 4.32734832
C 3.58903420 7.09589884 8.86515976
C 4.56238552 8.18898638 0.76986276
C 7.89208533 2.29833951 12.57723881
C 8.62833430 3.71231881 5.76821446
C 3.44508445 7.56104867 7.43034656
C 1.47001368 5.21569674 1.58367485
C 10.99109031 5.29752036 11.71620802
C 12.23330557 10.40046182 4.92000037
C 0.35831754 0.02428889 8.30556542
C 2.00305285 5.67253876 3.99395764
C 10.46549926 4.80906892 9.34897280
C 11.52999048 0.46463830 2.60848861
C 0.89943524 10.08289337 10.65609593
C 6.48984063 5.65265360 1.94012413
C 5.95860110 4.83066126 11.40710887
C 7.16724928 0.33428244 4.87211474
C 5.38675174 10.10477401 8.58282018
C 5.00457160 4.65523890 3.89599224
C 7.41458932 5.82587902 9.45406087
C 8.63303069 9.90509266 2.89934885
C 3.92831038 0.57312693 10.55975722
C 0.86776251 8.93180711 0.86165540
C 11.59587576 1.55402628 12.48653071
C 12.72234968 3.64692327 5.80372945
C -0.25719909 6.82140409 7.53396268
C 14.02042866 7.47599083 2.73420860
C -1.56030204 3.01757665 10.61561834
C -0.38031389 2.25824214 3.92014667
C 12.90944392 8.26486932 9.40658658
C 1.25604929 9.29936885 3.64900074
C 11.21065376 1.21065882 9.68943317
C 12.33965172 3.99885649 3.01203510
C 0.13680159 6.46827699 10.32452177
C 1.80055510 3.36458390 13.01981721
C 10.66100432 7.11583217 0.32048440
C 9.52890203 8.37380948 7.01700276
C 2.91731378 1.81611578 6.35788285
C 6.09334638 2.47167813 1.39055374
C 6.36639177 8.01246617 11.95781099
C 7.46451657 7.65295018 5.22357802

C 4.98253074 2.75618356 8.05916305
C 3.41127428 2.33092078 2.24598159
C 9.04609294 8.15383664 11.09523338
C 10.15877773 7.46660836 4.42015432
C 2.30419079 2.89640454 8.91635382
O 0.82154438 4.45487000 0.99698418
O 11.65756340 6.08616044 12.24322488
O 12.90635617 9.62205710 5.45488619
O -0.31088046 0.80272066 7.76642373
O 1.69555655 5.20444250 5.01106366
O 10.80034162 5.28508427 8.34481619
O 11.76424756 10.58120961 1.52348604
O 0.59842546 0.02077175 11.70081149
O 7.60773906 5.78721363 1.67021370
O 4.83888192 4.69940374 11.67158915
O 6.03321657 0.49642732 5.02494602
O 6.50695345 9.97069305 8.32320924
O 5.14210186 4.13546593 4.91967912
O 7.23823546 6.34788701 8.43637965
O 8.46451631 9.40314481 1.87004807
O 4.09334386 1.09187310 11.58090150
P 1.04221586 7.98557468 2.40829648
P 11.41856175 2.50965768 10.94665253
P 12.58795852 2.69776343 4.25749770
P -0.07047573 7.77451198 9.07539093
P 4.57317580 3.46737958 1.41754618
P 7.88514735 7.01556048 11.92381412
P 9.01546991 8.59429250 5.28474917
P 3.46562971 1.75758743 8.08921208
S 4.23807463 7.35295132 3.39596308
S 8.21460678 3.13065690 9.94917664
S 9.32696244 2.04880561 3.44381699
S 3.13581727 8.41080929 10.05252398
S 3.81321366 6.52170280 0.67567724
S 8.64428363 3.96420479 12.66939059
S 10.01205826 1.11776310 6.13046768
S 2.69486812 9.22733356 7.33005756
Fe 2.46265392 6.32724660 2.46250405
Fe 9.99129991 4.16120051 10.87900490
Fe 11.19091954 1.01825346 4.20820194
Fe 1.35452051 9.43063984 9.12380437
Fe 4.82247817 5.45079137 2.36402031
Fe 7.62696183 5.03224301 10.98069440
Fe 8.84485306 0.11082330 4.47904381
Fe 3.71641060 10.30596549 9.00703949

H 10.53891331 0.14201379 6.89472801

5.3 Complex 6

5.3.1 Fe−μ−Fe site

161
Converted from jawg016a-out.cell by c2x
H 3.99920615 4.11971271 3.63949108
H -0.71038664 5.40735094 16.15313436
H 4.26452849 4.32943570 5.37080453
H -0.80973756 5.28474334 14.39466984
H 4.71073943 2.06811746 5.51908060
H -1.33734229 7.52873708 14.40074251
H 4.25649007 1.83477712 3.82775542
H -0.84340466 7.70436023 16.09029982
H 0.47988394 2.48729219 13.63520948
H 2.83265872 7.05807832 6.16974707
H -0.38628234 3.58569665 12.57568637
H 3.68152453 5.93693355 7.21771608
H -1.76752031 2.94189521 14.50169989
H 5.06455101 6.61029642 5.28464900
H -1.65916900 1.23476430 14.09827608
H 4.99604017 8.30364397 5.75359989
H 5.14372306 2.26423377 12.98996838
H -1.82843132 7.21701495 6.81381737
H 0.67641960 0.28157342 13.17377079
H 2.63332781 9.24141412 6.64513857
H -0.91768238 -0.08564874 12.49010050
H 4.23062059 9.61165299 7.31575158
H -0.68802798 8.39818831 11.71671411
H 4.02354000 1.12608624 8.11496726
H 0.53154225 9.56225335 11.25058219
H 2.79089581 -0.02747246 8.56974694
H -0.99397082 9.46915235 10.35242424
H 4.31549625 0.04004476 9.47029469
H -0.85226735 1.09436447 9.93571954
H 4.18470433 8.40039720 9.87825185
H 5.64001826 1.14885104 11.19194703
H -2.32008024 8.39318324 8.60471241
H 5.33328492 2.88812419 9.47575819
H -1.98910377 6.62200641 10.30243285
H -0.71290882 3.58379338 9.73433314
H 4.08015907 5.88683048 9.99000589
H -1.90608576 3.76113876 11.02830629

H 5.30053056 5.78911126 8.71557497
H 1.02351366 3.20714765 10.83939261
H 2.30632957 6.32123530 8.99336019
H 1.32864861 1.59370271 10.21867761
H 2.00550292 7.93965842 9.60245004
H 3.29287053 2.42698096 11.37192742
H 0.03163190 7.10258393 8.47404070
H 2.67973163 1.09828786 12.34035903
H 0.63876560 8.41384494 7.48111318
H 2.41433617 2.78564713 12.83968891
H 0.89865854 6.71815566 7.00356649
H 0.98303441 6.32741442 3.02094800
H 2.33168109 3.27353537 17.01544646
H 0.12685499 5.87397686 1.56266513
H 3.13041694 3.72329540 18.50780551
H 2.17245503 4.60393444 1.71094306
H 1.06223630 4.96570493 18.28483835
H 3.17072183 6.05167790 1.83799875
H 0.09981822 3.49884743 18.13608691
H 2.31808809 5.68952579 0.33959330
H 0.90468632 3.87769559 19.65550661
H 2.94532824 8.24659782 1.34857915
H 0.32389597 1.34107026 18.62117540
H 1.91185092 9.63375757 1.71087969
H 1.36584236 -0.04797870 18.29911036
H 3.31806658 9.11481859 3.63599415
H -0.00347860 0.48078498 16.34312339
H 2.72803193 7.46491671 3.72361973
H 0.64632053 2.10845409 16.23551106
H 1.65858700 8.83704394 4.08597045
H 1.67436161 0.69158485 15.92756690
H -1.26491011 7.20051492 19.52224972
H 4.50449461 2.33122068 0.49048998
H -2.93044533 6.62159700 19.58461303
H 6.15923475 2.95293960 0.46907615
H -2.42057214 8.78495361 18.29037535
H 5.76309630 0.78525746 1.73422359
H -2.27666730 9.60378375 19.83764151
H 5.53784515 -0.03840645 0.19460924
H -3.80670807 8.85223187 19.36360761
H 7.08977239 0.72148430 0.59335418
H -0.17997583 8.30843546 3.24527626
H 3.46645260 1.29182694 16.78644239
H -0.30948684 9.45923878 1.93176174
H 3.59736988 0.13857721 18.09573031

H 5.23748548 8.25258995 2.17974602
H -1.95561287 1.36524934 17.86049998
H 5.93997509 6.64779999 2.09984257
H -2.67617047 2.96234568 17.94555052
H 5.85837703 7.66625241 0.64873236
H -2.58212761 1.93559392 19.39142683
C 0.45774705 1.36313917 7.31075364
C 2.85494740 8.12669794 12.44273783
C -0.23566504 3.76333038 6.70490072
C 3.46454131 5.64432288 12.94373015
C 2.15924027 3.37924530 7.81878206
C 1.04790596 6.17090964 12.07996376
C 1.72075073 2.08222511 2.38019514
C 1.44737901 7.50452665 17.57981260
C -0.39329544 3.45646963 3.47306867
C 3.73480476 6.25346312 16.72552904
C -0.16996716 1.02133673 4.11776934
C 3.47511575 8.67077297 16.05251638
C 3.70708699 3.76794271 4.62483473
C -0.31715380 5.80067988 15.21770485
C 3.94667713 2.27923431 4.77222424
C -0.55149338 7.30245624 15.12171088
C -0.36949822 2.56955443 12.96303120
C 3.67948033 6.95816098 6.84265025
C -1.65585818 2.23678505 13.67907340
C 4.97119396 7.28703952 6.13317125
C 0.09111878 0.24788668 12.25692714
C 3.22440261 9.27567816 7.55799889
C -0.54161217 9.40896800 11.34003703
C 3.86620134 0.11362837 8.48222134
C -1.18000524 1.72260536 10.76121830
C 4.51342142 7.79622967 9.03530801
C -1.53516871 3.09755695 10.25118375
C 4.89643610 6.41564965 9.50813940
C 1.21772393 2.17934488 11.12773239
C 2.10962876 7.34646025 8.69786612
C 2.45608857 2.10482590 11.99349555
C 0.86434196 7.41193626 7.84026730
C 1.02328512 6.35685642 1.93544037
C 2.25324518 3.23374153 18.09868490
C 2.24907270 5.65109523 1.42164011
C 1.00147854 3.91793882 18.57547207
C 2.12110508 8.59749732 1.97076987
C 1.16590395 0.98657211 18.02536439
C 2.45629520 8.47986792 3.43933126

C 0.87442224 1.09022152 16.54658166
C -2.26367586 7.42313801 19.88748343
C 5.51616527 2.14042841 0.13973499
C -2.72410273 8.74883026 19.33409647
C 6.00950012 0.81762336 0.67440166
C -0.32820885 8.39924716 2.17389086
C 3.61213888 1.19905289 17.85751060
C 6.03218610 7.66253422 1.72240204
C -2.75767576 1.94498465 18.31846134
N 0.01516873 0.42274934 7.84628963
N 3.28341556 9.06156941 11.88792676
N 2.27052884 1.85967196 1.36947854
N 0.82545519 7.72391434 18.54563609
N -0.05515105 1.68202206 11.78173066
N 3.37558644 7.83930347 8.03009529
N 0.90611448 7.80660967 1.52697698
N 2.36873063 1.78119954 18.49784859
O -1.21684387 4.35008742 6.88405101
O 4.37322941 4.97744514 12.68296700
O 2.75270698 3.78541697 8.73118491
O 0.55244030 5.76448315 11.11170190
O 6.47045482 4.12008655 3.05376160
O -3.13825148 5.65629258 17.26793196
O 6.79377046 0.01543820 4.14866165
O -3.52523358 9.70534756 16.09400513
S 2.43319685 1.39177417 5.27820872
S 0.93731982 8.21555143 14.60973946
S 1.94480313 4.20178306 4.82839886
S 1.44529108 5.35051122 15.12761931
Fe 1.22360286 2.85738721 6.46431211
Fe 2.14405323 6.66230574 13.37822227
Fe 0.85651687 2.40087155 4.01965184
Fe 2.52064870 7.23942144 15.99373366
H 3.27216309 7.07178934 14.54417923

5.3.2 $C{\equiv}O_{basal}$ geminal to CN

161
Converted from jawg016b-out.cell by c2x
H 4.04993223 4.09018622 3.52125542
H -0.75795466 5.29030615 15.83322030
H 4.27512603 4.27278612 5.26044578
H -0.85692475 5.18948214 14.07025488
H 4.74734433 2.01334855 5.36295735
H -1.31580311 7.43928660 14.10836281

H 4.28394984 1.80078528 3.67201253
H -0.78507987 7.60169656 15.79188232
H 0.48324455 2.38927334 13.45630752
H 2.94826872 6.95644144 5.99065573
H -0.34981702 3.50871902 12.39366031
H 3.80316183 5.85191778 7.05077732
H -1.75305205 2.87508484 14.30983525
H 5.20070984 6.52081766 5.13574974
H -1.69014605 1.17047321 13.87704549
H 5.09839078 8.22138458 5.57660719
H 5.23098260 2.26220781 12.80918762
H -1.79970547 7.14005680 6.63849466
H 0.71574557 0.20582107 12.97713582
H 2.69730347 9.14206362 6.47398770
H -0.89340747 -0.16781443 12.33373786
H 4.29711722 9.52916780 7.13008260
H -0.68452024 8.22585548 11.45303293
H 4.04554443 1.11040372 8.01132011
H 0.54610686 9.37458721 10.97001168
H 2.84107259 -0.06451102 8.48290905
H -0.99224406 9.30438962 10.09728620
H 4.37572239 0.02922067 9.36235734
H -0.84635633 1.03527256 9.74420085
H 4.25523918 8.30488036 9.71288504
H 5.73104130 1.07621661 11.03895982
H -2.31888111 8.29630563 8.41837581
H 5.40258398 2.83902866 9.34110123
H -1.98285840 6.52437638 10.11672587
H -0.73779125 3.53848783 9.60158257
H 4.18029471 5.80057783 9.82183369
H -1.94057385 3.66863337 10.88851213
H 5.40522603 5.71178708 8.54623733
H 1.03124163 3.13111637 10.65728658
H 2.40614924 6.21583839 8.79900021
H 1.31046339 1.52419607 10.00627520
H 2.09028962 7.82573157 9.42164161
H 3.29110878 2.37513102 11.14589722
H 0.13241541 6.96119628 8.28667880
H 2.72834297 0.97894694 12.05014980
H 0.70654607 8.31127618 7.32646897
H 2.43311784 2.62373712 12.65215023
H 0.99993075 6.63607435 6.80227947
H 1.03267263 6.25716272 2.89732235
H 2.40225556 3.10803390 16.56444332
H 0.19671028 5.78112853 1.43411455

H 3.23647247 3.58162501 18.03018521
H 2.25473803 4.54349852 1.58146591
H 1.17809042 4.81909951 17.88691076
H 3.23303455 6.00107070 1.73793641
H 0.20237187 3.36341878 17.70648317
H 2.40418650 5.64816745 0.22512415
H 1.01693382 3.69880561 19.23135580
H 2.98587019 8.19069865 1.19422613
H 0.44732007 1.17480588 18.26069730
H 1.94351440 9.56502558 1.57193990
H 1.48895617 -0.20170913 17.88985258
H 3.37662711 9.05333022 3.47535539
H 0.05766766 0.30728824 15.98337961
H 2.81192923 7.39423941 3.56319001
H 0.63407633 1.96302998 15.88623383
H 1.72949955 8.74830749 3.95249798
H 1.70695590 0.59737735 15.50690589
H -1.19534020 6.99322623 18.97379554
H 4.63135583 2.36765734 0.48616407
H -2.85749502 6.39322101 19.04109725
H 6.29307886 2.96919044 0.41623895
H -2.44515726 8.53767328 17.72800890
H 5.88356735 0.82165336 1.72856031
H -2.20038940 9.38111226 19.25217924
H 5.64096419 -0.01870029 0.20259416
H -3.75897305 8.63002801 18.88014862
H 7.19797979 0.73595128 0.57731659
H -0.12543551 8.23619048 3.11641700
H 3.55461986 1.12555614 16.33937765
H -0.27663857 9.37430231 1.79237113
H 3.70777586 -0.01303301 17.66253733
H 5.36496708 8.15779948 2.11843373
H -1.93208953 1.19927391 17.33168455
H 6.08939382 6.56753122 1.99299242
H -2.65403555 2.79135595 17.45756184
H 5.95616346 7.61477675 0.56454196
H -2.51800561 1.74470537 18.88582325
C 0.47254469 1.30421126 7.14177521
C 2.96586378 7.94680761 12.30277666
C -0.22422201 3.69752555 6.53428818
C 3.86310967 5.68469190 12.95733980
C 2.18256778 3.31777444 7.64720094
C 1.28486838 5.97760169 11.73599582
C 1.77284703 2.05435473 2.22493014
C 1.65774285 7.30113385 17.20155214

C -0.38187463 3.39427768 3.33711167
C 3.80688887 5.92504328 16.11305432
C -0.16186784 0.97888788 3.98610883
C 3.60798571 8.31631505 15.44278103
C 3.73545166 3.72138964 4.49372725
C -0.35829860 5.67862471 14.90221875
C 3.97739845 2.23128241 4.62388746
C -0.53233326 7.18232047 14.81946690
C -0.35558887 2.49007854 12.77445475
C 3.79096779 6.87046584 6.67017726
C -1.65454011 2.17936669 13.47733722
C 5.08415168 7.21037860 5.97077405
C 0.10895080 0.16690981 12.07384922
C 3.29725892 9.18126959 7.38133515
C -0.52730886 9.23484383 11.07753914
C 3.91235063 0.09607923 8.38098038
C -1.17830480 1.64334219 10.58336898
C 4.59789121 7.71235643 8.86752326
C -1.55370214 3.02389524 10.10109765
C 4.99296526 6.33421947 9.33768664
C 1.21970915 2.09694515 10.92588359
C 2.20210397 7.24140274 8.51212969
C 2.47505260 1.99729424 11.76425391
C 0.95491925 7.30531249 7.65810827
C 1.08201267 6.28135009 1.81160725
C 2.35201518 3.08179341 17.65013248
C 2.32124675 5.59485139 1.30589517
C 1.10950384 3.76518243 18.15214243
C 2.16538734 8.53000327 1.82690349
C 1.26861847 0.83253384 17.63092440
C 2.52073625 8.40689308 3.28972197
C 0.91724772 0.94975509 16.16634844
C -2.19697961 7.20336668 19.34053257
C 5.63289134 2.15841497 0.11786111
C -2.68083718 8.52164234 18.78975131
C 6.11943329 0.84074942 0.66700151
C -0.28384271 8.31652843 2.04516893
C 3.71636899 1.04478064 17.41013904
C 6.15905635 7.59028450 1.63262016
C -2.72426914 1.76885779 17.81843679
N 0.02380382 0.36358023 7.67172672
N 3.40696901 8.89487917 11.78243723
N 2.36409738 1.88274605 1.22809954
N 1.07419193 7.50008943 18.19782993
N -0.04582772 1.60086149 11.59443117

```
N 3.46662273 7.74934315 7.85291602
N 0.95193750 7.72799883 1.39394008
N 2.48217823 1.63419388 18.06432919
O -1.21221213 4.27229263 6.71100721
O 5.00910368 5.80440279 12.62427481
O 2.79943503 3.71306837 8.54856412
O 0.66642437 5.66906639 10.80259045
O 6.53789940 4.07716822 2.98225119
O -3.09679509 5.26686498 16.48172095
O 6.84996619 0.02642308 4.08277765
O -3.39596668 9.26234079 15.35725043
S 2.46530855 1.33791072 5.12408735
S 0.99678837 8.02585210 14.29287118
S 1.96737022 4.14606757 4.67237625
S 1.39392943 5.18386888 14.82933513
Fe 1.24059741 2.79939157 6.29863112
Fe 2.19859829 6.44956675 13.14052443
Fe 0.88469975 2.34363817 3.85840983
Fe 2.54457167 6.96816438 15.57152550
H 3.48881223 4.66265541 13.30539217
```

5.3.3 $C{\equiv}O_{\mathrm{basal}}$ *trans* to CN

```
161
Converted from jawg016d-out.cell by c2x
H 4.03503506 4.10412445 3.54067379
H -0.69601449 5.32693903 15.87075087
H 4.24200831 4.27893382 5.28226150
H -0.89145814 5.16216056 14.12620481
H 4.69756526 2.01408695 5.37778852
H -1.36673155 7.41638827 14.03815017
H 4.23686558 1.80696057 3.68436820
H -0.93121673 7.62565333 15.72703055
H 0.44265181 2.48582629 13.43932256
H 2.86213638 6.98168272 6.01795929
H -0.41994568 3.58347392 12.37983031
H 3.70770501 5.87589126 7.08342618
H -1.80762055 2.92519533 14.29673937
H 5.10961199 6.53633863 5.16438222
H -1.68879297 1.21564577 13.87898373
H 5.01991913 8.23609540 5.61145746
H 5.21367881 2.19433462 12.78659517
H -1.91073659 7.22895644 6.68906640
H 0.69767342 0.30281025 12.96468038
H 2.60032118 9.15444987 6.49241842
```

H -0.89867649 -0.09558179 12.32120528
H 4.20037610 9.55517629 7.14356841
H -0.73599003 8.37897219 11.50876452
H 4.04106675 1.08684055 7.94326646
H 0.44114981 9.56836478 11.00796307
H 2.85844540 -0.09803337 8.44220813
H -1.10306054 9.43744599 10.14858186
H 4.39824335 0.02722002 9.30664130
H -0.86025353 1.11620953 9.72360995
H 4.16648111 8.35360139 9.73137074
H 5.73620573 1.04936542 10.99331344
H -2.43325549 8.41584823 8.45702590
H 5.38321901 2.81130478 9.30289247
H -2.07689219 6.65978180 10.15540870
H -0.79565871 3.62430590 9.59244834
H 4.10235515 5.84599814 9.86848393
H -2.00819459 3.71987803 10.87565611
H 5.31350444 5.74626938 8.58464311
H 0.98223854 3.22061130 10.63352114
H 2.31893676 6.24697010 8.83200990
H 1.29799467 1.60973075 10.01154129
H 2.00732886 7.86042108 9.45042244
H 3.25949204 2.48197915 11.14885159
H 0.04243138 6.98427682 8.31440567
H 2.68342965 1.12468356 12.09988954
H 0.61785087 8.34253423 7.36365321
H 2.38797505 2.79602105 12.63332522
H 0.91232709 6.67246875 6.82937514
H 0.96018518 6.26976497 2.93772076
H 2.31229559 3.25394442 16.57478190
H 0.11875384 5.79722637 1.47545576
H 3.12085246 3.73431284 18.05294082
H 2.18770652 4.56993821 1.61044254
H 1.02663768 4.92138849 17.87391713
H 3.15456507 6.03249630 1.78157495
H 0.09421195 3.43776957 17.69701212
H 2.33316244 5.68473356 0.26229639
H 0.88546108 3.80894555 19.22660453
H 2.88885844 8.21869591 1.22785964
H 0.38495258 1.27442845 18.25083703
H 1.84417315 9.58782003 1.62192757
H 1.44936361 -0.08041463 17.86585111
H 3.30037596 9.07726863 3.50827318
H 0.01186502 0.40399581 15.95600773
H 2.73746139 7.41757475 3.60098371

H 0.54697192 2.07678920 15.87608366
H 1.65700442 8.76920806 3.99911902
H 1.65143322 0.74397663 15.48805838
H -1.36258685 7.14627913 18.98275939
H 4.61541991 2.33141803 0.51703319
H -3.03138199 6.57472121 19.07059221
H 6.27731429 2.93684729 0.44724708
H -2.59459011 8.70643724 17.74514401
H 5.90203520 0.79343903 1.74960057
H -2.32252028 9.55227726 19.26539113
H 5.61372109 -0.04870330 0.23178430
H -3.89689750 8.82839530 18.91527366
H 7.18618681 0.69511286 0.56620707
H -0.20110482 8.24377335 3.17671811
H 3.47455800 1.28846579 16.33040242
H -0.36137760 9.39091102 1.85964782
H 3.66169712 0.15806634 17.66221809
H 5.30298129 8.09640434 2.18281979
H -2.02116595 1.48217340 17.31630717
H 6.02550239 6.50442850 2.03260181
H -2.77271726 3.06225754 17.45461650
H 5.88381840 7.56786791 0.61890836
H -2.60456273 2.00924636 18.87724122
C 0.41467696 1.34863827 7.12517775
C 2.86524801 8.14263059 12.26822368
C -0.27221344 3.75077698 6.53345971
C 3.60496246 5.74011561 12.91004612
C 2.13092362 3.35561002 7.64262357
C 1.19312098 6.12073233 11.81301155
C 1.74709117 2.07096681 2.21849136
C 1.49133333 7.42172644 17.26667233
C -0.42344002 3.42377535 3.34073267
C 3.73717898 6.13072820 16.22307473
C -0.18190424 1.00715135 3.97592221
C 3.68088685 8.50416456 15.35421775
C 3.70584549 3.73469682 4.50791178
C -0.36025053 5.70354579 14.90650980
C 3.93301299 2.24150866 4.63577620
C -0.60457331 7.19646745 14.78221711
C -0.40253366 2.56582617 12.76207874
C 3.70215958 6.89345529 6.70070510
C -1.69076693 2.22320394 13.47143866
C 4.99887576 7.22421805 6.00223689
C 0.09680942 0.25434504 12.05873990
C 3.20215092 9.20673215 7.39779783

C -0.62584392 9.39180661 11.12517189
C 3.92636513 0.07462045 8.32757243
C -1.20425896 1.71471587 10.56446410
C 4.50950294 7.75251786 8.89182830
C -1.60309342 3.09044291 10.08564535
C 4.90930359 6.37824620 9.37308253
C 1.18765495 2.19504655 10.92063146
C 2.11438166 7.27201654 8.54284601
C 2.43601400 2.13226554 11.77329740
C 0.86568916 7.33494930 7.69033379
C 1.00324129 6.29938980 1.85226217
C 2.25077479 3.21958146 17.65943271
C 2.24746320 5.62357129 1.34211736
C 0.98500331 3.86886117 18.14790142
C 2.07412117 8.55304854 1.86987354
C 1.20970089 0.95134795 17.61575671
C 2.44362962 8.42991978 3.32968842
C 0.85334604 1.06927491 16.15155708
C -2.35539889 7.37425812 19.36024486
C 5.61641409 2.12885120 0.14434421
C -2.82162278 8.69929454 18.81038773
C 6.11027876 0.80859754 0.68153500
C -0.36873893 8.33228545 2.10795510
C 3.64906045 1.21299359 17.39974060
C 6.09299778 7.53194152 1.68525697
C -2.82140032 2.03712304 17.81180268
N -0.03008220 0.39788607 7.63911817
N 3.25864006 9.08448556 11.69964687
N 2.34458345 1.87445628 1.22954111
N 0.87141773 7.63894644 18.23338492
N -0.07590357 1.68494097 11.58080073
N 3.37736230 7.77809474 7.87904732
N 0.86102579 7.74803757 1.44413052
N 2.40826147 1.77237563 18.06018569
O -1.25456215 4.33435199 6.71670425
O 4.57751412 5.14049519 12.73398174
O 2.74893530 3.75208658 8.54235207
O 0.56167431 5.72538510 10.92204250
O 6.50783956 4.01616208 2.98150574
O -3.22136578 5.54706848 16.66862882
O 6.86891527 -0.03850882 4.05294532
O -3.11038283 9.06794027 14.84575166
S 2.41084204 1.36392202 5.13308911
S 0.90279782 8.11058326 14.29887675
S 1.93947263 4.17678045 4.66564024

S 1.40899301 5.26845023 14.75135270
Fe 1.18752396 2.84107113 6.29234782
Fe 2.14353928 6.65093071 13.15136452
Fe 0.85170194 2.37801657 3.84935035
Fe 2.46636685 7.13505792 15.62540691
H 3.20370209 9.12717812 16.18288697

5.3.4 *C*≡O$_{\text{basal}}$ *cis* to CN

161
Converted from jawg016f-out.cell by c2x
H 4.03379768 4.12084414 3.58918453
H -0.51935139 5.20293264 16.08149971
H 4.30744733 4.31446436 5.32092421
H -0.76756951 5.07785821 14.33388135
H 4.76725320 2.05159066 5.43719317
H -1.33248219 7.30865858 14.35749699
H 4.28657529 1.83460140 3.75216765
H -0.75979351 7.50231020 16.01911453
H 0.54305607 2.45391707 13.53600474
H 2.89221796 7.04108696 6.10122201
H -0.30309479 3.56787167 12.47793487
H 3.74631074 5.92822014 7.15473868
H -1.70255497 2.90611668 14.39714018
H 5.13789309 6.58929474 5.23247984
H -1.61755900 1.20874612 13.95298249
H 5.04215407 8.29081701 5.66874287
H 5.19393140 2.29590531 12.92319945
H -1.75638313 7.21505157 6.69848167
H 0.75458164 0.26437323 13.06392074
H 2.68122697 9.23181296 6.57347653
H -0.84342699 -0.10430536 12.39179504
H 4.28426278 9.60124545 7.23625317
H -0.60909611 8.38419285 11.53925257
H 4.05164696 1.11438727 8.09669528
H 0.63437871 9.53314858 11.10486102
H 2.82015006 -0.03948531 8.55062015
H -0.88070886 9.47612413 10.18206300
H 4.34862672 0.02508884 9.44978086
H -0.77929859 1.08249490 9.82745817
H 4.22831877 8.38236733 9.81543263
H 5.69393301 1.15001679 11.14100065
H -2.25453080 8.37782104 8.49068894
H 5.38279384 2.89186638 9.42454606
H -1.91859032 6.58634232 10.17531171

H -0.65219975 3.57590933 9.63634301
H 4.14576058 5.85984813 9.89264709
H -1.84946789 3.74086623 10.92731710
H 5.37540530 5.79255046 8.62846621
H 1.09108074 3.18364460 10.72048225
H 2.36206590 6.31475185 8.92488309
H 1.39901333 1.56561580 10.11469593
H 2.05565646 7.93571462 9.52965522
H 3.36268711 2.41124164 11.24862141
H 0.08314445 7.08927690 8.39988059
H 2.76024973 1.09732542 12.24019550
H 0.68937559 8.40126057 7.40983067
H 2.49112925 2.78995588 12.71727756
H 0.95623930 6.70846857 6.93286524
H 1.01681782 6.35594076 2.97526641
H 2.45972043 3.16428895 16.76209001
H 0.15284630 5.90469716 1.51727381
H 3.29174230 3.64475445 18.22436928
H 2.19108104 4.61699906 1.66091001
H 1.24757074 4.88745394 18.06522938
H 3.19849707 6.05886216 1.77134698
H 0.25457127 3.43542667 17.92448082
H 2.32317183 5.69734848 0.28362405
H 1.09378110 3.79670623 19.43145892
H 2.99439448 8.26340319 1.32106712
H 0.49235955 1.24685190 18.43219602
H 1.96189498 9.65314368 1.67310760
H 1.53336146 -0.13656658 18.08612816
H 3.35230745 9.13002462 3.60910572
H 0.13547041 0.36205338 16.15293305
H 2.76785059 7.47732171 3.68497265
H 0.71741621 2.01630670 16.05520414
H 1.69103878 8.84301932 4.04899275
H 1.79545506 0.64940332 15.70525384
H -1.03790182 7.16156514 19.27630731
H 4.53093631 2.37222978 0.47549933
H -2.68413444 6.53213940 19.29480571
H 6.18395743 2.99928908 0.43528729
H -2.29064469 8.67888962 18.00167154
H 5.77862190 0.83529566 1.73272888
H -2.11398781 9.53354387 19.53467070
H 5.58515046 0.00031232 0.19418204
H -3.63895002 8.73392401 19.11644157
H 7.11968766 0.77620502 0.60704911
H -0.14814140 8.35169338 3.18277095

H 3.63001640 1.19447306 16.55449596
H -0.26724161 9.48849948 1.85370393
H 3.76522887 0.05079805 17.87628817
H 5.25720254 8.31930841 2.12811818
H -1.77619451 1.27037932 17.56434570
H 5.95736213 6.70941457 2.12257609
H -2.50853948 2.86546863 17.67837743
H 5.90800118 7.67288681 0.63499854
H -2.40456022 1.81593163 19.10240290
C 0.51040035 1.33286024 7.24971745
C 2.90045434 8.15656690 12.43212775
C -0.18650870 3.73471362 6.63180921
C 3.59975982 5.75253886 12.86859503
C 2.21296516 3.35696022 7.75878863
C 1.16072435 6.12012412 11.91770557
C 1.78007045 2.07443667 2.34186331
C 1.80335090 7.38555710 17.28878395
C -0.35137705 3.44602245 3.42161458
C 4.01035392 5.97320189 16.35500519
C -0.13236174 1.00662381 4.06598588
C 3.65229977 8.44547663 15.61701816
C 3.74641122 3.75933595 4.57232731
C -0.22761056 5.60903648 15.11368654
C 3.99094290 2.26983613 4.70516498
C -0.50907724 7.09716216 15.03999741
C -0.29947131 2.54815932 12.85660489
C 3.73816407 6.94803576 6.77571571
C -1.59494316 2.21983760 13.55852483
C 5.02799938 7.28111767 6.06648389
C 0.16350502 0.22954181 12.15043901
C 3.27848701 9.26785131 7.48311333
C -0.44294278 9.39665372 11.17581606
C 3.89546472 0.10125847 8.46306105
C -1.11090156 1.70645611 10.65432843
C 4.56525036 7.78835424 8.96858325
C -1.47237812 3.08190270 10.14859918
C 4.95991496 6.40496615 9.42589814
C 1.28713776 2.15926904 11.01801552
C 2.16317456 7.33915285 8.62746740
C 2.52951591 2.09728587 11.87990067
C 0.91777662 7.40070015 7.76998240
C 1.05348321 6.38049334 1.88875109
C 2.40788224 3.14354264 17.84855651
C 2.26940114 5.66294632 1.36699217
C 1.17078346 3.83887258 18.34946377

C 2.16506176 8.61596062 1.93590537
C 1.32201892 0.89677499 17.81720155
C 2.49358642 8.49276164 3.40551461
C 0.99277861 1.00414354 16.34655089
C -2.05151761 7.35160431 19.61951742
C 5.54167863 2.17909391 0.12376631
C -2.55520660 8.65926616 19.05790851
C 6.03798699 0.86496852 0.67566459
C -0.28718167 8.43218555 2.10950421
C 3.77255739 1.10913077 17.62717972
C 6.06008496 7.70997891 1.71101137
C -2.58115019 1.83979241 18.03003933
N 0.06621873 0.38702828 7.77453791
N 3.36656817 9.10721444 11.93701856
N 2.37710679 1.86401377 1.35656480
N 1.29471689 7.61699427 18.31593611
N 0.01444586 1.66516238 11.67484283
N 3.42996908 7.83307372 7.95974282
N 0.95123975 7.83145807 1.47842725
N 2.53053090 1.69595426 18.26470941
O -1.16585970 4.32913602 6.79254979
O 4.56599890 5.18054156 12.59040310
O 2.82048492 3.76444585 8.66229796
O 0.52022402 5.71993871 11.03755055
O 6.49726669 4.13524514 3.06489729
O -3.25993641 5.58135878 17.39894319
O 6.80935540 0.02593012 4.15870799
O -3.35378345 9.47562225 15.63998532
S 2.48409993 1.37403419 5.22134974
S 0.93509595 8.05267916 14.46533438
S 1.98254199 4.18535500 4.78107707
S 1.54017664 5.20889650 14.86029486
Fe 1.27336270 2.83035540 6.41150124
Fe 2.15437182 6.63296068 13.24026790
Fe 0.89703814 2.38555555 3.96988587
Fe 2.65770973 7.03432137 15.62910979
H 4.58777141 5.70648302 15.41613667

5.3.5 $C{\equiv}O_{apical}$

161
Converted from jawg016h-out.cell by c2x
H 4.06052639 4.22495332 3.60902830
H -0.70634088 5.62768959 15.87731126
H 4.27056904 4.40897388 5.35095382

H -0.86865228 5.59887152 14.12124890
H 4.76057354 2.15500085 5.45460212
H -1.36544117 7.86058526 14.26062762
H 4.31251136 1.93860792 3.76033312
H -0.84025882 7.93021335 15.94387023
H 0.57878541 2.55233363 13.43989887
H 2.88265818 7.23607293 6.11577558
H -0.20211820 3.70660898 12.38240952
H 3.71501732 6.09690629 7.15725652
H -1.62833048 3.16736698 14.31391887
H 5.09343158 6.73443285 5.21601866
H -1.63448937 1.45130120 13.91836567
H 5.06189578 8.43338515 5.67598023
H 5.22258059 2.56418853 12.84793094
H -1.76072893 7.30196709 6.69837712
H 0.63928244 0.36629847 13.01333657
H 2.72287274 9.41823465 6.58136214
H -0.97019398 0.06717373 12.33371142
H 4.32171180 9.77442217 7.25570608
H -0.84325961 8.66138004 11.55906257
H 4.07518757 1.11491072 8.01969704
H 0.49918241 9.72697375 11.16675631
H 2.84043131 -0.03418462 8.47859349
H -0.99140177 9.77852002 10.20973110
H 4.36761336 0.03242024 9.37697402
H -0.84108245 1.26738747 9.74472965
H 4.27204231 8.56327817 9.80908764
H 5.64330335 1.33869324 11.04565173
H -2.22162053 8.50142380 8.50094127
H 5.41648863 3.13271976 9.33981694
H -1.90177750 6.72697070 10.19235675
H -0.63286506 3.79425965 9.70095859
H 4.13791244 6.04937949 9.94107944
H -1.90366265 3.89206755 10.92434135
H 5.33730898 5.92477957 8.64803008
H 1.11855845 3.18676488 10.55087278
H 2.36855064 6.50580372 8.95540981
H 1.35919415 1.51490008 10.05503439
H 2.07882184 8.13994312 9.53758794
H 3.35596084 2.32026226 11.12502863
H 0.09613780 7.31544067 8.43004136
H 2.67439795 1.17737140 12.26550446
H 0.72247370 8.58280860 7.39583181
H 2.50446800 2.93478357 12.52384527
H 0.95549151 6.87011734 6.97300896

H 1.02520402 6.49556906 2.98405127
H 2.41123305 3.32254976 16.60544277
H 0.16675909 6.05086632 1.52268344
H 3.27720179 3.76782289 18.06267102
H 2.21300217 4.78001309 1.66173210
H 1.23026546 5.05227060 17.91255817
H 3.21163370 6.22425292 1.78950370
H 0.22984764 3.60647807 17.81879139
H 2.35726508 5.87135292 0.29104381
H 1.10309034 3.98071652 19.30079880
H 2.99602879 8.41674189 1.32491195
H 0.43355233 1.40264550 18.28504992
H 1.96684374 9.80562316 1.68300456
H 1.45994941 0.02352277 17.87870097
H 3.37709036 9.27412757 3.60606011
H 0.05860410 0.57312704 15.97080910
H 2.75935632 7.63322804 3.70208864
H 0.60167528 2.24264046 15.93586351
H 1.71438171 9.02555331 4.06171431
H 1.70412555 0.91222647 15.52002256
H -1.11892385 7.44463344 19.12010193
H 4.54955226 2.38487442 0.47772451
H -2.77937022 6.84150275 19.16119189
H 6.21214455 2.98153713 0.43497880
H -2.32665321 8.99391137 17.86873699
H 5.77418568 0.83457103 1.72786495
H -2.13977653 9.83187655 19.40511333
H 5.55623594 -0.00495116 0.19508324
H -3.68247784 9.07503072 18.97750844
H 7.11017121 0.74356340 0.59771027
H -0.12200999 8.50739534 3.21670844
H 3.54385357 1.30325197 16.37784749
H -0.25499692 9.64511646 1.89102764
H 3.67583789 0.17425266 17.71078903
H 5.27433807 8.49065031 2.17853399
H -1.85059859 1.31880707 17.40066583
H 5.97172500 6.87893072 2.13897818
H -2.53818362 2.93270021 17.44919672
H 5.89385940 7.86437835 0.66686604
H -2.45387870 1.94365331 18.91945302
C 0.48108764 1.40522617 7.19988184
C 2.92637550 8.53497919 12.34154958
C -0.20628595 3.83285812 6.60118062
C 3.56082790 6.06896529 12.97145777
C 2.18868152 3.45138711 7.70910377

C 1.26071599 6.29993537 11.85488931
C 1.76849586 2.13261437 2.28177054
C 1.61194703 7.71796155 17.35147933
C -0.33426503 3.48068228 3.30067145
C 3.73278874 6.33770002 16.32428058
C -0.12838530 1.07131573 4.03871956
C 3.57263931 8.73524640 15.59208944
C 3.74022949 3.85493917 4.57938395
C -0.35020233 6.07338926 14.95161713
C 3.99431533 2.36692164 4.70936641
C -0.57253442 7.57336290 14.95083346
C -0.26509750 2.69470782 12.77167909
C 3.73090413 7.11823627 6.78274289
C -1.56679607 2.45012711 13.49665810
C 5.02141316 7.41858170 6.06033666
C 0.05199329 0.35107680 12.09690244
C 3.31231640 9.44737029 7.49550295
C -0.58651662 9.66205153 11.21368847
C 3.91611546 0.10338336 8.38980959
C -1.15142215 1.87477147 10.59210416
C 4.59019307 7.94946466 8.96934635
C -1.48296964 3.27453623 10.13332253
C 4.95594193 6.56228195 9.44301112
C 1.27143696 2.17837921 10.91075604
C 2.18045881 7.52894678 8.64440747
C 2.50482020 2.13858623 11.78426095
C 0.93439297 7.59005266 7.78709423
C 1.06357299 6.53185668 1.89788413
C 2.37527394 3.29349067 17.69193122
C 2.28854195 5.82782501 1.37388191
C 1.15812091 4.00892097 18.21702894
C 2.17191793 8.76933636 1.94558247
C 1.25067760 1.06645243 17.64562060
C 2.50439470 8.65197138 3.41504137
C 0.90262335 1.22665437 16.18357937
C -2.12530065 7.65127174 19.47430394
C 5.55471369 2.17476750 0.12120123
C -2.60078710 8.97173074 18.92200579
C 6.03002689 0.85261059 0.66987502
C -0.27554284 8.58792724 2.14542047
C 3.70040201 1.22939617 17.44925444
C 6.06757068 7.88482341 1.73986048
C -2.63743669 1.92700918 17.84767312
N 0.04169379 0.45142286 7.71444787
N 3.35925278 9.47918244 11.80749335

N 2.31239146 1.91663318 1.26673926
N 1.03230463 7.94428392 18.34262642
N -0.02054386 1.77861536 11.60022775
N 3.45007341 8.00702091 7.96887262
N 0.95280062 7.98481781 1.49812108
N 2.47517258 1.84162494 18.09640718
O -1.16982878 4.45181185 6.77052902
O 4.48072316 5.38197596 12.81820216
O 2.80144965 3.88613989 8.59364951
O 1.14681244 5.29566381 11.19735129
O 6.52834913 4.14887491 2.83784329
O -3.10852720 5.67301707 16.75061753
O 6.81166977 0.10486181 4.11561757
O -3.34580033 9.68069496 15.48837576
S 2.48675332 1.45734040 5.19606153
S 0.92876092 8.49617913 14.46503658
S 1.96518711 4.26362011 4.74065125
S 1.41422567 5.63481559 14.79026309
Fe 1.24252442 2.91020582 6.36657264
Fe 2.17481981 7.04806900 13.22436202
Fe 0.90306385 2.45033391 3.91812200
Fe 2.50285301 7.38592473 15.72316329
H 0.59123147 7.18407253 11.62300760

5.3.6 $C\equiv N_{basal}$

161
Converted from jawg016j-out.cell by c2x
H 4.04470799 4.08695771 3.53018917
H -0.60830022 5.36319376 15.90520021
H 4.26737645 4.26601963 5.26965847
H -0.82815013 5.11996513 14.17313633
H 4.73617660 2.00383243 5.36481721
H -1.21435547 7.38642901 13.95159742
H 4.26452154 1.79289592 3.67463844
H -0.82108995 7.64612130 15.65416393
H 0.51194799 2.48102966 13.43682776
H 2.92500899 6.93790585 5.99562554
H -0.38136341 3.55226923 12.37569871
H 3.77928438 5.85001683 7.07297862
H -1.75080539 2.89326430 14.30252189
H 5.18368829 6.49426376 5.15112738
H -1.58944424 1.18560877 13.92525165
H 5.07533370 8.19965299 5.56860954
H 5.24067603 2.16995783 12.84754978

H -1.80841130 7.16603658 6.63180824
H 0.80474873 0.27848599 12.96157624
H 2.66351730 9.12459048 6.44632613
H -0.81489954 -0.09918962 12.38144634
H 4.26437080 9.52611417 7.09143050
H -0.64542517 8.31284401 11.46238897
H 4.02566019 1.09103903 7.91923301
H 0.54821182 9.46965459 10.94392996
H 2.85509616 -0.08909806 8.45880026
H -1.01369805 9.37654879 10.10520676
H 4.40151569 0.06549201 9.30270683
H -0.80074500 1.04860620 9.74522715
H 4.21913334 8.36501355 9.68342153
H 5.75334436 1.06744970 11.05617435
H -2.33181504 8.34228727 8.38558451
H 5.40979375 2.78695917 9.32493455
H -1.98062426 6.61143369 10.10855342
H -0.72780020 3.54757118 9.56832664
H 4.14645615 5.86247754 9.88568567
H -1.94288191 3.67173349 10.84897007
H 5.34965440 5.72575312 8.59577456
H 1.03423391 3.20068411 10.65211782
H 2.38306741 6.23127978 8.80953921
H 1.35213235 1.59713047 10.00601302
H 2.06569682 7.84543537 9.42278620
H 3.31631041 2.46826124 11.14644639
H 0.10568506 6.96037103 8.29555501
H 2.75297795 1.09029917 12.07736545
H 0.67300197 8.31334014 7.33248683
H 2.45267098 2.75417599 12.63939029
H 0.97000179 6.63977965 6.80875648
H 1.00229317 6.26814358 2.90346792
H 2.43627692 3.11006429 16.53269151
H 0.16844699 5.78767303 1.43841540
H 3.27288131 3.58865180 17.99604774
H 2.23133385 4.55420291 1.57334227
H 1.21002347 4.82330609 17.85583575
H 3.20225513 6.01256732 1.76412316
H 0.23731572 3.36596978 17.67633250
H 2.39066003 5.68107937 0.23663276
H 1.05332276 3.70315352 19.20036097
H 2.94830760 8.20346256 1.19019521
H 0.48977135 1.18125835 18.23802864
H 1.90540903 9.57369154 1.57917012
H 1.53118656 -0.19584489 17.86655091

H 3.35018833 9.06639950 3.47270657
H 0.09111799 0.30746665 15.96703553
H 2.79169591 7.40412761 3.56183024
H 0.67105504 1.96121170 15.85816844
H 1.70564007 8.75256030 3.95804222
H 1.74190240 0.59439479 15.48261760
H -1.17905276 7.02624155 18.93986788
H 4.61842238 2.35051421 0.49351728
H -2.84403683 6.43088644 19.01128300
H 6.28169474 2.94896218 0.42417238
H -2.42731368 8.57775627 17.70181807
H 5.87500383 0.80090637 1.73582712
H -2.16845621 9.41547196 19.22809535
H 5.61623932 -0.03782191 0.20901584
H -3.73401004 8.67735190 18.86217781
H 7.17951764 0.70644939 0.57214599
H -0.15585750 8.24028450 3.12578470
H 3.59243469 1.13874107 16.30809052
H -0.30910639 9.38044646 1.80476926
H 3.74956697 -0.00304052 17.62621658
H 5.31455404 8.12968647 2.15183266
H -1.87545911 1.24850875 17.27633753
H 6.04530160 6.54143479 2.00611154
H -2.60544475 2.83774171 17.42119814
H 5.90877794 7.60637769 0.59119901
H -2.46538231 1.77491677 18.83694323
C 0.45374443 1.31502502 7.14528501
C 3.38798775 8.05783225 12.40938926
C -0.22861013 3.71143890 6.53355062
C 3.68575255 5.51848430 12.90601802
C 2.18110987 3.32229559 7.64823814
C 1.32268226 6.09722356 11.78703486
C 1.77104566 2.05273481 2.23170280
C 1.63581964 7.33468648 17.19882729
C -0.38602431 3.39596289 3.33869730
C 3.83954265 5.95764454 16.12121957
C -0.15687015 0.97563135 3.97957608
C 3.61283589 8.37826156 15.48151869
C 3.72808557 3.71664275 4.50114973
C -0.27723265 5.68555067 14.92222501
C 3.96474875 2.22518812 4.62831459
C -0.48028900 7.17770716 14.73160286
C -0.34209828 2.53921788 12.76835656
C 3.76688335 6.86304876 6.67784516
C -1.61716661 2.17963594 13.49062915

C 5.06239243 7.19454193 5.97724353
C 0.17462139 0.22859334 12.07520800
C 3.26522697 9.18363924 7.35128125
C -0.52174879 9.32266103 11.07402304
C 3.92066308 0.08740826 8.32785087
C -1.14136169 1.66273878 10.57584174
C 4.56572051 7.74325727 8.86215464
C -1.53943365 3.02824017 10.07054381
C 4.95600774 6.37644726 9.37435986
C 1.24689248 2.17192536 10.92325064
C 2.17499992 7.25457953 8.51631907
C 2.49898431 2.10246711 11.77000234
C 0.92522313 7.30883902 7.66518909
C 1.05199353 6.28981146 1.81761187
C 2.38821812 3.08746901 17.61845451
C 2.29671615 5.61005358 1.31538122
C 1.14456471 3.76921215 18.12102075
C 2.13151697 8.53886422 1.82981824
C 1.30950881 0.83773206 17.60661410
C 2.49583901 8.41624817 3.29092469
C 0.95325601 0.94879647 16.14249470
C -2.17915774 7.23798710 19.30928911
C 5.61947620 2.13970022 0.12591411
C -2.65701122 8.56005715 18.76533070
C 6.10219507 0.81955133 0.67142436
C -0.31587704 8.32251982 2.05498957
C 3.75585650 1.05559485 17.37821642
C 6.11085098 7.56975222 1.65941477
C -2.67053924 1.80986145 17.76941424
N -0.00142771 0.37596282 7.67304919
N 2.97327971 8.95775065 11.69681006
N 2.36062065 1.87075980 1.23590140
N 1.02141829 7.54632932 18.17462876
N -0.01216080 1.65221705 11.58974764
N 3.43711784 7.75917551 7.84783469
N 0.91842066 7.73562276 1.40038532
N 2.52221802 1.64131073 18.03548923
O -1.21076408 4.29829499 6.70542667
O 4.64795820 4.89228171 12.73453460
O 2.80385326 3.72090731 8.54467145
O 0.64259176 5.76029899 10.90247705
O 6.51977858 4.04766255 2.99941766
O -3.06055903 5.30664598 16.45073407
O 6.84859571 -0.01399793 4.09140041
O -3.40383376 9.37999039 15.35183456

S 2.45032113 1.33710911 5.13332208
S 1.06711730 8.02651675 14.25283458
S 1.96135728 4.14710907 4.67441341
S 1.48049292 5.20927135 14.78178583
Fe 1.23044281 2.80305055 6.30447466
Fe 2.28992002 6.51102037 13.15310426
Fe 0.88003294 2.34601985 3.86315028
Fe 2.57850420 7.01188470 15.59662532
H 4.44044604 8.01502348 12.74573923

5.3.7 C≡N_{basal}

161
Converted from jawg016k-out.cell by c2x
H 4.01843434 4.10597893 3.59693073
H -0.54349669 5.38454471 16.03499991
H 4.27901568 4.29744676 5.33208951
H -0.72569082 5.20840149 14.28707608
H 4.74303425 2.04134090 5.44042590
H -1.18975418 7.45228171 14.18018249
H 4.25777054 1.81847932 3.75580710
H -0.76622720 7.66529335 15.88543500
H 0.55978935 2.57074377 13.58051618
H 2.96965399 6.96297787 6.07918886
H -0.29286541 3.68377423 12.53142402
H 3.83300965 5.86871580 7.14264439
H -1.67103176 3.02421324 14.45815307
H 5.22165387 6.52474690 5.22227913
H -1.60117642 1.32514743 14.01517468
H 5.10427483 8.22763018 5.63437871
H 5.27683543 2.43496119 12.98787469
H -1.75452797 7.17049895 6.69245429
H 0.81477618 0.38163386 13.08827530
H 2.67715141 9.14472270 6.55933345
H -0.79677549 0.00691346 12.47629759
H 4.30439809 9.53486367 7.14586993
H -0.49291280 8.43524664 11.66276570
H 3.93645728 1.10828304 7.88609925
H 0.60523126 9.65184571 11.06367623
H 2.95375310 -0.10848614 8.65691675
H -0.96694668 9.44968381 10.29282547
H 4.60544812 0.23801600 9.24744435
H -0.78761965 1.16315164 9.89217418
H 4.28824430 8.36551857 9.77440010
H 5.77453562 1.29479601 11.21310754

H -2.26948929 8.32790973 8.47277102
H 5.45680176 2.97457761 9.44975998
H -1.92247262 6.57826663 10.18711997
H -0.64273838 3.64393540 9.63609106
H 4.19959079 5.86591071 9.94994118
H -1.82018904 3.85566548 10.93936782
H 5.41029174 5.73938480 8.66523683
H 1.08187592 3.32505251 10.78285197
H 2.43484396 6.21816126 8.86054581
H 1.38692676 1.72267246 10.13437593
H 2.11835079 7.80658532 9.52274252
H 3.36625335 2.51699582 11.26605376
H 0.16095131 6.96210872 8.37651942
H 2.73604029 1.22688233 12.29202958
H 0.71832625 8.34765862 7.45346686
H 2.51226554 2.92838927 12.73350327
H 1.02072247 6.69146762 6.87804100
H 1.03492665 6.26446466 2.94424959
H 2.45243110 3.23973394 16.68874968
H 0.20950706 5.79455913 1.47145224
H 3.29031171 3.70983509 18.15171678
H 2.27843230 4.56471974 1.61374684
H 1.22431751 4.94555292 17.99690849
H 3.24434485 6.03064203 1.79699471
H 0.25150798 3.48249390 17.85794122
H 2.42802234 5.69425836 0.27574570
H 1.08929773 3.84853891 19.36310531
H 2.99160411 8.20987634 1.23804039
H 0.49722875 1.30755267 18.38984611
H 1.94806731 9.57816989 1.62995829
H 1.53816430 -0.07275863 18.01860087
H 3.38618359 9.06110424 3.52714230
H 0.10600209 0.44370923 16.11342501
H 2.81778169 7.40274722 3.61336334
H 0.69294635 2.09746850 16.01862805
H 1.73681883 8.75917707 4.00117715
H 1.75976229 0.72398350 15.64349849
H -1.13908907 7.13894083 19.13663187
H 4.62496580 2.37706391 0.49234827
H -2.80483706 6.54946039 19.20918771
H 6.28594291 2.97090143 0.42460672
H -2.38334886 8.69604428 17.90553122
H 5.87413990 0.82822750 1.74248726
H -2.11880764 9.52928250 19.43223663
H 5.62147679 -0.01594152 0.21660948

H -3.68962181 8.79750136 19.06511619
H 7.18402594 0.73168496 0.58321071
H -0.11186891 8.24436445 3.17471675
H 3.60572872 1.25877398 16.46575052
H -0.26473634 9.38764531 1.85630065
H 3.76007760 0.11601911 17.78010872
H 5.35358730 8.17837894 2.20190541
H -1.86223894 1.33173559 17.43211898
H 6.07643103 6.58634404 2.07148021
H -2.58804250 2.92350773 17.56449936
H 5.94210739 7.63567560 0.64538107
H -2.44757950 1.87633921 18.99081062
C 0.46835393 1.33303253 7.26343379
C 2.91466677 8.03771902 12.44223756
C -0.21600535 3.72925584 6.62876708
C 3.62356467 5.70299177 12.91509612
C 2.19790964 3.35421049 7.74699174
C 1.09408869 6.08474649 11.90354313
C 1.75181601 2.07434461 2.33807724
C 1.62993424 7.43026597 17.44381836
C -0.38914522 3.42557106 3.42632540
C 3.88018491 6.08397539 16.40688812
C -0.16587308 1.00232306 4.06098270
C 3.67059535 8.47926340 15.68840226
C 3.72117303 3.74557474 4.57800323
C -0.19483953 5.74627877 15.07024954
C 3.96422741 2.25517797 4.70892184
C -0.43522356 7.24219185 14.93814884
C -0.28872054 2.66416517 12.90860537
C 3.81584014 6.88399803 6.75518060
C -1.57777869 2.33592620 13.61955610
C 5.10292464 7.22480193 6.04781249
C 0.19713775 0.34680345 12.19297934
C 3.31326930 9.19679369 7.44039975
C -0.44624805 9.44024744 11.24805853
C 3.97438800 0.13252980 8.36721970
C -1.10270433 1.81091440 10.70854663
C 4.62344385 7.75027266 8.94441973
C -1.46215753 3.17422674 10.17090047
C 5.01286331 6.38193054 9.44799945
C 1.28335531 2.29454760 11.05342044
C 2.22901706 7.24924247 8.59564513
C 2.53166302 2.22363687 11.90574471
C 0.97535682 7.33406227 7.75395977
C 1.09158279 6.29405528 1.85817566

```
C 2.40362470 3.21231706 17.77469255
C 2.33929026 5.62055719 1.35457641
C 1.16385651 3.89622155 18.28147497
C 2.17305688 8.54208244 1.87726990
C 1.31930517 0.96294285 17.76182696
C 2.52940198 8.41643095 3.34044886
C 0.96851427 1.08172947 16.29753973
C -2.13745884 7.35320790 19.50900291
C 5.62463385 2.16036515 0.12692418
C -2.61276572 8.67798788 18.96877306
C 6.10625253 0.84197101 0.67910130
C -0.27209566 8.32895300 2.10402468
C 3.76591943 1.17581417 17.53613467
C 6.14567851 7.60982109 1.71345481
C -2.65314930 1.90003940 17.92295315
N 0.00834562 0.40359000 7.80524726
N 3.41706309 9.07078822 12.02582675
N 2.33967708 1.87502754 1.34607136
N 1.00918425 7.66204116 18.41101790
N 0.02461822 1.78085986 11.72037022
N 3.48981969 7.77207575 7.93540811
N 0.95912612 7.74144050 1.44711487
N 2.53307828 1.76497504 18.19103458
O -1.19576846 4.32481494 6.78877611
O 4.59246652 5.08370123 12.75714419
O 2.82143906 3.77271939 8.63564446
O 0.57600632 5.74976478 10.91006980
O 6.51880768 4.10403113 3.05954595
O -3.03751359 5.40078916 16.80357136
O 6.83533581 0.03887930 4.13942747
O -3.36084293 9.46607450 15.54543576
S 2.45461716 1.36085300 5.22186090
S 1.08117176 8.14444087 14.48369759
S 1.95637998 4.17234699 4.78045537
S 1.57107838 5.30141979 14.93053929
Fe 1.24397074 2.82121103 6.41448388
Fe 2.18696073 6.63837735 13.14748782
Fe 0.86852333 2.37536921 3.97147810
Fe 2.62777093 7.11614522 15.82687813
H 3.00200923 9.56535454 11.24449381
```

5.3.8 $C{\equiv}\mathrm{N}_{\mathrm{apical}}$

```
161
Converted from jawg016l-out.cell by c2x
```

H 4.04652005 4.11736731 3.52217087
H -0.59464686 5.37271228 15.94764949
H 4.26384889 4.29657603 5.26313923
H -0.84292377 5.18197477 14.21615887
H 4.73701326 2.03207380 5.35814804
H -1.24626757 7.45390674 14.07248868
H 4.27001845 1.82381753 3.66643143
H -0.82425752 7.65768097 15.78337217
H 0.49657523 2.46368763 13.43750320
H 2.92711702 6.99513423 6.02443067
H -0.35493663 3.57014325 12.37707720
H 3.78150312 5.89136838 7.08684105
H -1.75619744 2.90752953 14.28780905
H 5.17569469 6.55543997 5.16610802
H -1.65196338 1.20110418 13.85616408
H 5.07578565 8.25955357 5.60589150
H 5.25166219 2.25762669 12.78965987
H -1.82604715 7.19842783 6.66475543
H 0.75158946 0.28449292 12.96474438
H 2.68012649 9.17687119 6.49139066
H -0.84887598 -0.10883917 12.30908190
H 4.27974473 9.56886604 7.14784399
H -0.61130750 8.35635056 11.48105487
H 4.05434318 1.10820609 7.98355042
H 0.57801160 9.53822132 10.98776374
H 2.84946542 -0.06723948 8.45841015
H -0.96557920 9.41956934 10.12032358
H 4.38568987 0.03289454 9.33961522
H -0.80528658 1.09291684 9.72470439
H 4.23884684 8.37194958 9.73329212
H 5.77346483 1.09388948 11.01850292
H -2.34292731 8.36353036 8.43519749
H 5.42214095 2.85154705 9.32121577
H -1.99193473 6.61157776 10.13615476
H -0.73460064 3.59995531 9.58532771
H 4.16274812 5.86931664 9.88403924
H -1.94650881 3.70271294 10.86834124
H 5.36869554 5.75652538 8.59301575
H 1.03834131 3.20229635 10.62915454
H 2.38676039 6.26625895 8.83802889
H 1.35568712 1.58960219 10.00770519
H 2.07195545 7.88012130 9.45233732
H 3.31472416 2.46343611 11.14519140
H 0.11263809 7.00590591 8.32010989
H 2.73969660 1.11131724 12.10196850

H 0.68687933 8.35261531 7.35581278
H 2.44409746 2.78672773 12.62599764
H 0.97984690 6.67502010 6.83836275
H 1.01768559 6.29082352 2.92362674
H 2.36300282 3.18020319 16.51046610
H 0.16746075 5.82432998 1.46556081
H 3.20566252 3.65948481 17.96810826
H 2.22412717 4.57737236 1.59564893
H 1.08452091 4.84389041 17.77247505
H 3.20347021 6.03179169 1.75063556
H 0.16782817 3.34726906 17.72792512
H 2.36410041 5.68158454 0.23913124
H 1.02736534 3.80673267 19.19485601
H 2.96129590 8.21683784 1.21150421
H 0.44902784 1.21031300 18.21269800
H 1.93170193 9.60003286 1.58318344
H 1.49950229 -0.14623495 17.79970024
H 3.37307202 9.09470389 3.48516693
H 0.05342566 0.37107327 15.92043282
H 2.79725933 7.43856478 3.58597225
H 0.59850230 2.03851786 15.84643979
H 1.72793536 8.80318531 3.97301172
H 1.69408198 0.69861216 15.44011862
H -1.23079244 7.09536071 18.95309503
H 4.62287322 2.35833634 0.45982785
H -2.89257058 6.51102519 19.03823974
H 6.28863035 2.94634429 0.39423471
H -2.43936116 8.66600142 17.72766934
H 5.87748337 0.79141100 1.69348678
H -2.19682988 9.49397191 19.25957005
H 5.59172289 -0.02654463 0.15935212
H -3.76122319 8.77218099 18.87151936
H 7.17155969 0.69514796 0.51881117
H -0.10708989 8.29520938 3.16235866
H 3.54951321 1.23197330 16.27303976
H -0.28251923 9.42322822 1.83089298
H 3.70333512 0.06689105 17.56926310
H 5.36783394 8.19712858 2.23259974
H -1.94260157 1.31513776 17.25862175
H 6.08556940 6.60009405 2.08898766
H -2.67429284 2.90074764 17.42038362
H 5.93185409 7.64524525 0.66481739
H -2.52950054 1.82396874 18.82605786
C 0.46022520 1.32937131 7.12177216
C 2.98535891 8.13466972 12.31170393

C -0.23072860 3.73474957 6.53248294
C 3.66682398 5.73037783 12.90426087
C 2.17491558 3.35082340 7.64005366
C 1.24754177 6.12432334 11.80038198
C 1.76958212 2.06700973 2.22047156
C 1.85454203 7.22995554 17.42835218
C -0.38785533 3.41872368 3.33394657
C 3.87830834 5.94811892 16.06736331
C -0.16343532 1.00147755 3.97425466
C 3.62379337 8.42257020 15.54064774
C 3.72876222 3.74591590 4.49237545
C -0.28350926 5.73155898 14.97045664
C 3.96764568 2.25474879 4.61969131
C -0.50248529 7.22656426 14.83632748
C -0.34514610 2.55085167 12.75748965
C 3.76990688 6.90963952 6.70378057
C -1.63885780 2.21157587 13.45750140
C 5.06246376 7.24771324 6.00012526
C 0.15178740 0.23671643 12.05786976
C 3.27847018 9.22519703 7.39917845
C -0.49101921 9.36821900 11.09765369
C 3.92076497 0.09469592 8.35831195
C -1.14740409 1.69482554 10.56345937
C 4.57749077 7.76744790 8.89461769
C -1.54332966 3.06973731 10.08069906
C 4.97032853 6.39326693 9.38034726
C 1.24429840 2.17660992 10.91597291
C 2.18244045 7.29052686 8.54576574
C 2.49093997 2.11700006 11.77056280
C 0.93543848 7.34823994 7.69137326
C 1.05718153 6.31989953 1.83751296
C 2.32400214 3.14796663 17.59609982
C 2.28939997 5.62896148 1.32067574
C 1.07416182 3.80962227 18.11075860
C 2.14750746 8.56613991 1.84662149
C 1.26704438 0.89108541 17.56749840
C 2.51274784 8.45168484 3.30781963
C 0.90176004 1.02677300 16.10855695
C -2.22424804 7.31783564 19.33391589
C 5.62144154 2.14401284 0.08894295
C -2.68429471 8.64408799 18.78675097
C 6.09662387 0.81657739 0.62753042
C -0.28812443 8.36792188 2.09464909
C 3.71133672 1.12988803 17.34126291
C 6.14687221 7.62399630 1.73036454

C -2.73654138 1.87027560 17.75928457
N 0.01191606 0.37812068 7.63193996
N 3.43822714 9.08170714 11.79499961
N 2.36233021 1.87542583 1.22838091
N 0.77839927 7.67148400 17.81529837
N -0.01849416 1.66712911 11.57754497
N 3.44608358 7.79557264 7.88133063
N 0.92936233 7.76821802 1.42349756
N 2.47435644 1.70216930 18.00376580
O -1.21355831 4.31828504 6.71089842
O 4.64745658 5.13892654 12.73053943
O 2.79565084 3.75242941 8.53531716
O 0.63183923 5.71076751 10.90356284
O 6.53496229 4.08209480 2.98910236
O -3.07104918 5.25994985 16.43027770
O 6.85959862 0.02207779 4.06814444
O -3.43946835 9.43770446 15.46773660
S 2.45400631 1.36377607 5.12208156
S 1.03482882 8.10101539 14.38284471
S 1.95963519 4.17324282 4.66492900
S 1.47436859 5.27037112 14.76606420
Fe 1.22946932 2.82923533 6.29186607
Fe 2.20863191 6.63856281 13.13988778
Fe 0.87749599 2.36773092 3.85115800
Fe 2.62069916 7.02404256 15.59768264
H 2.58113177 6.83694277 18.17419575

5.3.9 C≡$\boldsymbol{N}_{\mathbf{apical}}$

161
Converted from jawg016m-out.cell by c2x
H 4.08051854 4.12284224 3.62545953
H -0.70213391 5.26927681 15.76639739
H 4.26608862 4.31567681 5.36774402
H -0.83702615 5.14355055 14.01157783
H 4.76416180 2.06273364 5.48940259
H -1.35606667 7.38263692 13.96887727
H 4.33732175 1.83633597 3.79160749
H -0.96946555 7.54623119 15.68490204
H 0.53880687 2.43015168 13.49324126
H 2.88386758 7.05101032 6.10834836
H -0.31597253 3.54755590 12.44953395
H 3.71769648 5.91970056 7.15803288
H -1.70806776 2.87145888 14.36679845
H 5.11641519 6.56929898 5.23670896

H -1.61538962 1.17009447 13.91399166
H 5.06325787 8.26907900 5.69536645
H 5.21402051 2.24390672 12.87709156
H -1.79129683 7.17092381 6.72803075
H 0.73293216 0.23607355 13.00960699
H 2.70648109 9.23213763 6.57936640
H -0.86152238 -0.12410626 12.32252259
H 4.29729669 9.59617562 7.27073487
H -0.62087626 8.40446159 11.47956500
H 4.07470617 1.07535125 8.11637367
H 0.59637486 9.57243545 11.02477685
H 2.83212706 -0.08066749 8.54305770
H -0.93049514 9.48989444 10.12479903
H 4.34259835 -0.02237189 9.46845690
H -0.79299984 1.10382556 9.77509202
H 4.23324436 8.37669060 9.82238839
H 5.71352267 1.11849899 11.07642110
H -2.27830100 8.34877074 8.51433666
H 5.39397282 2.89484911 9.39034579
H -1.95679817 6.57591448 10.19923148
H -0.68548612 3.62083681 9.66753506
H 4.11188563 5.87139635 9.95230953
H -1.90800991 3.71695647 10.94092938
H 5.31427969 5.74930289 8.65915321
H 1.07327800 3.18132960 10.68799481
H 2.34907476 6.31149531 8.93303981
H 1.39000868 1.56502988 10.07411081
H 2.04737214 7.93194696 9.53245434
H 3.35286620 2.37958631 11.21193689
H 0.07556347 7.11672986 8.41163935
H 2.72511079 1.09779882 12.22921749
H 0.70015696 8.38912293 7.38008645
H 2.47823853 2.80691056 12.66638074
H 0.93802173 6.67912433 6.95174047
H 0.95866170 6.31639747 2.96573128
H 2.37623107 3.14062454 16.56859563
H 0.14802669 5.86896314 1.47869049
H 3.21596377 3.68497545 18.00689606
H 2.20344961 4.60457128 1.69021629
H 1.10684844 4.87632078 17.70658065
H 3.18335466 6.06089945 1.83300023
H 0.17581295 3.38497442 17.79184190
H 2.36423283 5.68757302 0.31732246
H 1.05330911 3.96005342 19.20812951
H 2.97141652 8.24763012 1.34346054

H 0.43842840 1.25977236 18.35954585
H 1.92926904 9.62869515 1.71288348
H 1.48940361 -0.11185731 18.01284777
H 3.33906774 9.04401700 3.63229118
H 0.05332609 0.32799889 16.09523706
H 2.69047677 7.41285463 3.70253614
H 0.61358624 1.98558313 15.95375946
H 1.67198879 8.81941703 4.07438449
H 1.69943085 0.61870006 15.61704324
H -1.07627033 7.15759618 19.20001468
H 4.57742011 2.62659045 0.43167777
H -2.70721468 6.48697435 19.18977354
H 6.28923531 3.07251036 0.32791865
H -2.39345694 8.62443901 17.89981307
H 5.86192009 1.14169920 1.79503802
H -2.15635601 9.49726135 19.41883874
H 5.18836672 0.22052810 0.46344218
H -3.69650731 8.70752586 19.06317386
H 6.91394126 0.66509038 0.47758921
H -0.13729318 8.41119953 3.16908853
H 3.57053143 1.19535107 16.44111779
H -0.32327234 9.44418748 1.77295759
H 3.68335653 0.07169948 17.77446947
H 5.26129991 8.25546011 2.06690433
H -1.87378529 1.28455984 17.44371257
H 5.98723536 6.67398362 2.32022146
H -2.58756255 2.87956941 17.59877262
H 5.96502746 7.38836305 0.70751019
H -2.47562128 1.80401152 19.00679801
C 0.46390055 1.31175709 7.18248920
C 2.95491070 8.20034555 12.35647536
C -0.21091355 3.72867794 6.59265758
C 3.64713223 5.78837653 12.90959036
C 2.18150808 3.34048172 7.70803046
C 1.21878611 6.16425560 11.78458789
C 1.84858907 2.02786111 2.29059149
C 1.45340631 7.33631193 17.28522620
C -0.32519374 3.37269696 3.32553559
C 3.66358366 6.14668450 16.33855120
C -0.11542822 0.97528956 4.02928040
C 3.39715743 8.49366919 15.67859376
C 3.74921353 3.75517712 4.59253703
C -0.33858815 5.66940049 14.82182027
C 4.00665846 2.26892603 4.73413347
C -0.60848806 7.16322456 14.73105326

C -0.30900549 2.52509613 12.82140490
C 3.72732970 6.94118425 6.78395872
C -1.59954315 2.18549312 13.52772431
C 5.02537206 7.25391817 6.07908981
C 0.14780646 0.21268470 12.09258429
C 3.28747723 9.26072621 7.49904891
C -0.47749826 9.41925057 11.11190918
C 3.90888644 0.05992345 8.47329618
C -1.12614555 1.70729031 10.61647764
C 4.55975484 7.76882671 8.98086343
C -1.50345438 3.09163901 10.14779974
C 4.92882727 6.38463059 9.45389500
C 1.27299927 2.15514257 10.97931509
C 2.15615132 7.33504962 8.63108925
C 2.51196027 2.09358851 11.84608338
C 0.91291065 7.39580995 7.76963294
C 1.03296372 6.34964641 1.88166036
C 2.33139491 3.16588387 17.65450657
C 2.27473264 5.65103556 1.39810352
C 1.08833101 3.87117092 18.12701194
C 2.13812962 8.58811507 1.95830873
C 1.25903285 0.91325524 17.73063705
C 2.45751400 8.43914248 3.42679949
C 0.90560707 0.98408581 16.26368715
C -2.10060920 7.32174359 19.52934063
C 5.55465579 2.31237354 0.07646825
C -2.61475708 8.61980318 18.96504767
C 5.90517369 0.99602854 0.71771671
C -0.30502741 8.40740460 2.09760020
C 3.70725812 1.12690421 17.51498290
C 6.08408510 7.60365642 1.76669937
C -2.66545400 1.84960415 17.93671959
N 0.01067989 0.36346456 7.69432699
N 3.40788316 9.16128243 11.86480857
N 2.44498061 1.82729302 1.30179891
N 1.23146507 7.68346214 18.45252690
N 0.00225152 1.65251498 11.63214115
N 3.42769097 7.82256653 7.96836180
N 0.93437309 7.80149213 1.47495405
N 2.46766419 1.73809927 18.13744005
O -1.18521048 4.32843910 6.76070969
O 4.61474847 5.17540393 12.72115860
O 2.79857573 3.75383329 8.59956724
O 0.63194654 5.73368896 10.87451744
O 6.53601024 4.02038426 2.92221293

O -3.14649634 5.57638729 16.77037646
O 6.82857499 0.00295642 4.12164289
O -3.56391246 9.48888323 15.67796251
S 2.49805070 1.35714049 5.20982051
S 0.88998860 8.11725767 14.32865932
S 1.97368187 4.16023421 4.73419736
S 1.44044651 5.26878083 14.71775842
Fe 1.24237323 2.81253441 6.35563893
Fe 2.17894259 6.68177646 13.13666439
Fe 0.92921164 2.34180844 3.90468570
Fe 2.37403857 7.12182898 15.74811634
H 1.96192297 8.18999173 18.96713791

5.3.10 S *cis* to CN

161
Converted from jawg016n-out.cell by c2x
H 4.04211087 4.06781821 3.53655199
H -0.76527156 5.43912674 15.95239882
H 4.26328191 4.25742732 5.27813843
H -1.22591430 5.28406751 14.16186026
H 4.73606169 2.00202723 5.39205450
H -1.33847765 7.74947913 14.03611550
H 4.28624654 1.78172766 3.69728416
H -0.86357612 7.87662037 15.74720071
H 0.48137211 2.36767813 13.48943229
H 2.95730049 6.93369071 5.99899695
H -0.36358786 3.47226656 12.42547601
H 3.81184108 5.83214552 7.06154389
H -1.74517424 2.81046466 14.35178153
H 5.21076251 6.50565793 5.14592605
H -1.67909267 1.11296793 13.89906947
H 5.10639618 8.20381613 5.58980522
H 5.26320074 2.20841456 12.83943720
H -1.82587739 7.12567051 6.66091016
H 0.73264314 0.18948721 13.02767454
H 2.69995092 9.11351059 6.47381935
H -0.86463882 -0.20660153 12.36939457
H 4.29789818 9.50874144 7.12894694
H -0.61300642 8.19782299 11.47207832
H 4.05234253 1.09482988 8.03970659
H 0.59470110 9.37141505 11.00818916
H 2.84448410 -0.07915337 8.50254344
H -0.92945250 9.27018291 10.10962358
H 4.37214845 0.01385744 9.39614095

H -0.82860705 0.96385502 9.78465943
H 4.25915929 8.29025548 9.72263605
H 5.77996576 1.01635625 11.06631053
H -2.34881025 8.30370008 8.43700538
H 5.44156658 2.75287438 9.34335872
H -1.99284030 6.51279055 10.11920401
H -0.73252596 3.45831642 9.59884046
H 4.21032853 5.77084126 9.79058904
H -1.93575074 3.60514047 10.88747214
H 5.44631379 5.72341901 8.52832569
H 1.01763760 3.09484823 10.67220649
H 2.42776443 6.19663573 8.81949392
H 1.33814064 1.48367324 10.05615717
H 2.09877311 7.80838962 9.43232607
H 3.29653327 2.36647179 11.18747500
H 0.14799194 6.90909378 8.29714690
H 2.73265229 1.01610377 12.15272647
H 0.70508238 8.26917649 7.34156333
H 2.42425912 2.69132561 12.66691746
H 1.02183657 6.59941455 6.81459196
H 1.04734040 6.26017398 2.89571451
H 2.41041718 3.01837099 16.68826409
H 0.20057577 5.78631479 1.43573804
H 3.22668577 3.48372548 18.16603831
H 2.26631068 4.54273376 1.56613779
H 1.15592230 4.72050243 17.97310794
H 3.24114039 6.00369994 1.71843182
H 0.19213689 3.25490311 17.82204419
H 2.39875342 5.65307866 0.21314521
H 1.00638513 3.62467205 19.33875845
H 2.99574954 8.18844936 1.19075282
H 0.43881250 1.07403823 18.34177300
H 1.95340481 9.56394274 1.57108162
H 1.48738342 -0.29904604 17.96459904
H 3.39294879 9.05029376 3.46909804
H 0.04617001 0.25526132 16.06341699
H 2.82582871 7.39210026 3.55996072
H 0.67890706 1.88768370 15.96205119
H 1.74441809 8.74842481 3.94638340
H 1.71075037 0.48346590 15.59846834
H -1.21498023 6.92980159 19.07517845
H 4.64073709 2.31192417 0.48163528
H -2.86773055 6.32058081 19.13566778
H 6.29688161 2.92558407 0.43955620
H -2.46703106 8.46063454 17.81920575

H 5.89300967 0.75891306 1.71927464
H -2.23319101 9.31479746 19.34178590
H 5.68326889 -0.06697013 0.17697655
H -3.78569338 8.55376478 18.96668705
H 7.22429395 0.70569683 0.58579830
H -0.11851047 8.23433537 3.11149682
H 3.55132027 1.05003240 16.44300062
H -0.26355147 9.37491748 1.79021399
H 3.71162668 -0.09813969 17.75413444
H 5.41164913 8.16153353 2.09905184
H -1.97401779 1.13399153 17.43611073
H 6.13357811 6.56904438 1.96963287
H -2.70622493 2.72029105 17.58926753
H 6.00867096 7.61776843 0.54345106
H -2.55923705 1.65834405 19.00091357
C 0.46130974 1.27966363 7.15131342
C 2.97374735 8.02848817 12.33379272
C -0.23254153 3.67765377 6.55172972
C 3.79381718 5.68166340 12.74887431
C 2.17817929 3.29376595 7.66335956
C 1.29890019 5.97857564 11.81333023
C 1.77950345 2.05056713 2.23241134
C 1.63370182 7.16407346 17.21043582
C -0.38901774 3.37623419 3.34266324
C 3.83144925 5.93194868 16.36401630
C -0.16315633 0.95934109 3.98658571
C 3.57122974 8.29379425 15.47207429
C 3.72619984 3.70405846 4.51066941
C -0.93381425 5.90194773 14.99527368
C 3.97130308 2.21472802 4.64545927
C -0.71656381 7.32061525 14.82193005
C -0.35786680 2.45378710 12.80558184
C 3.79944367 6.85042045 6.68010121
C -1.65175160 2.12567926 13.50951069
C 5.09302655 7.19247181 5.98275888
C 0.13693973 0.13645742 12.11742573
C 3.29848701 9.16125846 7.38199700
C -0.47753587 9.21054603 11.09779479
C 3.91684992 0.08057863 8.40990701
C -1.16556443 1.58178144 10.61445282
C 4.60776736 7.70623306 8.87306041
C -1.54769555 2.95231960 10.10882860
C 5.02009306 6.32661281 9.32729850
C 1.22695126 2.07135291 10.96360785
C 2.21447522 7.21896545 8.52631650

C 2.47560077 2.01768273 11.81522501
C 0.96709031 7.26628956 7.67181767
C 1.09070757 6.28123310 1.80996441
C 2.34518220 2.99028633 17.77174010
C 2.32636471 5.59535214 1.29432015
C 1.09671161 3.67129220 18.25851031
C 2.17678481 8.52857753 1.82426926
C 1.26600376 0.73849448 17.71638286
C 2.53602009 8.40482795 3.28556932
C 0.92780307 0.86685024 16.24916904
C -2.21809183 7.13611853 19.43796428
C 5.64775608 2.11214401 0.12341825
C -2.70651567 8.44980917 18.88075821
C 6.14284041 0.79320455 0.66098972
C -0.27179517 8.31619889 2.03962983
C 3.71167749 0.96245502 17.51308608
C 6.20496485 7.59299812 1.61286179
C -2.76784598 1.69181532 17.93419383
N 0.01617428 0.33601070 7.67957031
N 3.38639037 8.98656090 11.80614358
N 2.37437945 1.88451544 1.23661751
N 1.00846434 7.36303424 18.18105717
N -0.03436140 1.56369055 11.62989309
N 3.47378141 7.73207092 7.86089042
N 0.96356224 7.72740120 1.39249015
N 2.47358191 1.53925045 18.16832051
O -1.22229736 4.24949324 6.72919840
O 4.78377322 5.15804600 12.45926558
O 2.79786784 3.68631103 8.56470269
O 0.64047553 5.61099461 10.93136713
O 6.56287121 4.05424888 2.97733586
O -3.20010962 5.30338080 16.95101580
O 6.88560412 0.00421832 4.07486165
O -3.53424347 9.29393256 15.40002606
S 2.45473619 1.31832700 5.13615259
S 1.02857475 7.87835380 14.34054774
S 1.95681227 4.12755105 4.68467714
S 1.77030479 4.94386864 14.71851397
Fe 1.23182140 2.77757784 6.31398099
Fe 2.30255572 6.47427117 13.13572160
Fe 0.87925664 2.32789597 3.86571747
Fe 2.60048357 6.88025608 15.62119994
H 2.86400695 4.14771357 14.71266941

5.3.11 S *trans* to CN

```
161
Converted from jawg016o-out.cell by c2x
H 4.05442394 4.12107864 3.55831120
H -0.56136835 5.06968158 16.07231585
H 4.28715857 4.30317185 5.29687660
H -0.84485999 4.91892091 14.32690520
H 4.75336274 2.04391984 5.39814984
H -1.56115401 7.24419795 14.20733920
H 4.28347042 1.83063121 3.70743217
H -1.00835968 7.51144551 15.95380292
H 0.50149696 2.47094316 13.49662983
H 2.96032240 7.00682688 6.04446295
H -0.38362491 3.56469626 12.44894653
H 3.83474732 5.90462702 7.09144873
H -1.76185980 2.84735381 14.36338807
H 5.19064578 6.55881129 5.15007920
H -1.60508114 1.15272173 13.91934746
H 5.09631507 8.26365355 5.57235643
H 5.25874866 2.22710317 12.86728638
H -1.78258435 7.17944996 6.62300076
H 0.77517809 0.29396715 13.00665543
H 2.73817824 9.19673423 6.51638916
H -0.81983390 -0.10924638 12.35694965
H 4.34002202 9.58323848 7.16572571
H -0.55444624 8.38665642 11.50474419
H 4.07795512 1.10184391 8.03177623
H 0.63908123 9.56136549 11.00022236
H 2.86963548 -0.06947184 8.50268822
H -0.90764339 9.45289390 10.14588062
H 4.40777669 0.02007574 9.38200441
H -0.78400002 1.10920089 9.77008007
H 4.33891677 8.39667137 9.72596553
H 5.79047129 1.12957247 11.05968147
H -2.26203847 8.31555142 8.41555962
H 5.43303052 2.90493432 9.37904642
H -1.92521873 6.58261952 10.14113825
H -0.72749793 3.61428680 9.64457622
H 4.21540035 5.90806011 9.92055606
H -1.94051852 3.70830002 10.92914138
H 5.40522001 5.74157669 8.62024859
H 1.04114649 3.23536074 10.70967160
H 2.46331705 6.26891204 8.84765975
H 1.35934083 1.63525641 10.05723110
```

H 2.16264664 7.87575146 9.48852886
H 3.32183084 2.48746451 11.20568430
H 0.17810395 7.03945541 8.37021231
H 2.74545282 1.11836372 12.13806916
H 0.76018171 8.37676770 7.39769765
H 2.45622031 2.78225744 12.69516489
H 1.02905297 6.69597764 6.88228999
H 1.05594079 6.31660445 2.93358123
H 2.43184780 3.17549044 16.66073117
H 0.20699428 5.85057346 1.47372193
H 3.28742399 3.63905781 18.11447181
H 2.26900068 4.59293612 1.62104070
H 1.22408194 4.90146190 17.96489942
H 3.24883356 6.05424359 1.74782574
H 0.24386073 3.43707493 17.86935490
H 2.39753077 5.68420988 0.25195584
H 1.11333991 3.82567723 19.34867773
H 3.02151514 8.24121297 1.23624339
H 0.47082132 1.22927374 18.36543031
H 1.98044121 9.61720847 1.61103759
H 1.51992886 -0.12538185 17.93415943
H 3.40871852 9.10868845 3.51400925
H 0.11766359 0.38893745 16.04002043
H 2.84074814 7.45006764 3.60747053
H 0.57238634 2.08958795 16.02933577
H 1.75976037 8.80979269 3.98814638
H 1.73800317 0.82706022 15.58983411
H -1.13518067 7.10192029 19.11281700
H 4.62585159 2.39660842 0.48496643
H -2.79322114 6.49310079 19.15890365
H 6.28378140 3.00374583 0.43102249
H -2.35293738 8.66988941 17.88016153
H 5.87494944 0.84683788 1.72966955
H -2.17031972 9.48447177 19.42691820
H 5.64188938 0.01330212 0.19660366
H -3.70736129 8.72041614 18.98843691
H 7.19553949 0.76772881 0.58316407
H -0.09161426 8.29848714 3.15195342
H 3.58382464 1.19679218 16.44593162
H -0.23615610 9.43635624 1.82724262
H 3.71876903 0.05258847 17.76612220
H 5.40636195 8.25694128 2.15146066
H -1.91715746 1.22214570 17.44919217
H 6.11903352 6.65926759 2.04681263
H -2.62359391 2.82364531 17.56176703

H 6.00329163 7.69207280 0.60546138
H -2.51462471 1.78412048 18.99735465
C 0.48626279 1.33729983 7.18693415
C 3.17073126 8.11487426 12.31771037
C -0.20816110 3.73758635 6.57949105
C 3.64347803 5.72280437 13.02818080
C 2.20219783 3.35798078 7.69200565
C 1.28011864 6.22184126 11.91733090
C 1.78299525 2.08559150 2.27223630
C 1.73763132 7.47751556 17.32596128
C -0.37695980 3.43088740 3.37482165
C 3.76017118 6.00082925 16.23999249
C -0.15462306 1.00713011 4.01128532
C 3.78528438 8.44917857 15.63313769
C 3.74165841 3.75412944 4.53214714
C -0.44980308 5.56350734 15.10808773
C 3.98214900 2.26312643 4.66048121
C -1.06552176 6.87239725 15.08865131
C -0.34634136 2.54314433 12.82059906
C 3.81537220 6.92256868 6.70884322
C -1.62716296 2.16484012 13.52580971
C 5.09394572 7.25753261 5.98033814
C 0.17478289 0.24600615 12.10041996
C 3.34063571 9.23822127 7.42229242
C -0.42980455 9.39741937 11.12175539
C 3.94157422 0.08704503 8.40106707
C -1.13446248 1.70421566 10.61063570
C 4.65692944 7.77531609 8.89229152
C -1.53618687 3.08077978 10.13702653
C 5.03012111 6.40332136 9.39896305
C 1.25002696 2.20466070 10.97658252
C 2.25682624 7.29795943 8.57287979
C 2.49929943 2.13073024 11.82591503
C 0.99974098 7.37070430 7.73424478
C 1.10080312 6.33813495 1.84757990
C 2.39101399 3.15423972 17.74721035
C 2.33093059 5.64182604 1.33451477
C 1.16640369 3.85589258 18.26519700
C 2.20003275 8.58202354 1.86635919
C 1.28905274 0.91572738 17.71680876
C 2.55207818 8.46258464 3.32956851
C 0.92347815 1.08503320 16.26098151
C -2.14090524 7.30564833 19.47162057
C 5.63007233 2.19060889 0.12392448
C -2.62502449 8.62870665 18.93152141

C 6.11616975 0.87095435 0.66905997
C -0.24831473 8.37893424 2.08030419
C 3.73703668 1.11089137 17.51788142
C 6.19797915 7.67744903 1.67534844
C -2.70650538 1.80407133 17.92704297
N 0.04316384 0.39037134 7.71100056
N 3.67411200 9.01268116 11.76273920
N 2.37417118 1.89734193 1.27787977
N 1.16825201 7.64380305 18.33565344
N -0.00840178 1.67778615 11.63139666
N 3.51239333 7.80548349 7.89517361
N 0.98442693 7.78510500 1.43065306
N 2.50362546 1.70793606 18.16430903
O -1.19346674 4.31860138 6.75399307
O 4.56952523 5.05700049 12.83324058
O 2.82034541 3.76262679 8.58972302
O 0.64333852 5.74372574 11.07147715
O 6.55414599 4.12850602 3.01314761
O -3.26519876 5.23263313 16.64675571
O 6.87416934 0.06073446 4.09109195
O -3.18862997 9.34994925 15.56825611
S 2.46825184 1.37069690 5.16612132
S 1.23017695 8.42094989 14.37442117
S 1.97505015 4.18034690 4.71578225
S 1.43350455 5.44974904 14.84923401
Fe 1.25441287 2.83535043 6.34770210
Fe 2.26889519 6.75641939 13.22873554
Fe 0.88704924 2.38002433 3.90371853
Fe 2.62474335 7.17557993 15.68990427
H 1.99144173 9.48451928 14.03260303
